# Permafrost response to temperature rise in carbon and nutrient cycling: Effects from habitat‐specific conditions and factors of warming

**DOI:** 10.1002/ece3.8271

**Published:** 2021-10-27

**Authors:** Wenlong Gao, Weimin Sun, Xingliang Xu

**Affiliations:** ^1^ National‐Regional Joint Engineering Research Center for Soil Pollution Control and Remediation in South China Guangdong Key Laboratory of Integrated Agro‐environmental Pollution Control and Management Institute of Eco‐environmental and Soil Sciences Guangdong Academy of Sciences Guangzhou China; ^2^ Hainan Key Laboratory of Tropical Eco‐Circular Agriculture Environment and Plant Protection Institute Chinese Academy of Tropical Agricultural Sciences Haikou China; ^3^ Hainan Danzhou Tropical Agro‐ecosystem National Observation and Research Station Danzhou China; ^4^ School of Environment Henan Normal University Xinxiang China; ^5^ Key Laboratory of Yellow River and Huai River Water Environment and Pollution Control Ministry of Education Beijing China; ^6^ Key Laboratory of Ecosystem Network Observation and Modeling Institute of Geographic Sciences and Natural Resources Research Chinese Academy of Sciences Beijing China

**Keywords:** carbon cycling, climate warming, meta‐analysis, permafrost, progressive nitrogen limitation

## Abstract

Permafrost is experiencing climate warming at a rate that is two times faster than the rest of the Earth's surface. However, it is still lack of a quantitative basis for predicting the functional stability of permafrost ecosystems in carbon (C) and nutrient cycling. We compiled the data of 708 observations from 89 air‐warming experiments in the Northern Hemisphere and characterized the general effects of temperature increase on permafrost C exchange and balance, biomass production, microbial biomass, soil nutrients, and vegetation N dynamics through a meta‐analysis. Also, an investigation was made on how responses might change with habitat‐specific (e.g., plant functional groups and soil moisture status) conditions and warming variables (e.g., warming phases, levels, and timing). The net ecosystem C exchange (NEE) was found to be downregulated by warming as a result of a stronger sensitivity to warming in respiration (15.6%) than in photosynthesis (6.2%). Vegetation usually responded to warming by investing more C to the belowground, as belowground biomass increased much more (30.1%) than aboveground biomass (2.9%). Warming had a minor effect on microbial biomass. Warming increased soil ammonium and nitrate concentrations. What's more, a synthesis of 70 observations from 11 herbs and 9 shrubs revealed a 2.5% decline of N in green leaves. Compared with herbs, shrubs had a stronger response to respiration and had a decline in green leaf N to a greater extent. Not only in dry condition did green leaf N decline with warming but also in wet conditions. Warming in nongrowing seasons would negatively affect soil water, C uptake, and biomass production during growing seasons. Permafrost C loss and vegetation N decline may increase with warming levels and timing. Overall, these findings suggest that besides a positive C cycling–climate feedback, there will be a negative feedback between permafrost nutrient cycling and climate warming.

## Graphical abstract

This work provides a quantitative and mechanistic basis for predicting the functional stability of permafrost C and N cycling under warmer climates.

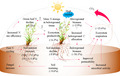

## 1 INTRODUCTION

**FIGURE 1:**
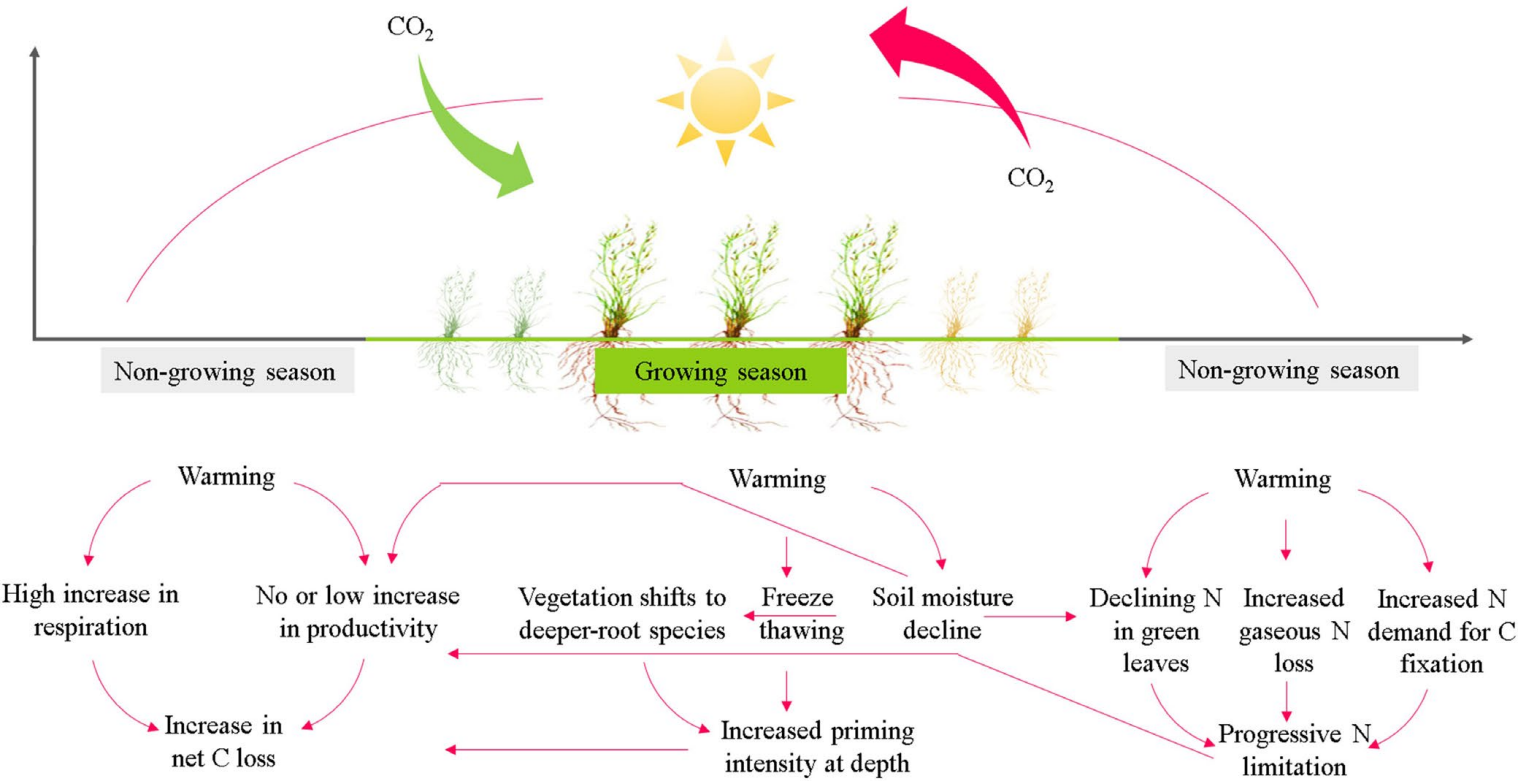
The potential pathways through which climate warming stimulates net C loss from permafrost ecosystems. Initially, rising temperature can stimulate massive SOC loss and productivity in permafrost ecosystems due to enhanced mineralization caused by increased microbial activity (Xue et al., 2016). With timing, the SOC loss is to enlarge when freeze–thawing induces a “biotic awakening” at depth where the majority of permafrost C is stored (Sistla et al., 2013), or microbial decomposition of organic C in subsoil is accelerated after a shift vegetation toward species with deeper roots caused by reduced soil moisture (Liu, Q., et al., 2018). Ongoing warming is to weaken the functional role of a permafrost ecosystem in C uptake through progressive nitrogen (N) limitation (Kou et al., 2020)

Terrestrial ecosystem carbon (C) dynamics and its feedback to climate changes are the most concerned issues in global change ecology (Greaver et al., 2016; Quan et al., 2019). The northern permafrost regions (15%–16% of global land area) are the largest terrestrial C pool, which store 1.5–2.0 times the amounts of C (1300–1672 Pg C) present in the atmospheric carbon dioxide (Köchy et al., 2015; Schuur & Mack, 2018; Tarnocai et al., 2009). Unfortunately, they are warming at a rate (0.20–0.29°C per decade) that is two times faster than the rest of the Earth's surface (Biskaborn et al., 2019; Chen et al., 2013; Schuur et al., 2015). Due to the high climate sensitivity, there is a concern that permafrost regions are soon to cross a “tipping point” where climate warming turns the areas from a net C sink to a net C source, thereby acting as an amplifier of climate change (Chen et al., 2013; Koven et al., 2011; Turetsky et al., 2019; Xue et al., 2016). It has been estimated that in decades to come, permafrost soils can lose up to 5%–15% of their current C storage (Schuur et al., 2015; Tan et al., 2010). The potential pathways through which climate warming stimulates permafrost C loss are summarized in Figure 1. However, the mechanisms we summarized are usually ecosystem‐specific, making it difficult to integrate them into the coupled carbon–climate models (Charles et al., 2015; Chen et al., 2013; Luo, 2007).

Moreover, how fast such a tipping point is near depends also on productivity response (Xue et al., 2016). In the Northern Hemisphere, warming usually enhances C gain of an ecosystem by extending the growing season (Liu, H., et al., 2018; Liu, Q., et al., 2018; Zhang et al., 2013) to increase vegetation photosynthesis and biomass (Chen et al., 2020; Wang et al., 2019). In addition, photosynthesis could be favored by increased CO_2_, which is termed as CO_2_ fertilization (Körner, 2001; Luo, 2007). Notably, permafrost vegetation has evolved many adaptive strategies (e.g., improved plant functional traits in heights and leaf areas, and a shift of vegetation toward species that are high in heat stress tolerance, water, and N use efficiencies) to maintain its functional stability in C uptake (Bjorkman et al., 2018; Liu, H., et al., 2018; Liu, Q., et al., 2018; Wookey et al., 2009). Thus, it remains still an open question whether permafrost C–climate feedback is positive (Chen et al., 2013; Xue et al., 2016), since there is no clear answer to how net C exchange (NEE) in alpine and arctic regions would change with their biological controls such as productivity and respiration (Table S1).

Numerous studies have projected that alpine or arctic NEE is found to be downregulated by warming as a result of a stronger sensitivity to warming in respiration than in photosynthesis (Heimann & Reichstein, 2008; Welker et al., 1999). Many mechanisms can contribute to a stronger respiration response to warming. For one thing, under warmer conditions alpine and arctic vegetation generally increases their C allocation to the belowground (Chen et al., 2020; Liu, H., et al., 2018; Liu, Q., et al., 2018). This is to increase flows of fresh and liable substrates to the rhizosphere to increase priming intensity (Keuper et al., 2020; Xue et al., 2016; Zhang et al., 2015) and will induce a “biotic awakening” at depth (Liu, H., et al., 2018; Liu, Q., et al., 2018; Sistla et al., 2013). For another, warming can increase availability of nutrients through enhancing mineralization of soil organic matter, increasing microbial respiration due to stimulating microbial growth (Lu et al., 2018; Zhang et al., 2015).

Progressive N limitation could be a cause for a lower sensitivity of photosynthesis than of respiration to warming (Luo et al., 2004). For example, since plant demand for N could be far beyond soil N supply (Luo, 2007), plant growth and associated C fixation are still N‐constrained in many permafrost sites where soil bioavailable N increased with warming (Chen et al., 2020; Salazar et al., 2020; Zhang et al., 2015). Besides, alpine or arctic plants exposed to warming often demonstrate lower N concentrations in green leaves (Li et al., 2017; Michelsen et al., 1996; Welker et al., 2004), which is often regarded as a key determinant to photosynthetic C fixation (An et al., 2005). Under warming conditions, green leaf N decline could be ascribed to a dilution effect from higher biomass production caused by increased N use efficiency (An et al., 2005) and is likely to feed back to aggravate N limitation by slowing down ecosystem N turnover through increasing production of low‐quality litter (Gao & Yan, 2019). As suggested by a model simulation, if progressive N limitation occurs, then more likely a permafrost ecosystem functions as a net C source (Charles et al., 2015). Thus, to better predict permafrost C–climate feedback, it is critical to understanding how vegetation biomass, microbial biomass, leaf N status, and soil N availability in alpine, subarctic, and arctic ecosystems respond to warming (Kou et al., 2020; Mao et al., 2020; Salazar et al., 2020). However, there are some uncertainties about the direction and magnitude of warming effects on these parameters (Table S1). Because habitat‐specific conditions are a major contributor to the variation in permafrost C and N responses (Greaver et al., 2016; Liu, H., et al., 2018; Liu, Q., et al., 2018; Quan et al., 2019), there is an urgent demand for identifying their influences to better predict the permafrost sensitivity to climate warming in C and N cycling.

Climate sensitivity of permafrost C and nutrient cycling is highly linked to habitat‐specific conditions such as plant functional groups and soil moisture status (Liu, H., et al., 2018; Liu, Q., et al., 2018; Wookey et al., 2009). For instance, as a matter of fact, shrubs compared with herbs with a larger coverage, height, and standing biomass are higher in N (biomass production per unit of vegetation N) and water use efficiencies (Sistla et al., 2013; Wookey et al., 2009). Although these traits may enable shrubs to stabilize primary production under warming, they also create conditions to stimulate a net C loss from an ecosystem by inducing a strong respiration response through additional inputs of labile C to the belowground and to deeper soils (Sistla et al., 2013). Moreover, organic matter from shrubs is more lignified with a high lignin‐to‐N ratio, making N turnover of the ecosystem too slow to supply plants quickly with nutrients (Wookey et al., 2009). Globally, soil moisture status is a key regulator of terrestrial C and N cycling (Greaver et al., 2016). If soil moisture becomes a limiting factor to plant productivity, soil N mineralization, and plant N uptake, a positive effect of warming on C uptake of an ecosystem could be tipped (Quan et al., 2019). Nonetheless, it remains unclear how permafrost C–climate and N–climate feedbacks vary with plant functional groups and soil moisture status.

Additionally, warming variables including patterns, levels, and timing strongly affect the relationships of permafrost C and nutrient cycling with climate (Chen et al., 2013; Kou et al., 2020; Luo, 2007; Natali et al., 2019). When temperature increase occurs in nongrowing seasons (Natali et al., 2019; Zhang et al., 2019), winter permafrost degradation will increase water, drought, and nutrient stresses on ecosystems in summers (Soja et al., 2007). Along with the increase in warming levels and timing, water, drought, and nutrient stresses on ecosystems are likely to increase (Greaver et al., 2016; Liu, H., et al., 2018; Liu, Q., et al., 2018; Ma et al., 2017). Although the effects derived from warming patterns, levels, and timing on permafrost C and N cycling have been examined by a number of field experiments, their general patterns are rarely synthesized through a meta‐analysis. This impedes an incorporation of these factors into models for evidence‐based predictions.

As global warming trend is likely to continue in coming decades (Delmotte et al., 2018), there is a need to predict the functional stability of permafrost ecosystems in C and nutrient cycling. To achieve this, we used a meta‐analysis to characterize the general effect of warming on the C exchange, vegetation growth, microbial biomass, leaf N status, and soil N availability in alpine, subarctic, and arctic ecosystems. Unlike previous meta‐analyses that focused merely on one of the two cold‐climate zones (Chen et al., 2020; Liu, H., et al., 2018; Liu, Q., et al., 2018; Lu et al., 2013; Rustad et al., 2001; Zhang et al., 2015), we compiled data from alpine and arctic biomes both that were similar in mean climate and vegetation (Myers‐Smith et al., 2015; Wang et al., 2014). Especially, we investigated how the effects may change with habitat‐specific conditions and factors of warming, which are lacking in previous syntheses (Chen et al., 2020; Liu, H., et al., 2018; Liu, Q., et al., 2018; Lu et al., 2013; Zhang et al., 2015). Based on above‐mentioned concerns, we aim to test the following hypotheses: (1) warming would induce a downregulation of NEE in cold biomes mainly by stimulating a larger biological response to respiration than to photosynthesis; (2) warming increased soil N availability in cold biomes, but the response of green leaf N to warming would be negative as a result of a dilution effect from higher biomass production; (3) temperature effects on the C exchange, C allocation, and leaf N would vary with plant functional groups and soil moisture status as well as warming variables.

## 2 MATERIALS AND METHODS

### 2.1 Data collection

To clarify the responses of permafrost to climate warming, we mainly focused on warming experiments conducted in the Northern Hemisphere. Boreal forests are excluded due to a few warming experiments (Allison & Treseder, 2008; Bergner et al., 2004; Bronson et al., 2008; Lavoie et al., 2015; Niinistö et al., 2004), as well as low vegetation similarity compared with alpine, subarctic, and arctic ecosystems (Myers‐Smith et al., 2015). Air‐warming, rather than soil‐warming, response was taken into account, mainly because of its representativeness including its direct effect on aboveground parts.

Fifteen response variables were selected, including gross ecosystem production (GEP), ecosystem respiration (ER), net ecosystem exchange (NEE), aboveground biomass (AGB), aboveground N pool (AGN), green leaf N, leaf δ^15^N, belowground biomass (BGB), microbial biomass (MB), soil temperature, soil moisture, soil organic C (SOC), soil total N, soil NH_4_^+^, and soil NO_3_^−^ (Tables S2 and S3). We searched publications from Web of Science and Google Scholar published before July 2019.

The terms “warming” & “permafrost” or “temperature” & “permafrost” were used for searching. Studies were selected for meta‐analysis if they reported on (1) air‐warming experiments in alpine, subarctic, and arctic biomes; (2) air‐warming approaches of infrared radiator (IR), open‐top chamber (OTC), snow fences, or greenhouse, rather than soil heating cables; (3) field observations from control and warming treatments during the growing season; and (4) mean, standard error (or deviation), and sample sizes.

GEP and ER were expressed in positive values, with NEE > 0 and NEE < 0 indicating net C gain and C loss, respectively. Data on green leaf N and leaf δ^15^N were collected by species (Table S3). As for belowground variables, only the top layer was included for analysis if various soil layers were surveyed (Table S2). Except for BGB (0–40 cm), the data collected for other belowground variables were confined to 0–20 cm (Table S2). Information on the unit, soil layer, experimental location, mean growing‐season air temperature (1.4–17.4°C), mean growing‐season rainfall (30–600 mm), plant function groups, soil moisture status, warming pattern, warming level (air temperature increase), and warming time (in growing seasons) was collected (Tables S2 and S3). Data were extracted using Engauge Digitizer 9.0 if they were presented in figures.

**FIGURE 2:**
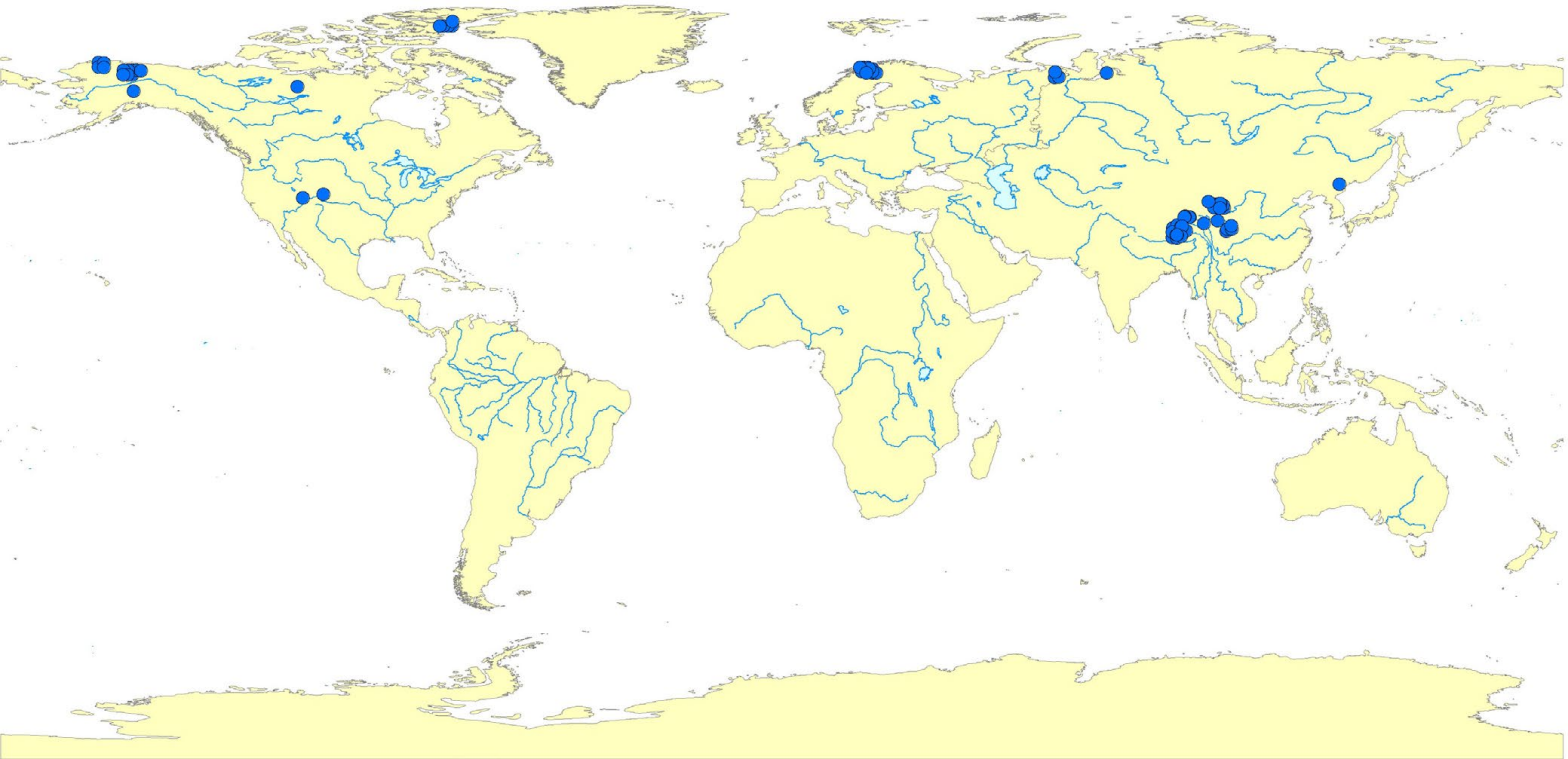
Warming experiments conducted in alpine, subarctic, and arctic biomes of the Northern Hemisphere

Meta‐analysis models require independence among observations (Hedges et al., 1999). Therefore, for studies having several measurements over time within a single growing season at the same warming level, overall means were pooled as a single observation. Measurements from different vegetation types, soil moisture status, warming patterns, warming levels, and warming time within a single study were viewed as independent variables. In total, 100 publications were included, with 708 observations from 89 warming experiments located in the world map (Figure 2). The 89 warming experiments were independent of each other.

To test the differences in responses between plant functional groups, vegetation types were classified into mosses, herbs, herbs and shrubs, and shrubs. Soil moisture status was grouped into dry, moist, and wet. Because of few sites where soil moisture status was not specified (Fu et al., 2019), we characterized it according to the ratio of soil moisture content to water holding capacity (<60%: dry, 60%–100%: moist, >100%, wet). As reports on nongrowing warming effects in cold biomes were rather limited (Fu et al., 2019, Zong et al., 2018), they were not included for an analysis. Air temperature increases varied from 0.2 to 6.2°C, with 2°C chosen as the subdividing point (low warming levels: ≤2℃, high warming levels: >2℃). The warming length ranged from 1 to 21 growing seasons and was grouped into short‐ and long‐term warming subdivided by 3 growing seasons.

Admittedly, warming approaches may affect warming effects (Wang et al., 2019). Unlike IR, passive warming, such as OTC, is hard to maintain a fixed temperature varying from few to several degrees along the day and increases temperature only during daytime. Moreover, passive warming of open‐top chamber could reduce photosynthetically active radiation (Boelman et al., 2003). However, a recent study has confirmed that warming methods (e.g., IR and OTC) have no differences in their effects on permafrost GEP, ER, NEE, AGB, BGB, SOC, MB, and soil total N (Chen et al., 2020). Thus, warming approach effects were not considered in this study.

### 2.2 Meta‐analysis

As for NEE and leaf δ^15^N, they showed negative values in either control or warming treatments. Thus, their effect sizes were evaluated using the “Hedges’ *d* index” (Gao & Yan, 2019; Rustad et al., 2001). For the remaining thirteen response variables, response ratio, OR = *X_t_*/*X*_c_ was used as a metric to estimate effect sizes (Hedges et al., 1999). Log transformation of OR was done (In OR), and its variance (v) was computed by following equation (Hedges et al., 1999):

$$v=S_{c}^{2}/n_{c}X_{c}^{2}+S_{t}^{2}/n_{t}X_{t}^{2}$$

where *X_t_*, *S_t_*, and *n_t_* denote the mean, standard deviation, and sample size in the experimental group, and *X_c_*, *S_c_*, and *n_c_* denote the mean, standard deviation, and sample size in the control group. The weighting factor, the weighted mean of In OR, and its standard error were calculated as described in Hedges et al. (1999). Mean response ratio (OR) and its 95% confidence interval (CI) were gained by taking antilog. To quantify variations, the mean effect size was then transformed back to percentage change.

All meta‐analyses were performed using stata 14.0 (StataCorp, Texas, USA). A fixed‐effects model using inverse‐variance pooling was selected. Publication bias was assessed with Egger's test, with results showing a lack of publication bias (see Table S4). Admittedly, there were differences in results between Begg's and Egger's tests. Compared with Egger's test, Begg's test was more sensitive (see Table S4). Despite this, the results from Egger's tests are presented in Table S4.

For each response variable, we first calculated the overall response of the whole dataset and then examined between‐group heterogeneity (*Q_b_*, Table S5) through a *Q* test (Liu & Greaver, 2009). For response variables (total observations >10) with a significant *Q_b_*, subgroup analysis (vegetation type, soil moisture status, warming pattern, warming level, and warming time) was conducted. If the 95% CI of percent change or the “Hedges’ *d* index” did not overlap 0, the response was considered to be significant.

To examine how climate factors (e.g., mean growing‐season air temperature and rainfall) affected warming response, linear regression with Durbin–Watson test was performed. Significance was tested at *p* < .05.

## 3 RESULTS

### 3.1 Responses to warming in C exchange and balance

**FIGURE 3:**
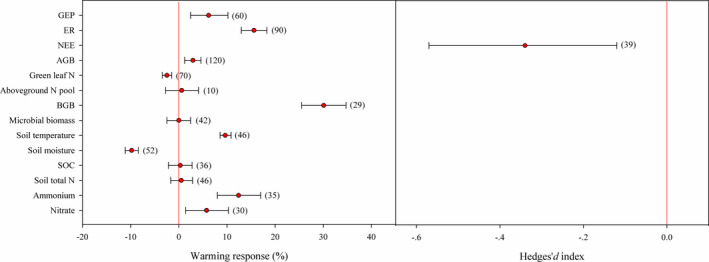
Overall mean effect sizes and 95% confident intervals, with the observations indicated by numbers in brackets. GEP: gross ecosystem productivity, ER: ecosystem respiration, NEE: net ecosystem C exchange, AGB: aboveground biomass, BGB: belowground biomass, SOC: soil organic C

**FIGURE 4:**
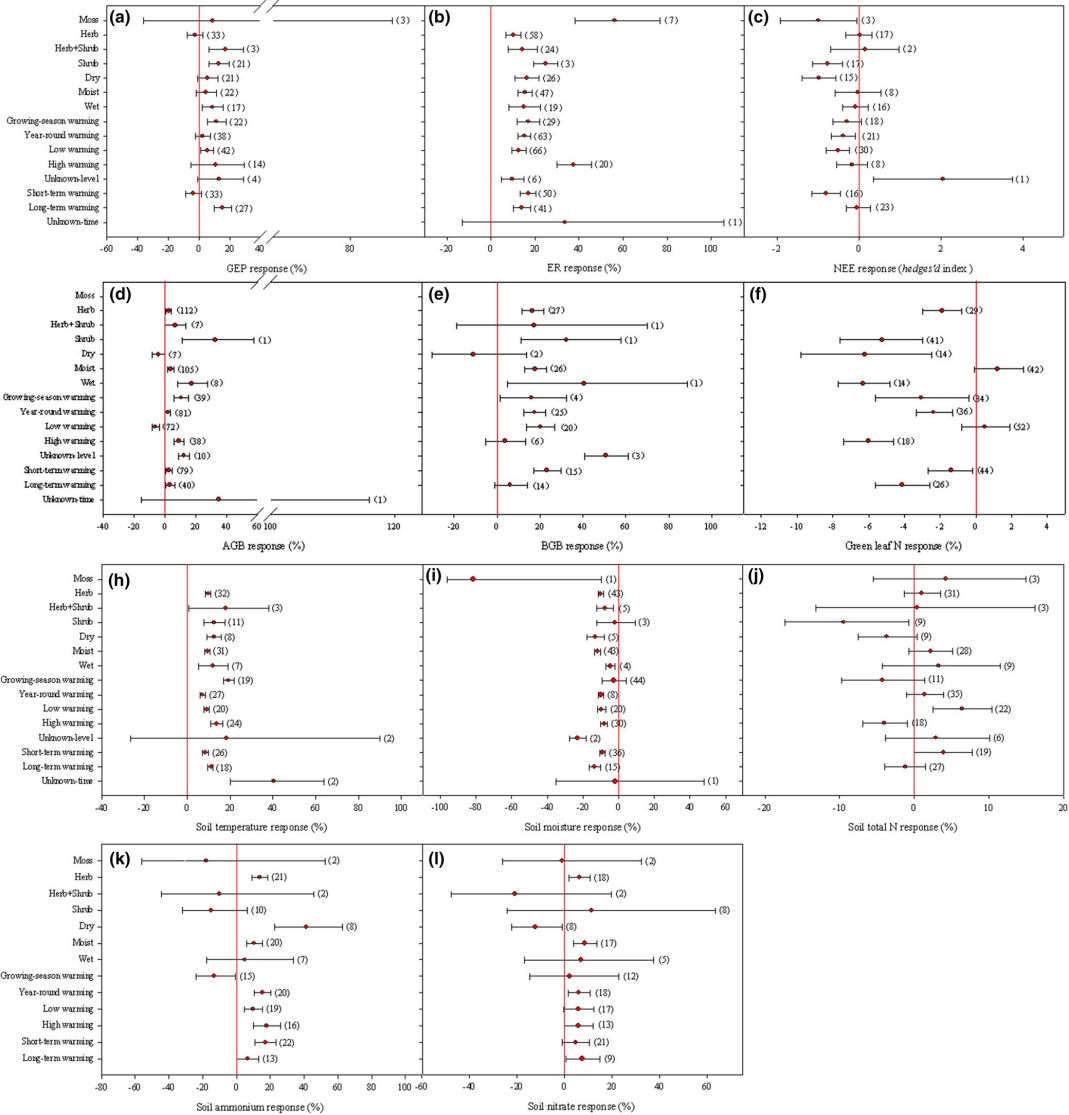
Mean effect sizes and 95% confident intervals based on plant functional groups (mosses, herbs, herbs and shrubs, and shrubs), soil moisture status (dry, moist, and wet), warming patterns (growing‐season warming and year‐round warming), warming levels (low and high warming levels), and warming time (short‐term and long‐term warming). Low warming levels:≤2℃, high warming levels: >2℃; short‐term: no more than 3 growing seasons, long‐term warming: more than 3 growing seasons

Warming downregulated NEE during growing season (Hedges’ *d* index: −0.34, 95% CI: −0.57 to −0.12) as a result of a larger biological response to respiration than to uptake (Figure 3). On average, GEP was enhanced by warming of 6.2% (95% CI: 2.43–10.19%), while the increase in ER was up to 15.6% (95% CI: 12.98–18.29%, Figure 3). Warming effects on NEE varied with vegetation types (*p* < .001, Table S5). NEE decline with warming was evident in moss or shrub communities that had a stronger respiration response than herbaceous communities (Figure 4a–c). Also, NEE response to warming was regulated by soil moisture status and warming time, which declined under dry condition or short‐term time treatment (Figure 4c). Warming patterns affected GEP response (Table S5), with no positive effects from year‐round warming (Figure 4a). High warming levels stimulated a higher increase in ER than low warming levels (Figure 4b). NEE was not downregulated by long‐term warming that had a stimulation on GEP (Figure 4a,c). Interestingly, warming response of NEE, as well as GEP, was negatively correlated to growing‐season rainfall (Figure S1).

### 3.2 Vegetation response to warming

Vegetation responded to experimental warming of 0.5–5.4℃ for 1 to 20 growing seasons by a small increase in AGB (mean response: 2.94%, 95% CI: 1.21–4.60%) but a large increase in BGB (mean response: 30.08%, 95% CI: 25.48–34.72%, Figure 3). Unlike herb communities, data were lacking to estimate biomass response for shrub communities (Figure 4d,e). When analyzed by warming patterns, AGB was not usually to respond positively under year‐round warming (Figure 4d). In addition, warming effects on vegetation biomass were temperature‐dependent (Table S5). Low warming levels increased BGB but decreased AGB, while high warming levels increased AGB without effects on BGB (Figure 4d–f). Unlike AGB, BGB response varied over time (Figure 4f).

Aboveground N storage did not increase with AGB (Figure 3). In contrast to leaf δ^15^N (Figure S2), a synthesis of 29 observations from 11 herbs and 41 observations from 9 shrubs revealed a 2.47% decline in green leaf N (95% CI: −3.44 to −1.49%, Figure 3). Compared with herbs, shrubs showed a higher reduction in green leaf N with warming (Figure 4f). Negative impacts of warming on green leaf N were common in dry and wet conditions (Figure 4f). High warming levels that had AGB a significant increase induced green leaf N a large decline (Figure 4f). An increase in warming time would bring green leaf N down further (Figure 4f).

### 3.3 Soil and microbial responses to warming

In contrast to soil temperature (9.64%), soil moisture was often reduced by warming (−9.80%) during growing seasons. Normally, soil moisture declined more in nonwet conditions (e.g., moist and dry conditions), or under year‐round and long‐term warming (Figure 4i). Microbial biomass usually exhibited undetectable changes in warmer climates (Figure 4). Warming generally increased soil nutrients, although no increase in soil total N was observed (Figure 3). Positive responses of soil nutrients to warming relied highly upon vegetation types. Under warming, soil nutrients increased in herbaceous communities, but it was not the case in shrub communities where soil total N tended to decrease (Figure 4j,l). Although there was a decrease of soil NO_3_^−^, soil NH_4_^+^ increased largely in dry conditions (Figure 4k, i). Soil NH_4_^+^ decreased under growing‐season warming, but increased under year‐round or long‐term warming. No temperature‐ and time‐dependent responses were observed for soil NH_4_^+^ and NO_3_^−^ (Figure 4k,i).

## 4 DISCUSSION

### 4.1 General effects of warming on permafrost C and nutrient cycling

Net C exchange determines if an ecosystem acts as a net source or sink for C, and thus, understanding its response has important implications for modeling and predicting terrestrial C–climate feedback (Heimann & Reichstein, 2008; Luo, 2007). As indicated by Hedges'd index (mean response: −0.34, 95% CI: −0.57 to −0.12), warming effect on alpine or arctic net C uptake could be often negative. This is contrasted with previous meta‐analyses that reported a neutral to positive effect using a small database (*n* = 6–18, Table S1) (Chen et al., 2020; Lu et al., 2013; Wang et al., 2019). Confirming our first hypothesis, NEE downregulation would be mainly emission‐driven, since biological response could be much stronger in respiration (e.g., ER, 15.6%) than in uptake (e.g., GEP, 6.2%, Figure 3). Considering the observations of an extended growing season (Liu, H., et al., 2018; Liu, Q., et al., 2018; Zhang et al., 2013), to verify if climate warming is to weaken the functional role of alpine and arctic ecosystems as a net C sink, further investigations should be done to answer the question as follows. Whether the increase in productivity in the extended period can compensate increased respiration or increased respiration in the nongrowing seasons exceeds increased productivity in growth seasons?

Our prediction in ER increase is approach to an earlier estimate (15.0%) (Chen et al., 2020). Such a large increase in ER is not surprising, since as a key component of ER, soil respiration is enhanced by an average of 11.3%–14.3% after experimentally increasing temperature to alpine or arctic ecosystems (Chen et al., 2020; Lu et al., 2013). Although many studies have attributed increased soil and ecosystem respiration to autotrophic respiration, plant respiration is not considered to be a positive feedback to climate warming. The reason is that plant respiration could be balanced by production over time, whereas microbial respiration cannot be balanced (Pries et al., 2016). Therefore, the central to terrestrial C–climate feedback is soil microbial response (Jansson & Hofmockel, 2020; Pendall, 2018; Xue et al., 2016).

Our meta‐analysis (mean reason: 0, 95% CI: −2.47%–2.43%, Figure 3), together with two previous syntheses (Chen et al., 2020; Salazar et al., 2020), suggests that warming has a low potential to increase microbial biomass in alpine or arctic soils. It may be the case. For example, after a synthesis of data from cold ecosystems of high northern latitudes (>50°), Salazar et al. (2020) observed no evidence of bacterial and archaeal abundances affected by experimental warming, though they pointed out that fungi would respond positively to warming.

GEP is a critical process controlling C gain of an ecosystem. Our meta‐analysis revealed that GEP–climate relationship was positive (6.2%, Figure 3). A previous study also showed that in a warmer climate, alpine or arctic NPP (an index that subtracts respiration from gross production) is to increase (e.g., 23.6%) (Wang et al., 2019). However, empirical evidence has suggested that in cold‐climate zones, aboveground NPP (−1.5%–20.5%) would be not so positive in response to warming as belowground NPP (19.0%) (Liu, H., et al., 2018; Liu, Q., et al., 2018; Wang et al., 2019). Nonetheless, increased aboveground and belowground C storage may be evident, as parallel to GEP dynamics, AGB and BGB typically increased in warming experiments (Figure 3). Similar to Chen et al. (2020), our meta‐analysis revealed that alpine or arctic vegetation would invest more C to the belowground under warming, as BGB (30.1%) compared with AGB (2.94%) exhibited a larger increase (Figure 3).

Our meta‐analysis of 11 herbs and 9 shrubs revealed that even if soil N availability increased with warming, N in green leaves may decline, showing consistence with second hypothesis (Figure 3). Similar results were also obtained in temperate biomes (Gao & Yan, 2019). These findings indicate that climate warming could exert consistent impact on global biomes (Craine et al., 2018), and reduce green leaf N in a broader climatic or geographical gradient. Green leaf N decline may be not in favor of C fixation, as in a Tibetan permafrost ecosystem subjected to warming, reduced N in the leaves of dominant plants of *K*. *tibetica* and *C*. *atrofusca* weakened GEP (Li et al., 2017).

However, it is still a challenge to judge if progressive N limitation is to occur under green leaf N decline. Two studies have addressed such a concern by making an investigation on vegetation δ^15^N (Craine et al., 2018; Kou et al., 2020). This is related to two reasons (Kou et al., 2020): (1) in permafrost zones where atmospheric N deposition and biological N fixation have a limited effect, vegetation δ^15^N would decline with warming over time; (2) decreased vegetation δ^15^N is a sign of increased reliance on mycorrhizae, which is crucial for transferring ^15^N‐deleted N from soils to plants under N‐limited conditions. Thus, data were collected from 11 species to evaluate warming effect on leaf δ^15^N, with results showing a lack of an alternation (Figure S2).

### 4.2 Mechanisms controlling permafrost response to warming in C and nutrient cycling

**FIGURE 5:**
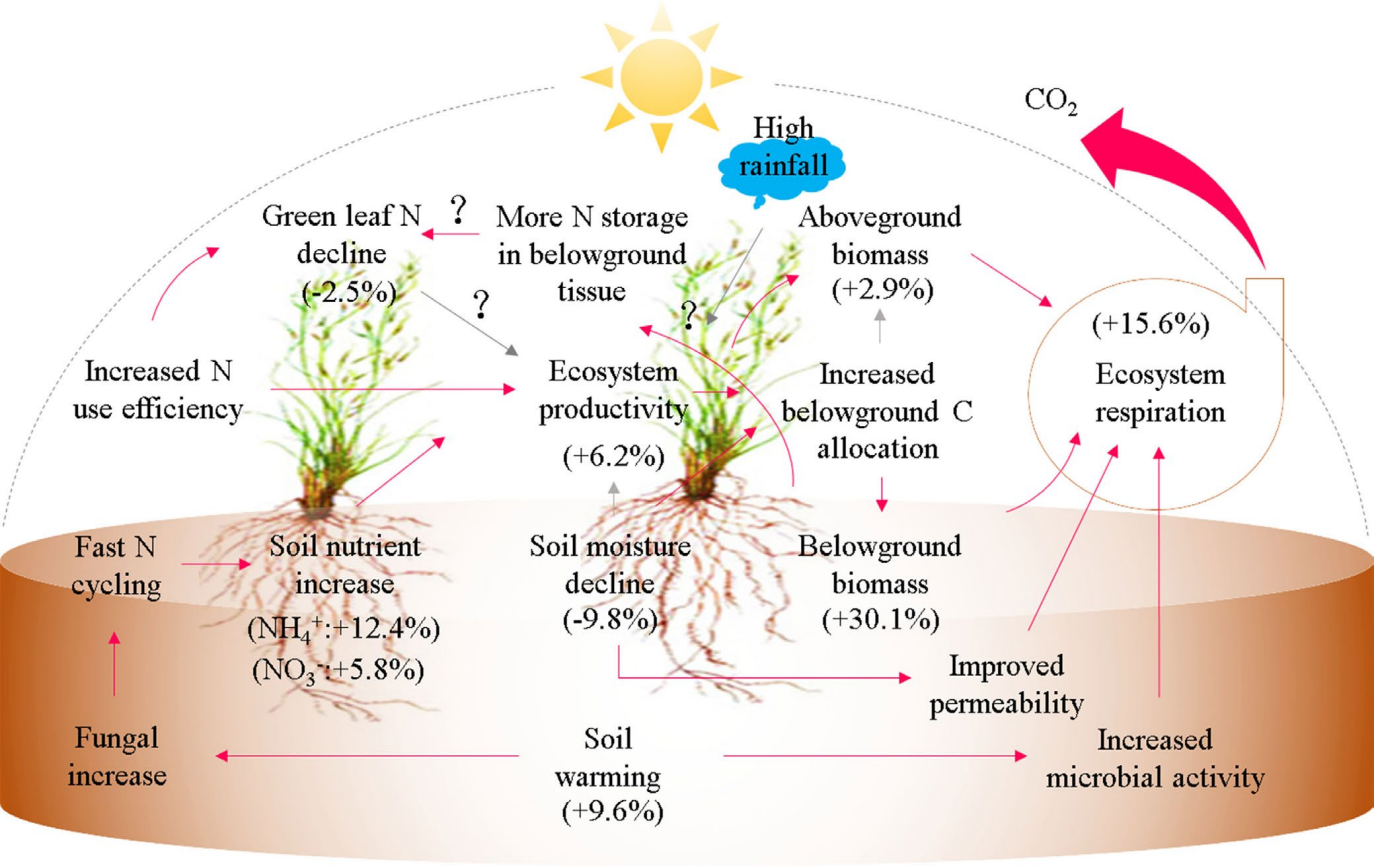
Mechanisms controlling permafrost response to warming in C‐ and nutrient cycling. According to Salazar et al. (2020), an increase in fungal dominance, rather than bacterial and *amoA* gene abundances, is likely to drive a fast turnover of N in permafrost soils after warming. Increased N turnover, together with a lack of more N locked up by vegetation as reflected by no increase in aboveground N pools and green leaf N, will lead to increased N availability in soils. As vegetation productivity and growth in cold biomes are generally co‐limited by temperature and nutrient (Körner, 2001; Natali et al., 2012), increased soil N availability under warmer climates is to stimulate GEP. Increased GEP could be a contributor to increased vegetation biomass production. It may be that to cope with stresses from water, alpine and arctic vegetation increases their C allocation to the belowground (Liu, Q., et al., 2018). Under increased GEP, AGB, and BGB, fresh and liable substrate flows from plants to soils increase (Xue et al., 2016; Zhang et al., 2015), increasing priming intensity in the rhizosphere (Keuper et al., 2020). This may stimulate a larger biological response to respiration than to uptake, leading to a downregulation of NEE. Note that microbial biomass may be not a good indicator of respiration activity in permafrost, as whose response to warming is generally insensitive. Increased C fixation and biomass production may amplify tissue C and N imbalance, since green leaf N declines as AGB increases (An et al., 2005, Gao & Yan, 2019). It remains to be seen if green leaf N decline will be also associated with increased N allocation to the belowground and is to negatively affect long‐term C fixation. Red lines: stimulatory effects, black lines: inhibitory effects

Mechanisms controlling permafrost C–climate and nutrient–climate relationships are summarized in Figure 5. When alpine or arctic ecosystems are exposed to warming, NEE decline could be a result of diminished photosynthesis along with stimulated respiration (Voigt et al., 2017), or a greater inhibition on productivity than on respiration (Li et al., 2019). Our meta‐analysis confirms the finding of Oberbauer et al. (2007) that respiration response typically determines warming effects on C balance of a permafrost ecosystem (Figure 5). In cold biomes subjected to warming, there are many factors contributing to a strong respiration response (Figure 5). In addition to temperature, increased GEP, AGB, and BGB also serve as an important driver (Figure 3), since they are important controls of priming intensity in the rhizosphere due to their effects on labile substrate flows from plants to soils (Keuper et al., 2020; Liu, H., et al., 2018; Liu, Q., et al., 2018; Xue et al., 2016; Zhang et al., 2015). According to Oberbauer et al. (2007), at wet alpine or arctic sites, reduced soil moisture after warming would lead to an improved permeability to facilitate both an increase in below‐ and aboveground respiration. However, this meta‐analysis, combined with synthesized results from Chen et al. (2020) and Salazar et al. (2020), suggests that increased respiration activity (e.g., heterotrophic respiration) in warmed alpine or arctic sites is unlikely a result of microbial biomass increase (Figure 3). The lack of a response to microbial biomass may indicate that microbial C use efficiency in cold biomes is not to increase with warming (Figure 3).

In permafrost zones where vegetation productivity and growth are co‐limited by temperature and nutrients (Körner, 2001; Natali et al., 2012), increased temperature and nutrients could be a contributor to increased GEP (Figure 3). Increased N use efficiency, as reflected by increased AGB under green leaf N decline (Figure 3), could be also a mechanism leading to increased GEP under warming (An et al., 2005). In addition, increased GEP is likely associated with other factors, for example, improved plant functional traits in canopy and height (Ganjurjav et al., 2016; Oberbauer et al., 2007). This could be a case, as in a Tibetan permafrost site, gross ecosystem photosynthesis response to warming (ΔGEP) was positively linked with not only soil temperature but also normalized difference vegetation index (Li et al., 2017). Unexpectedly, compared with the global average (16.3%, Greaver et al., 2016), the stimulation of GEP by warming is much smaller in cold‐climate zones (6.2%, Figure 3). This is not surprising, considering that water availability may dominate over temperature in shaping alpine or arctic C fixation during growth seasons, and its decline would produce a negative effect (Fu et al., 2019). Moreover, at permafrost sites with a high rainfall during the growth seasons, GEP would not increase but decrease in response to warming (Figure S1), reflecting the complexity of warming effects on permafrost ecosystems.

Recent studies have highlighted that even if there are stresses from water under warming, alpine and arctic ecosystems could maintain their functional stability in productivity through shifting vegetation toward species with deeper‐root systems (Liu, H., et al., 2018; Liu, Q., et al., 2018). As revealed by Liu, H., et al. (2018) and Liu, Q., et al. (2018), alpine grasslands exhibited an increase in grass abundance that had deeper roots (up to 85 cm soil depth) at the expense of sedge (up to 25 cm) or forb (up to 30 cm) after a 4‐year manipulative experiment of warming and drought (50% reductions in precipitation). This provides an explanation for why aboveground primary production could stabilize under climate change, and why belowground compared with aboveground biomass is usually higher in increase with warming (Figure 3). As a result of increased belowground C allocation (e.g., BGB, Figure 3), there is a growing concern over if ongoing warming would induce a “biotic awakening” at depth to weaken the role of cold biomes functioning as a net C sink (Liu, H., et al., 2018; Liu, Q., et al., 2018; Sistla et al., 2013).

The dilution effect from biomass production is often suggested to be a reason for green leaf N decline (An et al., 2005; Luo et al., 2004; Wan et al., 2005). It may be the case, since aboveground N pool did not appear to increase with AGB when alpine and arctic vegetation were subjected to warming (Figure 3). The larger increase in BGB compared with AGB raises such a question. Could green leaf N decline under warming be a result of increased N allocation to the belowground tissues to enhance root functions for the uptake of water and nutrients? (Figure 5). As reported, green leaf N decline under warming could be also linked to decreased N resorption (less N is transported from senescent to alive leaves), chlorophyll degradation, and advanced plant senescence (Li et al., 2017; Prieto & Querejeta, 2020; Shi et al., 2015). If vegetation is long‐live or inherently high in lignin‐to‐N ratio (e.g., shrubs), green leaf N decline with warming is more likely to occur due to the slow in ecosystem N turnover (Gao & Yan, 2019; Luo et al., 2004; Wookey et al., 2009; Zhou et al., 2011). However, a lack of enough data cannot allow us to identify which mechanisms are mainly responsible for green leaf N decline. Because foliar N content is tightly related to photosynthesis and thus biomass production, further investigations should be done to clarify it for better understanding warming effects on alpine and arctic ecosystems.

In contrast to Rustad et al. (2001) and Zhang et al. (2015), our meta‐analysis, together with other two syntheses (Chen et al., 2020; Salazar et al., 2020), highlights that microbial biomass is not often to increase to fuel N mineralization in permafrost soils, as whose temperature sensitivity is generally low (Figure 3). As revealed by Salazar et al. (2020), if alpine and arctic are exposed to warming, increased rates of N production appear to be more linked to increased urease and protease activity and increased fungal dominance, since bacterial and *amoA* gene abundances are often lacking in increase. There is a suggestion that under a warmer climate, alpine and arctic vegetation would increase their reliance on mycorrhizae (Kou et al., 2020) or fungi (Bragazza et al., 2013; Deslippe et al., 2012; Salazar et al., 2020) for N acquisition.

### 4.3 Factors affecting permafrost response to warming in C and nutrient cycling

In support of the third hypothesis, plant functional groups could be an important factor affecting warming effects on permafrost C exchange (e.g., GEP, ER, and NEE) and green leaf N (Figure 4, Table S5). In the current study, we paid a special attention to herbaceous and woody (e.g., shrub) communities, as they are the two dominant vegetation forms in alpine and arctic zones (Wookey et al., 2009). Probably due to a stronger respiration response, NEE downregulation with warming is often evident in shrub communities (Figure 4b,c). Since autotrophic and heterotrophic respiration both depend highly on C substrates provided by photosynthesis, their differences in respiration response could be related to GEP dynamics (Liang et al., 2013). Compared with herbaceous communities, warming is more favorable for shrub communities to increase in GEP (Figure 4a) or aboveground NPP (Liu, H., et al., 2018; Liu, Q., et al., 2018). This is in line with field observations. Unlike herbaceous communities that shift its C allocation toward belowground (Liu, H., et al., 2018; Liu, Q., et al., 2018), shrub communities having a higher use efficiency of water respond to warming often by an increase in abundance, canopy, and height (Myers‐Smith et al., 2015; Wookey et al., 2009). However, to identify how herbaceous and woody communities differentially respond to warming, more investigations are urgently required.

Plant functional groups could affect warming effects on permafrost soil N availability, as soil NH_4_^+^‐N increases only in herbaceous communities (Figure 4k; Table S5). However, evidence of shrub soil N availability negatively affected by warming is a lack (Figure 4k, l), despite the prediction that warming would increase the height and leaf area index of a shrub to reduce soil N availability in summer through shading effects (Wookey et al., 2009). Indeed, soil total N decreases often with warming at sites dominated by shrubs (Figure 4j). This may be owe to the fact that shrub communities with lignified litter are slow in N turnover (Wookey et al., 2009). Habitats with lignified litter naturally select for microbiomes that are efficient in mining N from recalcitrant organic matter to cope with N limitation, for example, fungi (Bragazza et al., 2013; Deslippe et al., 2012; Salazar et al., 2020), leading to a rapid depletion of N in soils.

In consistence with the third hypothesis, soil moisture status will strongly regulate warming effects on permafrost NEE, AGB, BGB, and green leaf N (Figure 4, Table S5). As revealed, it was in the dry, not moist and wet, site where AGB declined that NEE was often downregulated by warming (Figure 4a–4d). In agreement with previous reports (Liu, H., et al., 2018; Liu, Q., et al., 2018), our work also suggests that compared with C exchange (e.g., GEP and ER, Figure 4a,b), soil moisture status would have more of an effect on C allocation in cold biomes (e.g., AGB and BGB, Figure 4d,e). Green leaf N decline with warming usually occurs in dry and wet conditions. At dry sites where AGB is often reduced by warming, green leaf N declines as soil nutrients increase (e.g., NH_4_^+^‐N, Figure 4d,k). This might suggest a reduction in N uptake by vegetation. Green leaf N declined as AGB increased (Figure 4d,f), suggesting that dilution‐based mechanisms could operate in wet conditions (An et al., 2005; Gao & Yan, 2019).

Obviously, warming phase, level, and timing are important factors affecting permafrost C‐ and nutrient–climate relationships (Table S5). For example, unlike growing‐season warming, year‐round warming, which usually induces soil water a decline and no increase in AGB, is not to stimulate GEP (Figure 4a,d,i). This suggests a concern over the negative effects of nongrowing season warming on growing‐season soil water, C uptake, and biomass production (Natali et al., 2012; Soja et al., 2007). ER temperature sensitivity would be in proportion to warming levels (Figure 4b). Low warming levels, whose effects on BGB, not AGB, are positive and do not lead to N decline in green leaves (Figure 4d–f). Green leaf N declines generally under high warming levels whose effects on AGB, not BGB, are clearly positive (Figure 4d–f). Such an observation might suggest a greater effect from aboveground than belowground response on green leaf N (Figure 5). Alpine or arctic NEE is often downregulated by short‐term warming that is not usually to have a stimulation on GEP (Figure 4a,c). Compared with short‐term warming, green leaf N declines more under long‐term warming (Figure 4f). One possibility for this would be a greater loss of water under long‐term warming (Figure 4i). This is to weaken vegetation capacity to capture N from soils (Wu et al., 2012).

## 5 CONCLUSIONS

Our meta‐analysis provides a quantitative basis for evidence‐based predictions of permafrost C–climate and N–climate feedbacks, and its modifications by habitat‐specific conditions (e.g., plant functional groups and soil moisture status) and warming variables (e.g., warming phases, levels, and timing). Although there will be an increase in C fixation (e.g., GEP, AGB, and BGB), warming is likely to induce a downregulation in alpine or arctic NEE by stimulating a larger biological response to respiration than to uptake. However, increased respiration activity may be not a result of microbial growth, as microbes in cold biomes are not often to respond to warming by an increase in biomass. Findings from this meta‐analysis highlight a high potential for warming to induce a downregulation of NEE in alpine or arctic sites that are dominated by shrub communities and are water‐limited. Probably because a lack of a positive effect on GEP, NEE downregulation may occur typically under short‐term warming. Although warming patterns could affect vegetation productivity and biomass, their effects on permafrost ecosystem respiration and net C uptake will be insignificant. Increasing warming levels would lead to a stronger respiration response and could affect C allocation by shifting its effects on AGB from negative to positive. Our work calls for an attention paid to how alpine or arctic vegetation N status may change with warming and its effect on permafrost C–climate feedback. This is because as a positive regulator of photosynthetic C fixation, N concentrations in green leaves will exhibit a decline if alpine or arctic vegetation are exposed to a warmer climate, despite increased N availability in the soils. Green leaf N decline could occur in various soil moisture conditions (e.g., wet and dry) and will be in proportion to warming levels and time. In future, additional investigations are required on how habitat‐specific conditions interact with factors of warming to affect alpine or arctic climate sensitivity.

## CONFLICT OF INTEREST

The authors declare no competing financial interests.

## AUTHOR CONTRIBUTION

**Wenlong Gao:** Conceptualization (lead); Data curation (lead); Formal analysis (lead); Funding acquisition (lead); Resources (lead); Writing‐original draft (lead); Writing‐review & editing (lead). **Weimin Sun:** Writing‐review & editing (equal). **Xingliang Xu:** Conceptualization (equal); Formal analysis (equal); Writing‐review & editing (lead).

## Supporting information

Figure S1

Figure S2

Table S1

Table S2

Table S3

Table S4

Table S5

## Data Availability

Supplementary materials are deposited at Dryad Dataset, https://doi.org/10.5061/dryad.jdfn2z3c1
