# Supplementary figures and images for: Permafrost response to temperature rise in carbon and nutrient cycling: Effects from habitat‐specific conditions and factors of warming
Source: Ecol Evol. 2021 Oct 27;11(22):16021–33. doi: 10.1002/ece3.8271 (PMC8601908; doi:10.1002/ece3.8271)

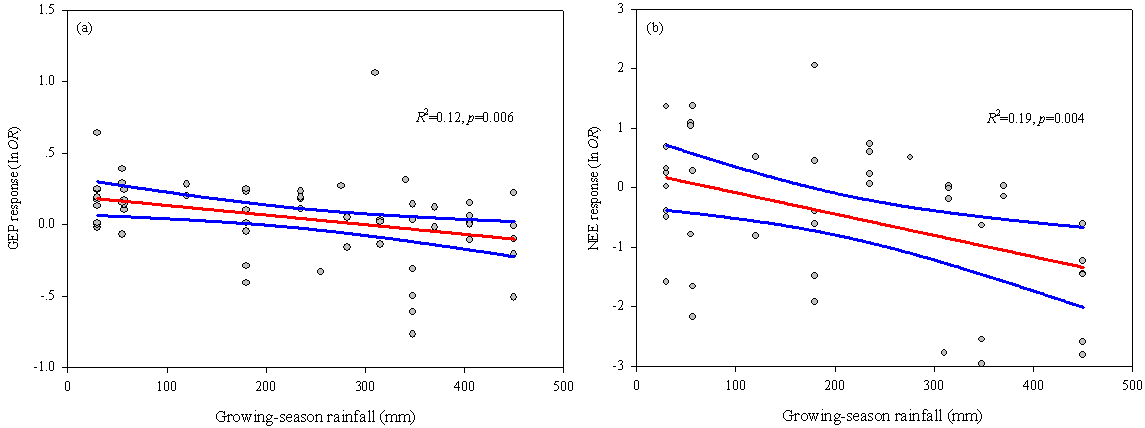

Supplement: Supplementary file 1 — Figure S1 [file ECE3-11-16021-s007.tif]

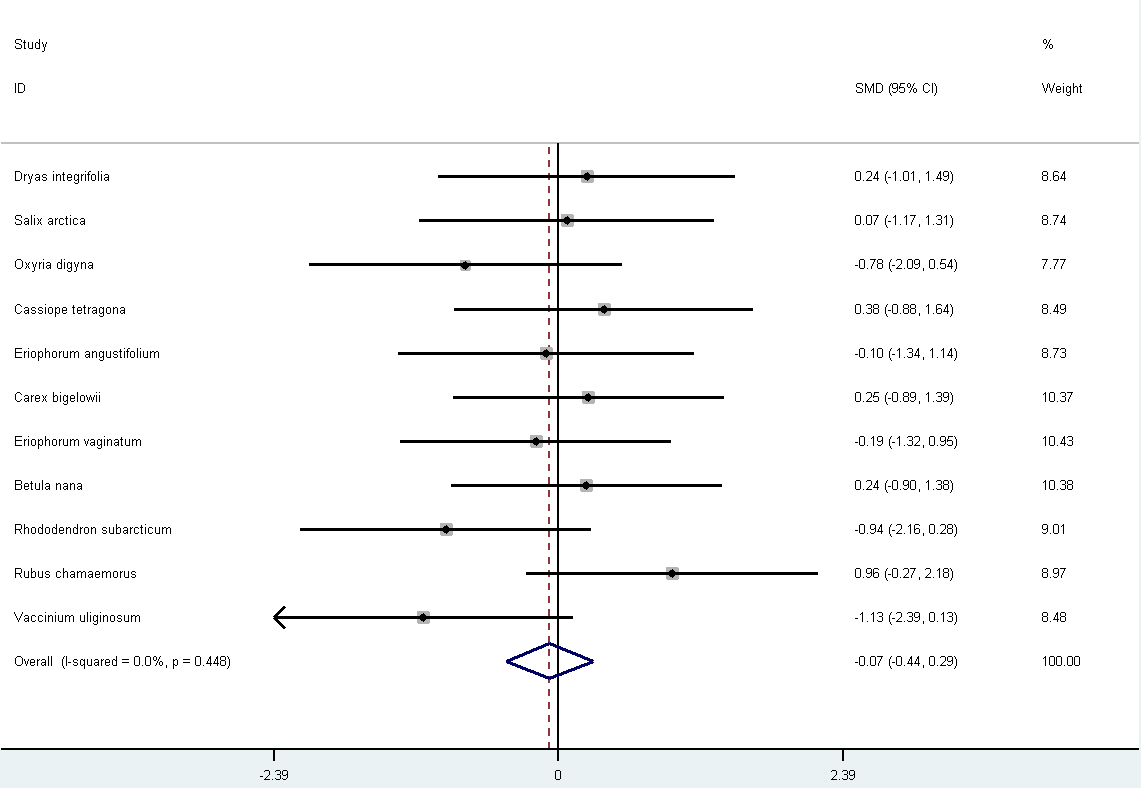

Supplement: Supplementary file 2 — Figure S2 [file ECE3-11-16021-s001.tif]
