# Supplementary material for: Permafrost response to temperature rise in carbon and nutrient cycling: Effects from habitat‐specific conditions and factors of warming
Source: Ecol Evol. 2021 Oct 27;11(22):16021–33. doi: 10.1002/ece3.8271 (PMC8601908; doi:10.1002/ece3.8271)
Supplement: Supplementary file 3 — Table S1 [file ECE3-11-16021-s002.docx]

**Table S1** Published meta-analyses on warming responses in permafrost C and nutrient cycling

| **Variable** | **Response** | **Significance** | **Sample** | **Data source** | **Reference** |
| --- | --- | --- | --- | --- | --- |
| Soil temperature | 21.70% | Yes | 10 | Tundra | Lu et al., (2013) |
| Soil temperature | 24.70% | Yes | 17 | Alpine meadow and steppe | Liu et al., (2018) |
| Soil moisture | -0.60% | No | 7 | Tundra | Lu et al., (2013) |
| Soil moisture | -3% | Yes | 16 | Alpine meadow and steppe | Liu et al., (2018) |
| GEP | 15.30% | Yes | 8 | Grasslands in cold biomes | Wang et al., (2019) |
| GEP | 2.83% | No | 29 | Alpine grasslands | Chen et al., (2020) |
| NPP | 23.60% | Yes | 12 | Grasslands in cold biomes | Wang et al., (2019) |
| NPP | 22.96% | No | 6 | Alpine grasslands | Chen et al., (2020) |
| ANPP | -1.50% | No | 34 | Alpine meadow and steppe | Liu et al., (2018) |
| ANPP | 20.50% | Yes | 23 | Grasslands in cold biomes | Wang et al., (2019) |
| BNPP | 19.00% | Yes | 12 | Grasslands in cold biomes | Wang et al., (2019) |
| ER | 8% | Yes | 18 | Tundra | Lu et al., (2013) |
| ER | 11% | Yes | 9 | Grasslands in cold biomes | Wang et al., (2019) |
| ER | 15% | Yes | 38 | Alpine grasslands | Chen et al., (2020) |
| Soil respiration | 11.30% | Yes | 12 | Tundra | Lu et al., (2013) |
| Soil respiration | 33.60% | Yes | 6 | Alpine forest and grassland | Zhang et al., (2015) |
| Soil respiration | 15.40% | Yes | 6 | Grasslands in cold biomes | Wang et al., (2019) |
| Soil respiration | 14.30% | Yes | 20 | Alpine grasslands | Chen et al., (2020) |
| NEE | -3.90% | No | 8 | Tundra | Lu et al., (2013) |
| NEE | 24.90% | Yes | 6 | Grasslands in cold biomes | Wang et al., (2019) |
| NEE | Hedges’d: -0.20 | No | 18 | Alpine grasslands | Chen et al., (2020) |
| Vegetation biomass | 19.00% | Yes |  | Alpine forest and grassland | Zhang et al., (2015) |
| AGB | 11.00% | Yes | 45 | Alpine grasslands | Chen et al., (2020) |
| BGB | 24.30% | Yes | 31 | Alpine grasslands | Chen et al., (2020) |
| SOC | 2.90% | No | 16 | Tundra | Lu et al., (2013) |
| SOC | -2.20% | No | 18 | Alpine forest and grassland | Zhang et al., (2015) |
| SOC | 2.40% | No | 41 | Alpine grasslands | Chen et al., (2020) |
| Microbial biomass | 14.30% | Yes | 26 | Alpine forest and grassland | Zhang et al., (2015) |
| Microbial biomass | 5.50% | No | 35 | Alpine grasslands | Chen et al., (2020) |
| Soil total N | -3.50% | No | 23 | Alpine forest and grassland | Zhang et al., (2015) |
| Soil total N | 1.90% | No | 45 | Alpine grasslands | Chen et al., (2020) |
| Soil NH_4_^+^ | 11.40% | No | 12 | Alpine forest and grassland | Zhang et al., (2015) |
| Soil NH_4_^+^ | 29.00% | Yes | 32 | Alpine grasslands | Chen et al., (2020) |
| Soil NO_3_^-^ | 39.20% | Yes | 12 | Alpine forest and grassland | Zhang et al., (2015) |
| Soil NO_3_^-^ | 10.90% | No | 33 | Alpine grasslands | Chen et al., (2020) |
| Net N mineralization | 49.20% | Yes | 7 | Alpine forest and grassland | Zhang et al., (2015) |
| Net nitrification | 56.00% | Yes | 4 | Alpine forest and grassland | Zhang et al., (2015) |

NPP: net primary productivity, ANPP: aboveground net primary productivity, BNPP: belowground net primary productivity.

**References**

Chen, Y., Feng, J., Yuan, X. and Zhu, B., 2020. Effects of warming on carbon and nitrogen cycling in alpine grassland ecosystems on the Tibetan Plateau: A meta-analysis. Geoderma, 370: 114363.

Liu, H., Mi, Z., Lin, L.I., Wang, Y., Zhang, Z., Zhang, F., Wang, H., Liu, L., Zhu, B. and Cao, G., 2018. Shifting plant species composition in response to climate change stabilizes grassland primary production. Proceedings of the National Academy of Sciences, 115(16): 4051-4056.

Lu, M., Zhou, X., Yang, Q., Li, H., Luo, Y., Fang, C., Chen, J., Yang, X. and Li, B., 2013. Responses of ecosystem carbon cycle to experimental warming: a meta‐analysis. Ecology, 94(3): 726-738.

Wang, N., Quesada, B., Xia, L., Butterbach‐Bahl, K., Goodale, C.L. and Kiese, R., 2019. Effects of climate warming on carbon fluxes in grasslands—A global meta‐analysis. Global change biology, 25(5): 1839-1851.

Zhang, X.-Z., Shen, Z.-X. and Fu, G., 2015. A meta-analysis of the effects of experimental warming on soil carbon and nitrogen dynamics on the Tibetan Plateau. Applied Soil Ecology, 87: 32-38.
