# Supplementary material for: Permafrost response to temperature rise in carbon and nutrient cycling: Effects from habitat‐specific conditions and factors of warming
Source: Ecol Evol. 2021 Oct 27;11(22):16021–33. doi: 10.1002/ece3.8271 (PMC8601908; doi:10.1002/ece3.8271)
Supplement: Supplementary file 4 — Table S2 [file ECE3-11-16021-s005.docx]

**Table S2** Data on soil temperature, soil mositure, ecosystem C flux, vegetation biomass, soil C, microbial biomass, and soil N

| **Response variable** | **Unit** | **Soil layer** | **Site** | **Ecosytem type** | **Growing-season rainfall** | **Growing-season temperature** | **Plant community** | **Soil water status** | **Warming pattern** | **Warming level** | | **Warming time** | | ***Xc*** | ***Sc*** | ***n_c_*** | ***Xt*** | ***S_t_*** | ***n_t_*** | **Citation** |
| --- | --- | --- | --- | --- | --- | --- | --- | --- | --- | --- | --- | --- | --- | --- | --- | --- | --- | --- | --- | --- |
|  |  | cm or layer |  |  | mm | ℃ |  |  |  | ℃ | Level | Growing-season | Time | Control mean | Standrad deviation | Sample size | Treatment mean | Standrad deviation | Sample size |  |
| Soil temperature | ℃ | 0-15 | Nagqu | Alpine meadow | 370 | 8.5 | Herb | Moist | Year-round | 0.8-1.1 | Low | 2 | Short | 12.66 | 1.39 | 120 | 13.76 | 0.93 | 120 | Wang et al. (2014) |
| Soil temperature | ℃ | 0-15 | Haibei | Alpine meadow | 370 | 8.5 | Herb | Moist | Year-round | 0.8-1.1 | Low | 10 | Long | 13.13 | 0.92 | 120 | 14.14 | 0.46 | 120 | Wang et al. (2014) |
| Soil temperature | ℃ | 10 | Haibei | Alpine meadow | 370 | 8.5 | Herb | Moist | Year-round | 1.2-1.7 | Low | 3 | Short | 8.86 | 2.48 | 5 | 10.92 | 2.20 | 5 | Hu etal. (2010) |
| Soil temperature | ℃ | 0-5 | Suli | Alpine meadow | 370 | 12.30 | Herb | Moist | Year-round | 2.00 | Low | 1 | Short | 9.70 | 1.25 | 48 | 9.08 | 1.04 | 48 | Yu et al. (2015) |
| Soil temperature | ℃ | 0-5 | Damxung | Alpine meadow | 370 | 10.7 | Herb | Moist | Year-round | 2.00 | Low | 4 | Long | 18.71 | 5.17 | 5 | 20.77 | 5.74 | 5 | Shen et al. (2015) |
| Soil temperature | ℃ | 0-5 | Kakagou | Alpine meadow | 574.4 | 7.33 | Herb | Moist | Year-round | >1.8 | High | 1 | Short | 12.82 | 0.76 | 3 | 13.78 | 0.87 | 3 | Shi et al. (2012) |
| Soil temperature | ℃ | 5 | Haibei | Alpine meadow | 370 | 8.5 | Herb | Moist | Growing-season | 1.2-1.7 | Low | 4 | Long | 10.21 | 0.30 | 4 | 13.04 | 0.15 | 4 | Rui et al. (2011) |
| Soil temperature | ℃ | 10 | Haibei | Alpine meadow | 370 | 8.5 | Herb | Moist | Year-round | 1.15 | Low | 5 | Long | 8.71 | 3.69 | 6 | 10.58 | 3.34 | 6 | Li etal. (2011) |
| Soil temperature | ℃ | 0-10 | Hongyuan | Alpine meadow | 600.00 | 6.60 | Herb | Moist | Year-round | 2.20 | High | 1 | Short | 11.28 | 0.79 | 4 | 12.45 | 0.87 | 4 | Wang et al. (2011) |
| Soil temperature | ℃ | 0-10 | Nam Tso | Alpine meadow | 330.00 | 6.80 | Herb | Moist | Growing-season | 1.30 | Low | 2 | Short | 9.83 | 0.36 | 8 | 11.79 | 0.74 | 8 | Dorji et al. (2013) |
| Soil temperature | ℃ | 0-10 | Nam Tso | Alpine meadow | 330.00 | 6.80 | Herb | Moist | Growing-season | 1.30 | Low | 3 | Short | 9.60 | 0.18 | 8 | 10.90 | 0.46 | 8 | Dorji et al. (2013) |
| Soil temperature | ℃ | 5 | Haibei | Alpine meadow | 370 | 8.5 | Herb | Moist | Growing-season | 1.2-1.7 | Low | 2 | Short | 6.46 | 0.25 | 4 | 8.45 | 1.25 | 4 | Luo et al. (2010) |
| Soil temperature | ℃ | 5 | Beiluhe | Alpine meadow | 276.00 | 6 | Herb | Moist | Year-round | 1.88 | Low | 1 | Short | 8.75 | 4.15 | 2 | 6.83 | 3.85 | 2 | Peng et al. (2014) |
| Soil temperature | ℃ | 5 | Beiluhe | Alpine meadow | 276.00 | 6 | Herb | Moist | Year-round | 3.99 | High | 1 | Short | 8.75 | 4.15 | 2 | 5.40 | 4.05 | 2 | Peng et al. (2014) |
| Soil temperature | ℃ | 5 | Beiluhe | Alpine meadow | 276.00 | 6 | Herb | Moist | Year-round | 1.88 | Low | 2 | Short | 10.59 | 2.84 | 4 | 8.91 | 2.97 | 4 | Peng et al. (2014) |
| Soil temperature | ℃ | 5 | Beiluhe | Alpine meadow | 276.00 | 6 | Herb | Moist | Year-round | 3.99 | High | 2 | Short | 10.59 | 2.84 | 4 | 7.59 | 3.46 | 4 | Peng et al. (2014) |
| Soil temperature | ℃ | 20 | Beiluhe | Alpine meadow | 256 | 6 | Herb | Moist | Year-round | 1.66 | Low | 2 | Short | 6.71 | 1.41 | 15 | 7.91 | 1.41 | 15 | Xu et al. (2014) |
| Soil temperature | ℃ | 20 | Beiluhe | Alpine meadow | 256 | 6 | Herb | Moist | Year-round | 2.18 | High | 2 | Short | 6.71 | 1.41 | 15 | 9.30 | 1.40 | 15 | Xu et al. (2014) |
| Soil temperature | ℃ | 5 | Damxung | Alpine meadow | 405 | 10.7 | Herb | Moist | Year-round | 1.00 | Low | 3 | Short | 17.99 | 6.88 | 12 | 20.13 | 8.82 | 12 | Geng et al. (2017) |
| Soil temperature | ℃ | 5 | Damxung | Alpine meadow | 405 | 10.7 | Herb | Moist | Year-round | 2.00 | Low | 3 | Short | 17.99 | 6.88 | 12 | 20.15 | 9.27 | 12 | Geng et al. (2017) |
| Soil temperature | ℃ | 5 | Damxung | Alpine meadow | 405 | 10.7 | Herb | Moist | Year-round | 3.00 | High | 3 | Short | 17.99 | 6.88 | 12 | 19.79 | 6.88 | 12 | Geng et al. (2017) |
| Soil temperature | ℃ | 5 | Damxung | Alpine meadow | 405 | 10.7 | Herb | Moist | Year-round | 4.00 | High | 3 | Short | 17.99 | 6.88 | 12 | 19.03 | 7.18 | 12 | Geng et al. (2017) |
| Soil temperature | ℃ | 0-10 | Beiluhe | Alpine meadow | 276.00 | 6 | Herb | Moist | Year-round | 1.88 | Low | 4 | Long | 0.67 | 0.73 | 5 | 2.21 | 0.73 | 5 | Peng et al. (2016) |
| Soil temperature | ℃ |  | Kakagou | Alpine meadow | 574.4 | 7.33 | Herb | Moist | Year-round | 1.40 | Low | 2 | Short | 12.05 | 3.36 | 5 | 12.00 | 3.55 | 5 | Shi et al. (2008) |
| Soil temperature | ℃ | 0-10 | Gangcha | Alpine steppe | 348 | 11.4 | Herb | Dry | Year-round | >2 | High | 3 | Short | 12.80 | 0.95 | 10 | 14.40 | 0.63 | 10 | Wang etal. (2018) |
| Soil temperature | ℃ | 0-10 | Gangcha | Alpine steppe | 348 | 11.4 | Herb | Dry | Year-round | >2 | High | 4 | Long | 13.90 | 0.63 | 10 | 15.40 | 0.95 | 10 | Wang etal. (2018) |
| Soil temperature | ℃ | 0-20 | Haiyan | Alpine meadow | 280 | 10.5 | Herb | Moist | Year-round | 2.00 | Low | 1 | Short | 10.81 | 4.17 | 3 | 12.22 | 4.54 | 3 | Zhao etal.(2017) |
| Soil temperature | ℃ | 0-20 | Gonghe | Alpine steppe | 302.00 | 11.10 | Herb | Dry | Year-round | 2.00 | Low | 1 | Short | 10.79 | 3.86 | 3 | 12.63 | 4.45 | 3 | Zhao etal.(2017) |
| Soil temperature | ℃ | 0-5 | Qilian Mountain | Alpine tundra | 330 | 12.38 | Herb | Moist | Year-round | 5.40 | High | 1 | Short | 12.83 | 3.30 | 9 | 15.96 | 3.53 | 9 | Zhang etal. (1996) |
| Soil temperature | ℃ |  | Toolik Lake | Arctic tundra | 180.00 | 10.00 | Herb+Shrub | Moist | Growing-season | 4-5 | High | 2 | Short | 2.90 | 1.12 | 5 | 4.70 | 1.57 | 5 | Hobbie etal. (1998) |
| Soil temperature | ℃ |  | Toolik Lake | Arctic tundra | 180.00 | 10.00 | Herb+Shrub | Moist | Growing-season | 4-5 | High | 3 | Short | 5.40 | 1.12 | 5 | 5.60 | 1.79 | 5 | Hobbie etal. (1998) |
| Soil temperature | ℃ |  | Toolik Lake | Arctic tundra | 180.00 | 10.00 | Herb+Shrub | Moist | Growing-season | 4-5 | High | 4 | Long | 7.60 | 1.34 | 5 | 8.80 | 1.34 | 5 | Hobbie etal. (1998) |
| Soil temperature | ℃ | 0-10 | Toolik Lake | Arctic tundra | 180.00 | 10.00 | Herb | Wet | Growing-season | 3.50 | High |  |  | 3.60 | 0.57 | 8 | 5.80 | 1.41 | 8 | Chapin III etal. (1995) |
| Soil temperature | ℃ | 10 | Toolik Lake | Arctic tundra | 180.00 | 10.00 | Shrub | Dry | Growing-season | 3.39 | High | 9 | Long | 5.02 | 0.23 | 2 | 5.78 | 0.23 | 2 | Demarco etal. (2014) |
| Soil temperature | ℃ | 0-10 | Abisko | Subarctic heath | 120.00 | 10.90 | Shrub | Wet | Growing-season | 2.80 | High | 5 | Long | 7.10 | 0.52 | 4 | 7.70 | 3.00 | 4 | Michelsen etal.(1996) |
| Soil temperature | ℃ | 0-10 | Abisko | Subarctic heath | 120.00 | 10.90 | Shrub | Wet | Growing-season | 2.80 | High | 5 | Long | 7.10 | 0.52 | 4 | 8.30 | 1.44 | 4 | Michelsen etal.(1996) |
| Soil temperature | ℃ | 0-10 | Abisko | Subarctic heath | 120.00 | 6.80 | Shrub | Dry | Growing-season | 2.80 | High | 5 | Long | 5.10 | 0.60 | 4 | 5.70 | 0.88 | 4 | Michelsen etal.(1996) |
| Soil temperature | ℃ | 0-10 | Abisko | Subarctic heath | 120.00 | 6.80 | Shrub | Dry | Growing-season | 2.80 | High | 5 | Long | 5.10 | 0.60 | 4 | 6.40 | 0.52 | 4 | Michelsen etal.(1996) |
| Soil temperature | ℃ | 0-10 | Abisko | Subarctic heath | 120.00 | 10.90 | Shrub | Wet | Growing-season | 2.80 | High | 6 | Long | 6.70 | 0.84 | 4 | 7.10 | 2.40 | 4 | Michelsen etal.(1996) |
| Soil temperature | ℃ | 0-10 | Abisko | Subarctic heath | 120.00 | 10.90 | Shrub | Wet | Growing-season | 2.80 | High | 6 | Long | 6.70 | 0.84 | 4 | 8.50 | 1.40 | 4 | Michelsen etal.(1996) |
| Soil temperature | ℃ | 0-10 | Abisko | Subarctic heath | 120.00 | 6.80 | Shrub | Dry | Growing-season | 2.80 | High | 6 | Long | 6.00 | 1.36 | 4 | 8.00 | 1.96 | 4 | Michelsen etal.(1996) |
| Soil temperature | ℃ | 0-10 | Abisko | Subarctic heath | 120.00 | 6.80 | Shrub | Dry | Growing-season | 2.80 | High | 6 | Long | 6.00 | 1.36 | 4 | 7.20 | 64.00 | 4 | Michelsen etal.(1996) |
| Soil temperature | ℃ | 8 | Abisko | Subarctic heath | 120.00 | 8.90 | Shrub | Wet | Growing-season | 3.90 | High |  |  | 6.47 | 1.31 | 4 | 7.18 | 1.12 | 4 | Christensen etal.(1997) |
| Soil temperature | ℃ | 0-2 | Abisko | Subarctic heath | 120.00 | 11.90 | Shrub | Wet | Growing-season | 1.00 | Low | 16 | Long | 12.53 | 0.99 | 6 | 12.93 | 0.98 | 6 | Pedersen etal.(2017) |
| Soil temperature | ℃ | 0-10 | Hongyuan | Alpine meadow | 600.00 | 6.60 | Herb | Moist | Year-round |  |  | 4-6 | Long | 10.48 | 4.12 | 6 | 10.90 | 4.94 | 6 | Zi etal. (2018) |
| Soil temperature | ℃ | 0-10 | Beiluhe | Alpine meadow | 276.00 | 6 | Herb | Moist | Year-round | >1 |  | 1-3 | Short | 0.31 | 0.56 | 4 | 2.06 | 0.54 | 4 | Peng etal. (2015) |
| Soil moisture | % | 10 | Beiluhe | Alpine meadow | 276 | 6 | Herb | Moist | Year-round | 2.30 | High | 2 | Short | 17.06 | 1.90 | 5 | 14.41 | 1.08 | 5 | Xue et al. (2015) |
| Soil moisture | % | 10 | Beiluhe | Alpine meadow | 276 | 6 | Herb | Moist | Year-round | 2.70 | High | 3 | Short | 17.03 | 1.33 | 5 | 15.47 | 1.07 | 5 | Xue et al. (2015) |
| Soil moisture | % | 0-15 | Nagqu | Alpine meadow | 370 | 8.5 | Herb | Moist | Year-round | 0.8-1.1 | Low | 2 | Short | 23.64 | 6.07 | 120 | 13.39 | 6.07 | 120 | Wang et al. (2014) |
| Soil moisture | % | 0-15 | Haibei | Alpine meadow | 370 | 8.5 | Herb | Moist | Year-round | 0.8-1.1 | Low | 10 | Long | 25.67 | 11.12 | 120 | 16.07 | 10.11 | 120 | Wang et al. (2014) |
| Soil moisture | % | 20 | Haibei | Alpine meadow | 370 | 8.5 | Herb | Moist | Year-round | 1.2-1.7 | Low | 3 | Short | 24.44 | 4.34 | 5 | 21.30 | 4.69 | 5 | Hu etal. (2010) |
| Soil moisture | % | 0-20 | Haibei | Alpine meadow | 370 | 8.5 | Herb | Moist | Year-round | 2.00 | Low | 1 | Short | 27.76 | 1.12 | 6 | 27.64 | 1.12 | 6 | Liu et al. (2018) |
| Soil moisture | % | 0-20 | Haibei | Alpine meadow | 370 | 8.5 | Herb | Moist | Year-round | 2.00 | Low | 2 | Short | 25.20 | 2.21 | 6 | 22.11 | 0.74 | 6 | Liu et al. (2018) |
| Soil moisture | % | 0-20 | Haibei | Alpine meadow | 370 | 8.5 | Herb | Moist | Year-round | 3.00 | High | 3 | Short | 26.66 | 2.22 | 6 | 22.52 | 1.12 | 6 | Liu et al. (2018) |
| Soil moisture | % | 0-20 | Haibei | Alpine meadow | 370 | 8.5 | Herb | Moist | Year-round | 3.00 | High | 4 | Long | 30.06 | 1.48 | 6 | 26.22 | 3.30 | 6 | Liu et al. (2018) |
| Soil moisture | % | 0-10 | Haibei | Alpine meadow | 370 | 8.5 | Herb | Moist | Growing-season | 1.2-1.7 | Low | 4 | Long | 35.40 | 13.40 | 4 | 26.30 | 5.40 | 4 | Zheng et al. (2012) |
| Soil moisture | % | 0-10 | Damxung | Alpine meadow | 405 | 10.7 | Herb | Moist | Year-round | 2.00 | Low | 4 | Long | 0.14 | 0.08 | 5 | 0.11 | 0.07 | 5 | Shen et al. (2015) |
| Soil moisture | % | 0-5 | Kakagou | Alpine meadow | 574.4 | 7.33 | Herb | Moist | Year-round | >1.8 | High | 1 | Short | 33.65 | 3.97 | 3 | 31.39 | 4.06 | 3 | Shi et al. (2012) |
| Soil moisture | % | 0–10 | Haibei | Alpine meadow | 370 | 8.5 | Herb | Moist | Growing-season | 1.2-1.7 | Low | 4 | Long | 42.76 | 6.02 | 4 | 27.66 | 3.87 | 4 | Rui et al. (2011) |
| Soil moisture | % | 0-10 | Hongyuan | Alpine meadow | 600.00 | 6.60 | Herb | Moist | Year-round | 2.2 | High | 1 | Short | 0.38 | 0.03 | 4 | 0.34 | 0.03 | 4 | Wang et al. (2011) |
| Soil moisture | % | 0–5 | Haibei | Alpine meadow | 370 | 8.5 | Herb | Moist | Year-round | 0.56 | Low | 4 | Long | 29.36 | 1.82 | 4 | 21.55 | 1.69 | 4 | Wu et al. (2016) |
| Soil moisture | % | 20 | Fenghuoshan | Alpine meadow | 216 | 2.5 | Herb | Moist | Year-round | 2.10 | High | 2 | Short | 39.49 | 10.51 | 5 | 36.83 | 8.91 | 5 | Li etal. (2011) |
| Soil moisture | % | 0-10 | Beiluhe | Alpine meadow | 276.00 | 6 | Herb | Moist | Year-round | 1.88 | Low | 2 | Short | 9.28 | 4.03 | 5 | 9.73 | 3.83 | 5 | Peng et al. (2014) |
| Soil moisture | % | 0-10 | Beiluhe | Alpine meadow | 276.00 | 6 | Herb | Moist | Year-round | 3.99 | High | 2 | Short | 9.28 | 4.03 | 5 | 11.98 | 4.43 | 5 | Peng et al. (2014) |
| Soil moisture | % | 10 | Beiluhe | Alpine meadow | 256 | 6 | Herb | Moist | Year-round | 3.00 | High | 2-4 |  | 8.80 | 23.12 | 289 | 8.60 | 21.25 | 289 | Xu et al. (2015) |
| Soil moisture | % | 20 | Beiluhe | Alpine meadow | 256 | 6 | Herb | Moist | Year-round | 1.66 | Low | 2 | Short | 7.39 | 4.28 | 15 | 6.38 | 4.85 | 15 | Xu et al. (2014) |
| Soil moisture | % | 20 | Beiluhe | Alpine meadow | 256 | 6 | Herb | Moist | Year-round | 2.18 | High | 2 | Short | 7.39 | 4.28 | 15 | 6.18 | 5.59 | 15 | Xu et al. (2014) |
| Soil moisture | % | 0-10 | Damxung | Alpine meadow | 405 | 10.7 | Herb | Moist | Year-round | 3.00 | High | 3 | Short | 0.24 | 0.07 | 12 | 0.22 | 0.07 | 12 | Geng et al. (2017) |
| Soil moisture | % | 0-10 | Damxung | Alpine meadow | 405 | 10.7 | Herb | Moist | Year-round | 4.00 | High | 3 | Short | 0.24 | 0.07 | 12 | 0.24 | 0.07 | 12 | Geng et al. (2017) |
| Soil moisture | % | 0-10 | Damxung | Alpine meadow | 405 | 10.7 | Herb | Moist | Year-round | 4.00 | High | 3 | Short | 0.24 | 0.07 | 12 | 0.21 | 0.09 | 12 | Geng et al. (2017) |
| Soil moisture | % | 0-10 | Damxung | Alpine meadow | 405 | 10.7 | Herb | Moist | Year-round | 4.00 | High | 3 | Short | 0.24 | 0.07 | 12 | 0.20 | 0.09 | 12 | Geng et al. (2017) |
| Soil moisture | % | 0-10 | Beiluhe | Alpine meadow | 276.00 | 6 | Herb | Moist | Year-round | 1.88 | Low | 4 | Long | 9.30 | 0.56 | 5 | 9.07 | 0.50 | 5 | Peng et al. (2016) |
| Soil moisture | % | 20 | Fenghuoshan | Alpine meadow | 216 | 2.5 | Herb | Moist | Year-round | 2.59 | High | 2 | Short | 46.53 | 11.86 | 5 | 44.16 | 10.98 | 5 | Li etal. (2011) |
| Soil moisture | % | 20 | Fenghuoshan | Alpine meadow | 216 | 2.5 | Herb | Moist | Year-round | 5.16 | High | 2 | Short | 46.53 | 11.86 | 5 | 38.43 | 10.64 | 5 | Li etal. (2011) |
| Soil moisture | % | 0-10 | Gangcha | Alpine steppe | 348 | 11.4 | Herb | Dry | Year-round | >2 | High | 3 | Short | 13.20 | 5.69 | 10 | 9.80 | 3.48 | 10 | Wang etal. (2018) |
| Soil moisture | % | 0-10 | Gangcha | Alpine steppe | 348 | 11.4 | Herb | Dry | Year-round | >2 | High | 4 | Long | 15.60 | 5.69 | 10 | 13.30 | 3.79 | 10 | Wang etal. (2018) |
| Soil moisture | % | 0-20 | Eight Mile Lake | Arctic tundra | 235 | 13.5 | Herb+Shrub | Moist | Growing-season | 1 | Low | 2 | Short | 5.60 | 1.71 | 6 | 7.40 | 1.96 | 6 | Natali etal. (2014) |
| Soil moisture | % | 0-20 | Eight Mile Lake | Arctic tundra | 235 | 13.5 | Herb+Shrub | Moist | Growing-season | 1 | Low | 3 | Short | 5.40 | 1.71 | 6 | 8.80 | 1.47 | 6 | Natali etal. (2014) |
| Soil moisture | % |  | Alexandra Fiord | Arctic tundra | 30 | 8.7 | Shrub | Dry | Year-round | 1-2 | Low | 17 | Long | 13.00 | 6.00 | 9 | 12.00 | 6.00 | 9 | Hudson etal. (2011) |
| Soil moisture | % |  | Alexandra Fiord | Arctic tundra | 30 | 7.9 | Shrub | Dry | Year-round | 1-2 | Low | 17 | Long | 18.00 | 6.71 | 5 | 18.00 | 6.71 | 5 | Hudson etal. (2011) |
| Soil moisture | % |  | Alexandra Fiord | Arctic tundra | 30 | 8.1 | Herb | Wet | Year-round | 1-2 | Low | 17 | Long | 49.00 | 4.47 | 5 | 44.00 | 4.47 | 5 | Hudson etal. (2011) |
| Soil moisture | % | 0–20 | Eight Mile Lake | Arctic tundra | 235 | 15 | Herb+Shrub | Moist | Growing-season | 0 | Low | 4 | Long | 42.50 | 5.88 | 6 | 42.90 | 4.41 | 6 | Deane-Coe etal. (2015) |
| Soil moisture | % | 0–20 | Eight Mile Lake | Arctic tundra | 235 | 15 | Herb+Shrub | Moist | Growing-season | 0.4 | Low | 5 | Long | 37.80 | 8.33 | 6 | 38.50 | 3.67 | 6 | Deane-Coe etal. (2015) |
| Soil moisture | % |  | Abisko | Subarctic heath | 120.00 | 11.90 | Moss | Wet | Growing-season | 3-4 | High | 10 | Long | 0.24 | 0.28 | 5 | 0.05 | 0.06 | 5 | Sorensen etal.(2011) |
| Soil moisture | % | 0-5 | Abisko | Subarctic heath | 120.00 | 11.90 | Shrub | Wet | Growing-season | 1 | Low | 16 | Long | 34.12 | 2.97 | 6 | 33.52 | 3.47 | 6 | Pedersen etal.(2017) |
| Soil moisture | % | 0-20 | Fenghuoshan | Alpine meadow | 216 | 2.5 | Herb | Moist | Year-round | 2.59 | High | 1-3 | Short | 46.48 | 12.28 | 5 | 44.71 | 10.68 | 5 | Peng etal.(2017) |
| Soil moisture | % | 0-20 | Fenghuoshan | Alpine swamp meadow | 216 | 1.42 | Herb | Wet | Year-round | 3.1 | High | 1-3 | Short | 51.94 | 1.97 | 5 | 49.31 | 1.75 | 5 | Peng etal.(2017) |
| Soil moisture | % | 0-10 | Damxung | Alpine meadow | 405 | 10.7 | Herb | Moist | Year-round | 2.95 | High | 1 | Short | 0.11 | 0.02 | 3 | 0.08 | 0.01 | 3 | Fu etal. (2019) |
| Soil moisture | % | 0-10 | Damxung | Alpine meadow | 405 | 10.7 | Herb | Moist | Year-round | 2.76 | High | 1 | Short | 0.11 | 0.02 | 3 | 0.06 | 0.01 | 3 | Fu etal. (2019) |
| Soil moisture | % | 0-10 | Damxung | Alpine meadow | 405 | 10.7 | Herb | Moist | Year-round | 2.95 | High | 2 | Short | 0.14 | 0.03 | 3 | 0.12 | 0.00 | 3 | Fu etal. (2019) |
| Soil moisture | % | 0-10 | Damxung | Alpine meadow | 405 | 10.7 | Herb | Moist | Year-round | 2.76 | High | 2 | Short | 0.14 | 0.03 | 3 | 0.09 | 0.04 | 3 | Fu etal. (2019) |
| Soil moisture | % | 0-10 | Damxung | Alpine meadow | 405 | 10.7 | Herb | Moist | Year-round | 2.95 | High | 3 | Short | 0.12 | 0.03 | 3 | 0.11 | 0.02 | 3 | Fu etal. (2019) |
| Soil moisture | % | 0-10 | Damxung | Alpine meadow | 405 | 10.7 | Herb | Moist | Year-round | 2.76 | High | 3 | Short | 0.12 | 0.03 | 3 | 0.08 | 0.01 | 3 | Fu etal. (2019) |
| Soil moisture | % | 0-10 | Beiluhe | Alpine meadow | 276.00 | 6 | Herb | Moist | Year-round | >1 |  | 1-3 | Short | 5.84 | 0.46 | 4 | 3.98 | 0.22 | 4 | Peng etal. (2015) |
| Soil moisture | % | 0-10 | Beiluhe | Alpine meadow | 276.00 | 6 | Herb | Moist | Year-round | >1 |  | 1-3 | Short | 12.36 | 0.63 | 5 | 10.21 | 0.64 | 5 | Peng etal. (2015) |
| Soil moisture | % | 0-10 | Beiluhe | Alpine swamp meadow | 306 | 6 | Herb | Wet | Year-round | >1.7 | High | 3 | Short | 39.30 | 0.88 | 3 | 37.20 | 1.04 | 3 | Zhang etal. (2014) |
| Soil moisture | % | 0-10 | Beiluhe | Alpine meadow | 306 | 6 | Herb | Moist | Year-round | >2.3 | High | 3 | Short | 37.80 | 1.02 | 3 | 35.80 | 1.39 | 3 | Zhang etal. (2014) |
| Soil moisture | % | 0-10 | Beiluhe | Alpine steppe | 306 | 6 | Herb+Shrub | Dry | Year-round | >1.7 | High | 3 | Short | 7.08 | 0.23 | 3 | 6.16 | 0.24 | 3 | Zhang etal. (2014) |
| AGB | g/m^2^ |  | Haibei | Alpine meadow | 370 | 8.5 | Herb | Moist | Year-round | 2.00 | Low | 1 | Short | 360.00 | 68.59 | 6 | 396.00 | 107.78 | 6 | Liu et al. (2018) |
| AGB | g/m^2^ |  | Haibei | Alpine meadow | 370 | 8.5 | Herb | Moist | Year-round | 2.00 | Low | 2 | Short | 300.00 | 97.98 | 6 | 216.00 | 68.59 | 6 | Liu et al. (2018) |
| AGB | g/m^2^ |  | Haibei | Alpine meadow | 370 | 8.5 | Herb | Moist | Year-round | 3.00 | High | 3 | Short | 344.00 | 39.19 | 6 | 332.00 | 48.99 | 6 | Liu et al. (2018) |
| AGB | g/m^2^ |  | Haibei | Alpine meadow | 370 | 8.5 | Herb | Moist | Year-round | 3.00 | High | 4 | Long | 488.00 | 117.58 | 6 | 448.00 | 88.18 | 6 | Liu et al. (2018) |
| AGB | g/m^2^ |  | Hongyuan | Alpine meadow | 216 | 6.60 | Herb | Moist | Year-round | 1.00 | Low | 1 | Short | 427.15 | 64.23 | 10 | 459.71 | 68.53 | 10 | Li etal. (2011) |
| AGB | g/m^2^ |  | Hongyuan | Alpine meadow | 216 | 6.60 | Herb | Moist | Year-round | 1.00 | Low | 2 | Short | 319.49 | 42.88 | 10 | 354.76 | 47.15 | 10 | Li etal. (2011) |
| AGB | g/m^2^ |  | Hongyuan | Alpine meadow | 216 | 6.60 | Herb | Moist | Year-round | 1.00 | Low | 3 | Short | 352.37 | 47.15 | 10 | 295.76 | 34.25 | 10 | Li etal. (2011) |
| AGB | g/m^2^ |  | Damxung | Alpine meadow | 405 | 10.7 | Herb | Moist | Year-round | 2.00 | Low | 3 | Short | 150.29 | 53.69 | 4 | 86.57 | 49.99 | 4 | Zong etal. (2013) |
| AGB | g/m^2^ |  | Fenghuoshan | Alpine meadow | 216 | 2.5 | Herb | Moist | Year-round | 2.00 | Low | 2 | Short | 297.29 | 119.27 | 5 | 383.81 | 126.67 | 5 | Li etal. (2011) |
| AGB | g/m^2^ |  | Fenghuoshan | Alpine meadow | 216 | 2.5 | Herb | Moist | Year-round | 4.00 | High | 2 | Short | 297.29 | 119.27 | 5 | 296.26 | 119.29 | 5 | Li etal. (2011) |
| AGB | g/m^2^ |  | Haibei | Alpine meadow | 370 | 8.5 | Herb | Moist | Year-round | 1.15 | Low | 5 | Long | 496.35 | 35.41 | 4 | 448.79 | 32.02 | 4 | Li etal. (2011) |
| AGB | g/m^2^ |  | Beiluhe | Alpine meadow | 256 | 6 | Herb | Moist | Year-round | 3.00 | High | 4 | Long | 245.64 | 75.60 | 40 | 257.21 | 70.15 | 40 | Xu et al. (2016) |
| AGB | g/m^2^ |  | Fenghuoshan | Alpine meadow | 216 | 2.5 | Herb | Moist | Year-round | 2.10 | High | 2 | Short | 201.87 | 103.59 | 3 | 261.68 | 116.55 | 3 | Li etal. (2011) |
| AGB | g/m^2^ |  | Great Basin Experimental Range | Alpine meadow | 171 | 14 | Herb | Moist | Year-round | 1-2 | Low | 1 | Short | 257.95 | 157.77 | 32 | 248.37 | 123.21 | 32 | Gill et al. (2014) |
| AGB | g/m^2^ |  | Great Basin Experimental Range | Alpine meadow | 171 | 14 | Herb | Moist | Year-round | 1-2 | Low | 2 | Short | 326.80 | 123.21 | 32 | 313.73 | 93.62 | 32 | Gill et al. (2014) |
| AGB | g/m^2^ |  | Great Basin Experimental Range | Alpine meadow | 171 | 14 | Herb | Moist | Year-round | 1-2 | Low | 3 | Short | 230.94 | 271.13 | 32 | 233.55 | 241.55 | 32 | Gill et al. (2014) |
| AGB | g/m^2^ |  | Damxung | Alpine meadow | 405 | 10.7 | Herb | Moist | Year-round | 1.08 | Low | 2 | Short | 31.47 | 11.27 | 7 | 26.20 | 6.82 | 7 | Fu et al. (2012) |
| AGB | g/m^2^ |  | Damxung | Alpine meadow | 405 | 10.7 | Herb | Moist | Year-round | 1.81 | Low | 2 | Short | 39.69 | 16.75 | 7 | 35.65 | 13.45 | 7 | Fu et al. (2012) |
| AGB | g/m^2^ |  | Damxung | Alpine meadow | 405 | 10.7 | Herb | Moist | Year-round | 0.94 | Low | 2 | Short | 48.53 | 19.84 | 7 | 47.78 | 24.66 | 7 | Fu et al. (2012) |
| AGB | g/m^2^ |  | Haibei | Alpine meadow | 370 | 8.5 | Herb | Moist | Year-round | 1.2-1.7 | Low | 1 | Short | 286.94 | 15.40 | 4 | 340.70 | 15.42 | 4 | Lin et al. (2011) |
| AGB | g/m^2^ |  | Haibei | Alpine meadow | 370 | 8.5 | Herb | Moist | Year-round | 1.2-1.7 | Low | 2 | Short | 210.44 | 82.58 | 7 | 272.86 | 111.09 | 7 | Lin et al. (2011) |
| AGB | g/m^2^ |  | Haibei | Alpine meadow | 370 | 8.5 | Herb | Moist | Year-round | 1.2-1.7 | Low | 3 | Short | 211.95 | 73.71 | 8 | 305.16 | 124.44 | 8 | Lin et al. (2011) |
| AGB | g/m^2^ |  | Beiluhe | Alpine meadow | 256 | 6 | Herb | Moist | Year-round | 1.66 | Low | 2 | Short | 149.35 | 56.09 | 15 | 185.11 | 64.85 | 15 | Xu et al. (2014) |
| AGB | g/m^2^ |  | Beiluhe | Alpine meadow | 256 | 6 | Herb | Moist | Year-round | 2.18 | High | 2 | Short | 149.35 | 56.09 | 15 | 184.66 | 80.63 | 15 | Xu et al. (2014) |
| AGB | g/m^2^ |  | Sanjiangyuan | Alpine meadow | 421 | 7.6 | Herb | Moist | Year-round |  |  |  |  | 241.31 | 60.19 | 3 | 326.25 | 110.35 | 3 | Guo et al. (2015) |
| AGB | g/m^2^ |  | Haibei | Alpine meadow | 370 | 8.5 | Herb | Moist | Year-round | 1.0-2.0 | Low | 16 | Long | 740.44 | 47.40 | 8 | 571.12 | 90.22 | 8 | Zhao et al. (2016) |
| AGB | g/m^2^ |  | Beiluhe | Alpine meadow | 276.00 | 6 | Herb | Moist | Year-round | 1.88 | Low | 3 | Short | 422.12 | 102.09 | 5 | 416.00 | 105.26 | 5 | Peng et al. (2016) |
| AGB | g/m^2^ |  | Beiluhe | Alpine meadow | 276.00 | 6 | Herb | Moist | Year-round | 1.88 | Low | 4 | Long | 412.43 | 79.15 | 5 | 438.17 | 83.23 | 5 | Peng et al. (2016) |
| AGB | g/m^2^ |  | Kakagou | Alpine meadow | 574.4 | 7.33 | Herb | Moist | Year-round | 1.40 | Low | 2 | Short | 272.25 | 164.17 | 5 | 264.53 | 120.14 | 5 | Shi et al. (2008) |
| AGB | g/m^2^ |  | Ma Qin | Alpine meadow | 437 | 11.7 | Herb | Moist | Growing-season | 0.50 | Low | 1 | Short | 155.03 | 36.52 | 6 | 142.75 | 70.42 | 6 | Liu et al. (2010) |
| AGB | g/m^2^ |  | Ma Qin | Alpine meadow | 437 | 11.7 | Herb | Moist | Growing-season | 0.50 | Low | 2 | Short | 197.51 | 62.02 | 6 | 245.16 | 124.07 | 6 | Liu et al. (2010) |
| AGB | g/m^2^ |  | Ma Qin | Alpine meadow | 437 | 11.7 | Herb | Moist | Growing-season | 0.50 | Low | 3 | Short | 198.57 | 157.85 | 6 | 294.59 | 109.88 | 6 | Liu et al. (2010) |
| AGB | g/m^2^ |  | Ma Qin | Alpine meadow | 437 | 11.7 | Herb | Moist | Growing-season | 0.50 | Low | 4 | Long | 183.48 | 76.06 | 6 | 331.34 | 115.54 | 6 | Liu et al. (2010) |
| AGB | g/m^2^ |  | Ma Qin | Alpine meadow | 437 | 11.7 | Herb | Moist | Growing-season | 0.50 | Low | 5 | Long | 257.09 | 140.92 | 6 | 413.00 | 121.23 | 6 | Liu et al. (2010) |
| AGB | g/m^2^ |  | Ma Qin | Alpine meadow | 437 | 11.7 | Herb | Moist | Growing-season | 0.70 | Low | 1 | Short | 155.03 | 36.52 | 6 | 203.06 | 59.06 | 6 | Liu et al. (2010) |
| AGB | g/m^2^ |  | Ma Qin | Alpine meadow | 437 | 11.7 | Herb | Moist | Growing-season | 0.70 | Low | 2 | Short | 197.51 | 62.02 | 6 | 224.80 | 90.19 | 6 | Liu et al. (2010) |
| AGB | g/m^2^ |  | Ma Qin | Alpine meadow | 437 | 11.7 | Herb | Moist | Growing-season | 0.70 | Low | 3 | Short | 198.57 | 157.85 | 6 | 245.44 | 64.74 | 6 | Liu et al. (2010) |
| AGB | g/m^2^ |  | Ma Qin | Alpine meadow | 437 | 11.7 | Herb | Moist | Growing-season | 0.70 | Low | 4 | Long | 183.48 | 76.06 | 6 | 260.28 | 70.45 | 6 | Liu et al. (2010) |
| AGB | g/m^2^ |  | Ma Qin | Alpine meadow | 437 | 11.7 | Herb | Moist | Growing-season | 0.70 | Low | 5 | Long | 257.09 | 140.92 | 6 | 335.03 | 33.73 | 6 | Liu et al. (2010) |
| AGB | g/m^2^ |  | Ma Qin | Alpine meadow | 437 | 11.7 | Herb | Moist | Growing-season | 1.10 | Low | 1 | Short | 155.03 | 36.52 | 6 | 193.05 | 84.56 | 6 | Liu et al. (2010) |
| AGB | g/m^2^ |  | Ma Qin | Alpine meadow | 437 | 11.7 | Herb | Moist | Growing-season | 1.10 | Low | 2 | Short | 197.51 | 62.02 | 6 | 202.13 | 84.58 | 6 | Liu et al. (2010) |
| AGB | g/m^2^ |  | Ma Qin | Alpine meadow | 437 | 11.7 | Herb | Moist | Growing-season | 1.10 | Low | 3 | Short | 198.57 | 157.85 | 6 | 236.61 | 61.92 | 6 | Liu et al. (2010) |
| AGB | g/m^2^ |  | Ma Qin | Alpine meadow | 437 | 11.7 | Herb | Moist | Growing-season | 1.10 | Low | 4 | Long | 183.48 | 76.06 | 6 | 234.18 | 177.69 | 6 | Liu et al. (2010) |
| AGB | g/m^2^ |  | Ma Qin | Alpine meadow | 437 | 11.7 | Herb | Moist | Growing-season | 1.10 | Low | 5 | Long | 257.09 | 140.92 | 6 | 328.52 | 152.24 | 6 | Liu et al. (2010) |
| AGB | g/m^2^ |  | Ma Qin | Alpine meadow | 437 | 11.7 | Herb | Moist | Growing-season | 1.40 | Low | 1 | Short | 155.03 | 36.52 | 6 | 176.16 | 81.67 | 6 | Liu et al. (2010) |
| AGB | g/m^2^ |  | Ma Qin | Alpine meadow | 437 | 11.7 | Herb | Moist | Growing-season | 1.40 | Low | 2 | Short | 197.51 | 62.02 | 6 | 190.99 | 62.00 | 6 | Liu et al. (2010) |
| AGB | g/m^2^ |  | Ma Qin | Alpine meadow | 437 | 11.7 | Herb | Moist | Growing-season | 1.40 | Low | 3 | Short | 198.57 | 157.85 | 6 | 233.54 | 135.29 | 6 | Liu et al. (2010) |
| AGB | g/m^2^ |  | Ma Qin | Alpine meadow | 437 | 11.7 | Herb | Moist | Growing-season | 1.40 | Low | 4 | Long | 183.48 | 76.06 | 6 | 226.51 | 101.41 | 6 | Liu et al. (2010) |
| AGB | g/m^2^ |  | Ma Qin | Alpine meadow | 437 | 11.7 | Herb | Moist | Growing-season | 1.40 | Low | 5 | Long | 257.09 | 140.92 | 6 | 278.20 | 50.66 | 6 | Liu et al. (2010) |
| AGB | g/m^2^ |  | Ma Qin | Alpine meadow | 437 | 11.7 | Herb | Moist | Growing-season | 2.40 | High | 1 | Short | 155.03 | 36.52 | 6 | 165.01 | 50.61 | 6 | Liu et al. (2010) |
| AGB | g/m^2^ |  | Ma Qin | Alpine meadow | 437 | 11.7 | Herb | Moist | Growing-season | 2.40 | High | 2 | Short | 197.51 | 62.02 | 6 | 189.10 | 50.58 | 6 | Liu et al. (2010) |
| AGB | g/m^2^ |  | Ma Qin | Alpine meadow | 437 | 11.7 | Herb | Moist | Growing-season | 2.40 | High | 3 | Short | 198.57 | 157.85 | 6 | 198.21 | 109.96 | 6 | Liu et al. (2010) |
| AGB | g/m^2^ |  | Ma Qin | Alpine meadow | 437 | 11.7 | Herb | Moist | Growing-season | 2.40 | High | 4 | Long | 183.48 | 76.06 | 6 | 204.99 | 155.05 | 6 | Liu et al. (2010) |
| AGB | g/m^2^ |  | Ma Qin | Alpine meadow | 437 | 11.7 | Herb | Moist | Growing-season | 2.40 | High | 5 | Long | 257.09 | 140.92 | 6 | 267.07 | 95.80 | 6 | Liu et al. (2010) |
| AGB | g/m^2^ |  | Nagqu | Alpine meadow | 370 | 8.5 | Herb | Moist | Year-round | 1.10 | Low | 4 | Long | 171.02 | 14.85 | 3 | 193.15 | 36.34 | 3 | Jiang et al. (2017) |
| AGB | g/m^2^ |  | Nagqu | Alpine meadow | 370 | 8.5 | Herb | Moist | Year-round | 1.70 | Low | 4 | Long | 171.02 | 14.85 | 3 | 229.34 | 24.76 | 3 | Jiang et al. (2017) |
| AGB | g/m^2^ |  | Nagqu | Alpine meadow | 370 | 8.5 | Herb | Moist | Year-round | 2.10 | High | 4 | Long | 171.02 | 14.85 | 3 | 200.25 | 29.72 | 3 | Jiang et al. (2017) |
| AGB | g/m^2^ |  | Nagqu | Alpine meadow | 370 | 8.5 | Herb | Moist | Year-round | 2.70 | High | 4 | Long | 171.02 | 14.85 | 3 | 170.30 | 20.64 | 3 | Jiang et al. (2017) |
| AGB | g/m^2^ |  | Nagqu | Alpine meadow | 370 | 8.5 | Herb | Moist | Year-round | 2.70 | High | 2 | Short | 53.00 | 2.64 | 4 | 42.10 | 3.46 | 4 | Wang et al. (2018) |
| AGB | g/m^2^ |  | Nagqu | Alpine meadow | 370 | 8.5 | Herb | Moist | Year-round | 2.70 | High | 6 | Long | 53.00 | 2.64 | 4 | 63.85 | 2.64 | 4 | Wang et al. (2018) |
| AGB | g/m^2^ |  | Nagqu | Alpine meadow | 370 | 8.5 | Herb | Moist | Year-round | 1.70 | Low | 4 | Long | 61.82 | 14.25 | 3 | 80.74 | 21.37 | 3 | Zhang etal. (2015) |
| AGB | g/m^2^ |  | Fenghuoshan | Alpine meadow | 216 | 2.5 | Herb | Moist | Year-round | 2.59 | High | 2 | Short | 270.20 | 22.52 | 3 | 335.33 | 57.20 | 3 | Li etal. (2011) |
| AGB | g/m^2^ |  | Fenghuoshan | Alpine meadow | 216 | 2.5 | Herb | Moist | Year-round | 5.16 | High | 2 | Short | 270.20 | 22.52 | 3 | 279.97 | 28.09 | 3 | Li etal. (2011) |
| AGB | g/m^2^ |  | Gonghe | Alpine steppe | 348 | 11.4 | Herb | Dry | Year-round | >1 |  | 2 | Short | 127.54 | 8.16 | 10 | 135.29 | 13.60 | 10 | Li etal.(2019) |
| AGB | g/m^2^ |  | Gonghe | Alpine steppe | 348 | 11.4 | Herb | Dry | Year-round | >1 |  | 3 | Short | 133.26 | 10.78 | 10 | 136.64 | 10.72 | 10 | Li etal.(2019) |
| AGB | g/m^2^ |  | Nam Co | Alpine steppe | 332 | 12 | Herb | Dry | Growing-season | 2.00 | Low | 1 | Short | 97.01 | 29.86 | 4 | 87.06 | 22.40 | 4 | Zhao etal.(2019) |
| AGB | g/m^2^ |  | Nam Co | Alpine steppe | 332 | 12 | Herb | Dry | Growing-season | 2.00 | Low | 2 | Short | 123.13 | 17.42 | 4 | 100.75 | 29.84 | 4 | Zhao etal.(2019) |
| AGB | g/m^2^ |  | Nagqu | Alpine steppe | 348 | 11.4 | Herb | Dry | Year-round | 1.7-2.0 | Low | 2 | Short | 71.61 | 6.68 | 4 | 67.40 | 6.67 | 4 | Li etal.(2019) |
| AGB | g/m^2^ |  | Nagqu | Alpine steppe | 348 | 11.4 | Herb | Dry | Year-round | 1.7-2.0 | Low | 3 | Short | 73.70 | 7.61 | 4 | 58.58 | 6.67 | 4 | Li etal.(2019) |
| AGB | g/m^2^ |  | Nagqu | Alpine steppe | 348 | 11.4 | Herb | Dry | Year-round | 1.7-2.0 | Low | 4 | Long | 66.30 | 5.73 | 4 | 53.08 | 1.94 | 4 | Li etal.(2019) |
| AGB | g/m^2^ |  | Eight Mile Lake | Arctic tundra | 235 | 13.5 | Herb+Shrub | Moist | Growing-season | 1.00 | Low | 1 | Short | 518.30 | 50.46 | 6 | 538.90 | 68.34 | 6 | Natali etal. (2014) |
| AGB | g/m^2^ |  | Eight Mile Lake | Arctic tundra | 235 | 13.5 | Herb+Shrub | Moist | Growing-season | 1.00 | Low | 2 | Short | 552.50 | 47.77 | 6 | 557.90 | 48.25 | 6 | Natali etal. (2014) |
| AGB | g/m^2^ |  | Eight Mile Lake | Arctic tundra | 235 | 13.5 | Herb+Shrub | Moist | Growing-season | 1.00 | Low | 3 | Short | 557.60 | 107.04 | 6 | 578.80 | 75.93 | 6 | Natali etal. (2014) |
| AGB | g/m^2^ |  | Qilian Mountain | Alpine tundra | 330 | 12.38 | Herb | Moist | Year-round | 5.40 | High | 1 | Short | 266.89 | 78.89 | 4 | 272.67 | 87.39 | 4 | Zhang etal. (1996) |
| AGB | g/m^2^ |  | Barrow | Arctic tundra | 57 | 3.7 | Herb+Shrub | Moist | Growing-season | 1-2 | Low | 3-4 | Long | 631.40 | 242.80 | 12 | 587.60 | 276.10 | 12 | Hollister etal. (2010) |
| AGB | g/m^2^ |  | Daring Lake | Arctic tundra | 138 | 5.9 | Shrub | Moist | Growing-season | >2 | High | 8 | Long | 387.32 | 51.6979 | 5 | 513.16 | 83.1817 | 5 | Zamin etal. (2014) |
| AGB | g/m^2^ |  | Toolik Lake | Arctic tundra | 180.00 | 10.00 | Herb | Wet | Growing-season | 3.50 | High | 3 | Short | 1150.19 | 185.15 | 8 | 1064.10 | 235.86 | 8 | Chapin III etal. (1995) |
| AGB | g/m^2^ |  | Toolik Lake | Arctic tundra | 180.00 | 10.00 | Herb | Wet | Growing-season | 3.50 | High | 8 | Long | 1019.62 | 200.51 | 8 | 954.67 | 228.34 | 8 | Chapin III etal. (1995) |
| AGB | g/m^2^ |  | Toolik Lake | Arctic tundra | 180 | 10.00 | Herb | Wet | Growing-season |  |  | 14 | Long | 132.92 | 20.37 | 5 | 164.42 | 11.05 | 5 | Boelman etal. (2003) |
| AGB | g/m^2^ |  | Toolik Lake | Arctic tundra | 180.00 | 10.00 | Herb+Shrub | Moist | Growing-season | >2 | High | 20 | Long | 474.90 | 65.20 | 4 | 748.70 | 151.80 | 4 | Sistla etal. (2013) |
| AGB | g/m^2^ |  | Abisko | Subarctic heath | 180.00 | 10.00 | Herb+Shrub | Moist | Growing-season | 2-4 | High | 1 | Short | 666.60 | 121.80 | 4 | 700.80 | 71.00 | 4 | Richardson etal.(2002) |
| AGB | g/m^2^ |  | Abisko | Subarctic heath | 180.00 | 10.00 | Herb+Shrub | Moist | Growing-season | 2-4 | High | 9 | Long | 785.90 | 274.60 | 4 | 1095.00 | 213.40 | 4 | Richardson etal.(2002) |
| AGB | g/m^2^ |  | Fenghuoshan | Alpine swamp meadow | 216 | 1.42 | Herb | Wet | Year-round | 6.20 | High | 4 | Long | 553.30 | 109.40 | 4 | 946.80 | 182.40 | 4 | Chen etal.(2017) |
| AGB | g/m^2^ |  | Fenghuoshan | Alpine swamp meadow | 216 | 1.42 | Herb | Wet | Year-round | 1.5-2.5 |  | 3 | Short | 1070.40 | 128.55 | 2 | 1325.50 | 49.78 | 2 | Xi etal.(2019) |
| AGB | g/m^2^ |  | Fenghuoshan | Alpine swamp meadow | 216 | 1.42 | Herb | Wet | Year-round | 3-5 | High | 3 | Short | 1070.40 | 128.55 | 2 | 1624.85 | 500.14 | 2 | Xi etal.(2019) |
| AGB | g/m^2^ |  | Fenghuoshan | Alpine swamp meadow | 216 | 1.42 | Herb | Wet | Year-round | 2.98 | High | 2 | Short | 744.00 | 281.38 | 3 | 1074.33 | 495.17 | 3 | Li etal. (2011) |
| AGB | g/m^2^ |  | Fenghuoshan | Alpine swamp meadow | 216 | 1.42 | Herb | Wet | Year-round | 5.52 | High | 2 | Short | 744.00 | 281.38 | 3 | 989.00 | 633.43 | 3 | Li etal. (2011) |
| AGB | g/m^2^ |  | Damxung | Alpine meadow | 405 | 10.7 | Herb | Moist | Year-round | 1.91 | Low | 1 | Short | 18.50 | 2.23 | 4 | 19.73 | 2.64 | 4 | Fu etal. (2017) |
| AGB | g/m^2^ |  | Damxung | Alpine meadow | 405 | 10.7 | Herb | Moist | Year-round | 3.51 | High | 1 | Short | 18.50 | 2.23 | 4 | 21.54 | 3.30 | 4 | Fu etal. (2017) |
| AGB | g/m^2^ |  | Damxung | Alpine meadow | 405 | 10.7 | Herb | Moist | Year-round | 1.91 | Low | 2 | Short | 16.66 | 3.83 | 4 | 17.46 | 3.51 | 4 | Fu etal. (2017) |
| AGB | g/m^2^ |  | Damxung | Alpine meadow | 405 | 10.7 | Herb | Moist | Year-round | 3.51 | High | 2 | Short | 16.66 | 3.83 | 4 | 17.58 | 3.41 | 4 | Fu etal. (2017) |
| AGB | g/m^2^ |  | Damxung | Alpine meadow | 405 | 10.7 | Herb | Moist | Year-round | 1.91 | Low | 3 | Short | 17.84 | 3.50 | 4 | 17.94 | 4.04 | 4 | Fu etal. (2017) |
| AGB | g/m^2^ |  | Damxung | Alpine meadow | 405 | 10.7 | Herb | Moist | Year-round | 3.51 | High | 3 | Short | 17.84 | 3.50 | 4 | 18.87 | 4.57 | 4 | Fu etal. (2017) |
| AGB | g/m^2^ |  | Damxung | Alpine meadow | 405 | 10.7 | Herb | Moist | Year-round | 2.95 | High | 1 | Short | 16.92 | 4.10 | 4 | 16.51 | 3.15 | 4 | Fu etal. (2019) |
| AGB | g/m^2^ |  | Damxung | Alpine meadow | 405 | 10.7 | Herb | Moist | Year-round | 2.76 | High | 1 | Short | 16.92 | 4.10 | 4 | 14.29 | 1.77 | 4 | Fu etal. (2019) |
| AGB | g/m^2^ |  | Damxung | Alpine meadow | 405 | 10.7 | Herb | Moist | Year-round | 2.95 | High | 2 | Short | 18.14 | 3.56 | 4 | 20.92 | 4.07 | 4 | Fu etal. (2019) |
| AGB | g/m^2^ |  | Damxung | Alpine meadow | 405 | 10.7 | Herb | Moist | Year-round | 2.76 | High | 2 | Short | 18.14 | 3.56 | 4 | 18.20 | 4.57 | 4 | Fu etal. (2019) |
| AGB | g/m^2^ |  | Damxung | Alpine meadow | 405 | 10.7 | Herb | Moist | Year-round | 2.95 | High | 3 | Short | 15.50 | 2.16 | 4 | 18.62 | 3.57 | 4 | Fu etal. (2019) |
| AGB | g/m^2^ |  | Damxung | Alpine meadow | 405 | 10.7 | Herb | Moist | Year-round | 2.76 | High | 3 | Short | 15.50 | 2.16 | 4 | 15.72 | 1.81 | 4 | Fu etal. (2019) |
| AGB | g/m^2^ |  | Beiluhe | Alpine meadow | 276.00 | 6 | Herb | Moist | Year-round | >1 |  | 3 | Short | 388.12 | 75.22 | 4 | 415.20 | 62.28 | 4 | Peng etal. (2015) |
| AGB | g/m^2^ |  | Beiluhe | Alpine meadow | 276.00 | 6 | Herb | Moist | Year-round | >1 |  | 3 | Short | 379.68 | 81.19 | 5 | 353.55 | 86.98 | 5 | Peng etal. (2015) |
| AGB | g/m^2^ |  | Damxung | Alpine meadow | 405 | 10.7 | Herb | Moist | Year-round | 1.60 | Low | 3 | Short | 195.80 | 46.66 | 4 | 120.99 | 20.32 | 4 | Zong etal. (2018) |
| AGB | g/m^2^ |  | Damxung | Alpine meadow | 405 | 10.7 | Herb | Moist | Year-round | 1.60 | Low | 4 | Long | 124.72 | 29.10 | 4 | 76.28 | 17.48 | 4 | Zong etal. (2018) |
| AGB | g/m^2^ |  | Damxung | Alpine meadow | 405 | 10.7 | Herb | Moist | Year-round | 1.60 | Low | 5 | Long | 164.82 | 40.92 | 4 | 90.02 | 20.38 | 4 | Zong etal. (2018) |
| AGB | g/m^2^ |  | Damxung | Alpine meadow | 405 | 10.7 | Herb | Moist | Year-round | 1.60 | Low | 3 | Short | 190.32 | 69.52 | 5 | 120.53 | 25.00 | 5 | Zong etal. (2018) |
| AGB | g/m^2^ |  | Damxung | Alpine meadow | 405 | 10.7 | Herb | Moist | Year-round | 1.60 | Low | 4 | Long | 126.01 | 24.93 | 5 | 58.71 | 8.27 | 5 | Zong etal. (2018) |
| AGB | g/m^2^ |  | Damxung | Alpine meadow | 405 | 10.7 | Herb | Moist | Year-round | 1.60 | Low | 5 | Long | 166.26 | 36.11 | 5 | 92.73 | 16.61 | 5 | Zong etal. (2018) |
| AGB | g/m^2^ |  | Haibei | Alpine meadow | 370 | 8.5 | Herb | Moist | Year-round | >1 |  | 2 | Short | 296.23 | 21.51 | 6 | 327.06 | 10.73 | 6 | Chen etal.(2016) |
| AGB | g/m^2^ |  | Haibei | Alpine meadow | 370 | 8.5 | Herb | Moist | Year-round | >1 |  | 3 | Short | 335.34 | 26.94 | 6 | 403.78 | 21.46 | 6 | Chen etal.(2016) |
| AGB | g/m^2^ |  | Haibei | Alpine meadow | 370 | 8.5 | Herb | Moist | Year-round | >1 |  | 4 | Long | 325.74 | 16.09 | 6 | 385.29 | 32.38 | 6 | Chen etal.(2016) |
| AGB | g/m^2^ |  | Suli | Alpine meadow | 370 | 12.30 | Herb | Moist | Growing-season | 2.18 | High | 1 | Short | 202.19 | 50.35 | 3 | 284.32 | 65.38 | 3 | Yu et al. (2015) |
| AGB | g/m^2^ |  | Damxung | Alpine meadow | 405 | 10.7 | Herb | Moist | Year-round | 1-1.4 | Low | 3 | Short | 50.44 | 5.27 | 3 | 30.00 | 3.01 | 3 | Fu etal.(2013) |
| AGB | g/m^2^ |  | Damxung | Alpine meadow | 405 | 10.7 | Herb | Moist | Year-round | 1-1.4 | Low | 4 | Long | 55.65 | 3.77 | 3 | 36.52 | 6.78 | 3 | Fu etal.(2013) |
| AGB | g/m^2^ |  | Damxung | Alpine meadow | 405 | 10.7 | Herb | Moist | Year-round | 1-1.4 | Low | 5 | Long | 50.44 | 7.53 | 3 | 28.26 | 4.52 | 3 | Fu etal.(2013) |
| AGB | g/m^2^ |  | Damxung | Alpine meadow | 405 | 10.7 | Herb | Moist | Year-round | 1-1.4 | Low | 3 | Short | 49.57 | 1.51 | 3 | 29.57 | 6.78 | 3 | Fu etal.(2013) |
| AGB | g/m^2^ |  | Damxung | Alpine meadow | 405 | 10.7 | Herb | Moist | Year-round | 1-1.4 | Low | 4 | Long | 56.96 | 12.05 | 3 | 70.44 | 20.33 | 3 | Fu etal.(2013) |
| AGB | g/m^2^ |  | Damxung | Alpine meadow | 405 | 10.7 | Herb | Moist | Year-round | 1-1.4 | Low | 5 | Long | 47.39 | 5.27 | 3 | 42.17 | 7.53 | 3 | Fu etal.(2013) |
| AGB | g/m^2^ |  | Damxung | Alpine meadow | 405 | 10.7 | Herb | Moist | Year-round | 1-1.4 | Low | 3 | Short | 55.65 | 2.26 | 3 | 42.61 | 7.53 | 3 | Fu etal.(2013) |
| AGB | g/m^2^ |  | Damxung | Alpine meadow | 405 | 10.7 | Herb | Moist | Year-round | 1-1.4 | Low | 4 | Long | 94.35 | 18.83 | 3 | 88.70 | 6.78 | 3 | Fu etal.(2013) |
| AGB | g/m^2^ |  | Damxung | Alpine meadow | 405 | 10.7 | Herb | Moist | Year-round | 1-1.4 | Low | 5 | Long | 90.87 | 13.56 | 3 | 66.96 | 14.31 | 3 | Fu etal.(2013) |
| BGB | g/m^2^ | 0-10 | Damxung | Alpine meadow | 405 | 10.7 | Herb | Moist | Year-round | 2.00 | Low | 3 | Short | 361.57 | 172.20 | 4 | 444.75 | 143.48 | 4 | Zong etal. (2013) |
| BGB | g/m^2^ | 0-5 | Fenghuoshan | Alpine meadow | 216 | 2.5 | Herb | Moist | Year-round | 2.0 | Low | 2 | Short | 1064.36 | 351.31 | 5 | 1448.60 | 254.02 | 5 | Li etal. (2011) |
| BGB | g/m^2^ | 0-5 | Fenghuoshan | Alpine meadow | 216 | 2.5 | Herb | Moist | Year-round | 4.0 | High | 2 | Short | 1064.36 | 351.31 | 5 | 1220.58 | 149.28 | 5 | Li etal. (2011) |
| BGB | g/m^2^ | 0-10 | Haibei | Alpine meadow | 370 | 8.5 | Herb | Moist | Year-round | 1.0–2.0 | Low | 17 | Long | 1161.80 | 463.44 | 16 | 695.20 | 588.72 | 16 | Yu et al. (2015) |
| BGB | g/m^2^ | 0-10 | Haibei | Alpine meadow | 370 | 8.5 | Herb | Moist | Year-round | 1.0–2.0 | Low | 17 | Long | 1696.18 | 608.32 | 16 | 1180.98 | 650.36 | 16 | Yu et al. (2015) |
| BGB | g/m^2^ | 0-5 | Fenghuoshan | Alpine meadow | 216 | 2.5 | Herb | Moist | Year-round | 2.10 | High | 2 | Short | 755.14 | 271.95 | 3 | 1016.82 | 220.16 | 3 | Li etal. (2011) |
| BGB | g/m^2^ | 0-40 | Haibei | Alpine meadow | 370 | 8.5 | Herb | Moist | Year-round | 1.2-1.7 | Low | 1 | Short | 3170.50 | 239.20 | 4 | 3954.60 | 292.60 | 4 | Lin et al. (2011) |
| BGB | g/m^2^ | 0-40 | Haibei | Alpine meadow | 370 | 8.5 | Herb | Moist | Year-round | 1.2-1.7 | Low | 2 | Short | 3228.20 | 239.20 | 4 | 4078.70 | 425.00 | 4 | Lin et al. (2011) |
| BGB | g/m^2^ | 0-40 | Haibei | Alpine meadow | 370 | 8.5 | Herb | Moist | Year-round | 1.2-1.7 | Low | 3 | Short | 3232.80 | 265.80 | 4 | 4229.40 | 318.80 | 4 | Lin et al. (2011) |
| BGB | g/m^2^ | 0-10 | Beiluhe | Alpine meadow | 256 | 6 | Herb | Moist | Year-round | 3.00 | High | 3 | Short | 1697.97 | 285.95 | 5 | 1571.26 | 395.29 | 5 | Xu et al. (2015) |
| BGB | g/m^2^ | 0-10 | Beiluhe | Alpine meadow | 256 | 6 | Herb | Moist | Year-round | 3.00 | High | 4 | Long | 2122.67 | 197.91 | 5 | 1750.51 | 219.83 | 5 | Xu et al. (2015) |
| BGB | g/m^2^ | 0-10 | Kakagou | Alpine meadow | 574.4 | 7.33 | Herb | Moist | Year-round | 1.40 | Low | 2 | Short | 1386.40 | 1145.98 | 5 | 1301.90 | 734.32 | 5 | Shi et al. (2008) |
| BGB | g/m^2^ | 0-30 | Nam Co | Alpine steppe | 332 | 12 | Herb | Dry | Growing-season | 2 | Low | 1 | Short | 1761.60 | 383.80 | 4 | 1622.10 | 348.80 | 4 | Zhao etal.(2019) |
| BGB | g/m^2^ | 0-30 | Nam Co | Alpine steppe | 332 | 12 | Herb | Dry | Growing-season | 2 | Low | 2 | Short | 2110.50 | 383.60 | 4 | 1726.70 | 732.60 | 4 | Zhao etal.(2019) |
| BGB | g/m^2^ | 0-10 | Barrow | Arctic tundra | 57 | 3.7 | Herb+Shrub | Moist | Growing-season | 1-2 | Low | 3-4 | Long | 1091.00 | 518.90 | 12 | 1285.80 | 592.80 | 12 | Hollister etal. (2010) |
| BGB | g/m^2^ | 0-10 | Daring Lake | Arctic tundra | 138 | 5.9 | Shrub | Moist | Growing-season | >2 | High | 8 | Long | 387.32 | 51.6979 | 5 | 513.16 | 83.1817 | 5 | Zamin etal. (2014) |
| BGB | g/m^2^ | 0-5 | Fenghuoshan | Alpine swamp meadow | 216 | 1.42 | Herb | Wet | Year-round | 6.2 | Low | 4 | Long | 6358.00 | 1460.00 | 4 | 8920.00 | 1780.00 | 4 | Chen etal.(2017) |
| BGB | g/m^2^ | 0-20 | Damxung | Alpine meadow | 405 | 10.7 | Herb | Moist | Year-round | 1.6 | Low | 3 | Short | 487.34 | 177.22 | 4 | 487.34 | 126.58 | 4 | Zong etal. (2018) |
| BGB | g/m^2^ | 0-20 | Damxung | Alpine meadow | 405 | 10.7 | Herb | Moist | Year-round | 1.6 | Low | 4 | Long | 569.62 | 316.46 | 4 | 930.38 | 88.60 | 4 | Zong etal. (2018) |
| BGB | g/m^2^ | 0-20 | Damxung | Alpine meadow | 405 | 10.7 | Herb | Moist | Year-round | 1.6 | Low | 5 | Long | 550.63 | 1068.15 | 4 | 727.85 | 996.93 | 4 | Zong etal. (2018) |
| BGB | g/m^2^ | 0-20 | Damxung | Alpine meadow | 405 | 10.7 | Herb | Moist | Year-round | 1.6 | Low | 3 | Short | 380.30 | 200.80 | 5 | 475.30 | 153.62 | 5 | Zong etal. (2018) |
| BGB | g/m^2^ | 0-20 | Damxung | Alpine meadow | 405 | 10.7 | Herb | Moist | Year-round | 1.6 | Low | 4 | Long | 854.30 | 389.97 | 5 | 917.60 | 82.51 | 5 | Zong etal. (2018) |
| BGB | g/m^2^ | 0-20 | Damxung | Alpine meadow | 405 | 10.7 | Herb | Moist | Year-round | 1.6 | Low | 5 | Long | 551.20 | 425.52 | 5 | 625.10 | 472.70 | 5 | Zong etal. (2018) |
| BGB | g/m^2^ | 0-10 | Haibei | Alpine meadow | 370 | 8.5 | Herb | Moist | Year-round | >1 |  | 2 | Short | 1535.90 | 274.59 | 6 | 2096.40 | 164.85 | 6 | Chen etal.(2016) |
| BGB | g/m^2^ | 0-10 | Haibei | Alpine meadow | 370 | 8.5 | Herb | Moist | Year-round | >1 |  | 3 | Short | 1950.70 | 274.59 | 6 | 2331.80 | 521.74 | 6 | Chen etal.(2016) |
| BGB | g/m^2^ | 0-10 | Haibei | Alpine meadow | 370 | 8.5 | Herb | Moist | Year-round | >1 |  | 4 | Long | 1883.40 | 302.02 | 6 | 2230.90 | 192.28 | 6 | Chen etal.(2016) |
| BGB | g/m^2^ | 0-20 | Damxung | Alpine meadow | 405 | 10.7 | Herb | Moist | Year-round | 1-1.4 | Low | 5 | Long | 804.00 | 696.28 | 3 | 1204.00 | 346.41 | 3 | Fu etal.(2013) |
| BGB | g/m^2^ | 0-20 | Damxung | Alpine meadow | 405 | 10.7 | Herb | Moist | Year-round | 1-1.4 | Low | 5 | Long | 1860.00 | 462.46 | 3 | 2192.00 | 464.19 | 3 | Fu etal.(2013) |
| BGB | g/m^2^ | 0-20 | Damxung | Alpine meadow | 405 | 10.7 | Herb | Moist | Year-round | 1-1.4 | Low | 5 | Long | 9110.00 | 3727.37 | 3 | 13954.00 | 6526.37 | 3 | Fu etal.(2013) |
| Soil total N | g/kg soil | 0-20 | Damxung | Alpine meadow | 405 | 10.7 | Herb | Moist | Year-round | 0.6-2.0 | Low | 2 | Short | 1.85 | 0.16 | 3 | 2.00 | 0.10 | 3 | Yu et al. (2014) |
| Soil total N | g/kg soil | 0-20 | Damxung | Alpine meadow | 405 | 10.7 | Herb | Moist | Year-round | 0.6-2.0 | Low | 2 | Short | 2.20 | 0.16 | 3 | 2.26 | 0.07 | 3 | Yu et al. (2014) |
| Soil total N | g/kg soil | 0-20 | Damxung | Alpine meadow | 405 | 10.7 | Herb | Moist | Year-round | 0.6-2.0 | Low | 2 | Short | 3.29 | 0.20 | 3 | 3.61 | 0.23 | 3 | Yu et al. (2014) |
| Soil total N | g/kg soil | 0-5 | Beiluhe | Alpine meadow | 276 | 6 | Herb | Moist | Year-round | 2.70 | High | 3 | Short | 0.64 | 0.10 | 5 | 0.63 | 0.10 | 5 | Xue et al. (2015) |
| Soil total N | g/kg soil | 0-15 | Nagqu | Alpine meadow | 370 | 8.5 | Herb | Moist | Year-round | 0.8-1.1 | Low | 2 | Short | 6.78 | 2.03 | 4 | 6.76 | 1.65 | 4 | Wang et al. (2014) |
| Soil total N | g/kg soil | 0-15 | Haibei | Alpine meadow | 370 | 8.5 | Herb | Moist | Year-round | 0.8-1.1 | Low | 10 | Long | 7.33 | 1.79 | 4 | 6.95 | 1.55 | 4 | Wang et al. (2014) |
| Soil total N | % | 0-10 | Haibei | Alpine meadow | 370 | 8.5 | Herb | Moist | Growing-season | 1.2-1.7 | Low | 4 | Long | 5.10 | 3.00 | 4 | 5.10 | 2.60 | 10 | Zheng et al. (2012) |
| Soil total N | g/kg soil | 0-15 | Kakagou | Alpine meadow | 574.4 | 7.33 | Herb | Moist | Year-round | >1.8 | High | 1 | Short | 4.28 | 0.76 | 5 | 4.66 | 0.54 | 5 | Shi et al. (2012) |
| Soil total N | % | 0–10 | Haibei | Alpine meadow | 370 | 8.5 | Herb | Moist | Growing-season | 1.2-1.7 | Low | 4 | Long | 6.93 | 0.80 | 4 | 6.61 | 0.60 | 10 | Rui et al. (2011) |
| Soil total N | g/kg soil | 5 | Fenghuoshan | Alpine meadow | 270 | 2.5 | Herb | Moist | Year-round | 1.2 | Low | 7 | Long | 2.57 | 0.33 | 3 | 2.41 | 0.75 | 3 | Chang etal. (2017) |
| Soil total N | g/kg soil | 0-20 | Nagqu | Alpine meadow | 345.6 | 12.4 | Herb | Moist | Year-round | 1.05 | Low | 4 | Long | 4.56 | 0.44 | 4 | 6.10 | 0.34 | 4 | Ganjurjav etal.(2016) |
| Soil total N | g/kg soil | 0-20 | Nagqu | Alpine meadow | 345.6 | 12.4 | Herb | Moist | Year-round | 1.69 | Low | 4 | Long | 4.56 | 0.44 | 4 | 6.03 | 1.44 | 4 | Ganjurjav etal.(2016) |
| Soil total N | g/kg soil | 0-5 | Dadu River | Alpine meadow | 360 | 17.4 | Herb | Moist | Growing-season | 1.63 | Low | 3 | Short | 9.80 | 9.35 | 3 | 8.40 | 8.66 | 3 | Xiong etal. (2016) |
| Soil total N | g/kg soil | 0-15 | Nagqu | Alpine meadow | 370 | 8.5 | Herb | Moist | Year-round | 2.70 | High | 4 | Long | 4.72 | 0.24 | 4 | 4.47 | 0.26 | 4 | Wang et al. (2018) |
| Soil total N | g/kg soil | 0-15 | Nagqu | Alpine meadow | 370 | 8.5 | Herb | Moist | Year-round | 2.70 | High | 4 | Long | 4.72 | 0.24 | 4 | 4.62 | 0.24 | 4 | Wang et al. (2018) |
| Soil total N | g/m^2^ | 0-5 | Fenghuoshan | Alpine meadow | 216 | 2.5 | Herb | Moist | Year-round | 2.59 | High | 2 | Short | 37.11 | 9.58 | 3 | 39.83 | 10.90 | 3 | Li etal. (2011) |
| Soil total N | g/m^2^ | 0-5 | Fenghuoshan | Alpine meadow | 216 | 2.5 | Herb | Moist | Year-round | 5.16 | High | 2 | Short | 37.11 | 9.58 | 3 | 32.18 | 15.22 | 3 | Li etal. (2011) |
| Soil total N | g/kg soil | 0-20 | Baingoin | Alpine steppe | 267.2 | 13 | Herb | Dry | Year-round | >2.25 | High | 4 | Long | 4.01 | 0.10 | 4 | 3.72 | 0.66 | 4 | Ganjurjav etal.(2016) |
| Soil total N | g/kg soil | 0-20 | Baingoin | Alpine steppe | 267.2 | 13 | Herb | Dry | Year-round | >2.25 | High | 4 | Long | 4.01 | 0.10 | 4 | 3.61 | 0.18 | 4 | Ganjurjav etal.(2016) |
| Soil total N | g/kg soil | 0-10 | Gangcha | Alpine steppe | 348 | 11.4 | Herb | Dry | Year-round | >2 | High | 3 | Short | 3.80 | 0.63 | 10 | 3.80 | 0.63 | 10 | Wang etal. (2018) |
| Soil total N | g/kg soil | 0-10 | Gangcha | Alpine steppe | 348 | 11.4 | Herb | Dry | Year-round | >2 | High | 4 | Long | 3.80 | 0.32 | 10 | 3.80 | 0.32 | 10 | Wang etal. (2018) |
| Soil total N | g/kg soil | 0-20 | Haiyan | Alpine meadow | 280 | 10.5 | Herb | Moist | Year-round | 2 | Low | 1 | Short | 0.28 | 0.03 | 3 | 0.32 | 0.02 | 3 | Zhao etal.(2017) |
| Soil total N | g/kg soil | 0-20 | Gonghe | Alpine steppe | 302.00 | 11.10 | Herb | Dry | Year-round | 2 | Low | 1 | Short | 0.21 | 0.02 | 3 | 0.27 | 0.03 | 3 | Zhao etal.(2017) |
| Soil total N | g/kg soil | 0-12 | Latnjajaure field station | Alpine meadow | 202 | 8.3 | Herb | Wet | Year-round | 1.5-3 |  | 19 | Long | 15.26 | 3.47 | 4 | 15.93 | 5.61 | 4 | Alatalo etal. (2017) |
| Soil total N | g/kg soil | 0-12 | Latnjajaure field station | Alpine meadow | 202 | 8.3 | Herb+Shrub | Mesic | Year-round | 1.5-3 |  | 21 | Long | 10.37 | 5.80 | 4 | 10.79 | 8.37 | 4 | Alatalo etal. (2017) |
| Soil total N | g/kg soil | 0-12 | Latnjajaure field station | Arctic heath | 202 | 8.3 | Shrub | Dry | Year-round | 1.5-3 |  | 19 | Long | 4.10 | 1.68 | 4 | 5.93 | 3.08 | 4 | Alatalo etal. (2017) |
| Soil total N | g/kg soil | 0-10 | Alexandra Fiord | Arctic tundra | 30 | 8.7 | Shrub | Dry | Year-round | 0.2 | Low | 9 | Long | 1.30 | 0.35 | 3 | 2.10 | 0.35 | 3 | Welker etal. (2004) |
| Soil total N | g/kg soil | 0-10 | Alexandra Fiord | Arctic tundra | 30 | 7.9 | Shrub | Moist | Year-round | 0.2 | Low | 9 | Long | 14.70 | 4.20 | 4 | 12.50 | 9.00 | 4 | Welker etal. (2004) |
| Soil total N | g/kg soil | 0-10 | Alexandra Fiord | Arctic tundra | 30 | 8.1 | Herb | Wet | Year-round | 0.2 | Low | 9 | Long | 30.30 | 11.43 | 3 | 28.30 | 1.56 | 3 | Welker etal. (2004) |
| Soil total N | g/kg soil | 0-20 | Changbai Mountain | Alpine tundra | 1120 | 8.7 | Shrub | Moist | Growing-season-warming | 1 | Low | 2 | Short | 4.30 | 0.26 | 3 | 3.60 | 0.33 | 3 | Wang etal. (2014) |
| Soil total N | g/kg soil | 0–5 | Seida | Arctic tundra | 450 | 12.5 | Moss | Wet | Growing-season | 0.95 | Low | 3 | Short | 2.60 | 0.22 | 5 | 2.70 | 0.22 | 5 | Voigt etal. (2017) |
| Soil total N | g/kg soil | 0–5 | Seida | Arctic tundra | 450 | 13.4 | Shrub | Wet | Growing-season | 0.95 | Low | 3 | Short | 12.00 | 2.24 | 5 | 12.00 | 2.24 | 5 | Voigt etal. (2017) |
| Soil total N | g/kg soil | B | Seida | Arctic tundra | 450 | 12.5 | Shrub | Dry | Growing-season | 0.95 | Low | 3 | Short | 4.00 | 6.71 | 5 | 2.00 | 2.24 | 5 | Voigt etal. (2017) |
| Soil total N | g/kg soil | Organic soil | Kilpisjäarvi | Subarctic heath | 514 | 10.1 | Shrub | Moist | Year-round | 1.5 | Low | 19 | Long | 15.00 | 7.80 | 4 | 12.60 | 6.20 | 4 | Ylanne etal. (2015) |
| Soil total N | g/kg soil | Mineral soil | Kilpisjäarvi | Subarctic heath | 514 | 10.1 | Shrub | Moist | Year-round | 1.5 | Low | 19 | Long | 1.10 | 0.40 | 4 | 0.90 | 0.20 | 4 | Ylanne etal. (2015) |
| Soil total N | g/kg soil | Surface organic soil | Toolik Lake | Arctic tundra | 180.00 | 10.00 | Herb+Shrub | Moist | Growing-season | >2 | High | 20 | Long | 11.00 | 0.80 | 4 | 11.00 | 1.60 | 4 | Sistla etal. (2013) |
| Soil total N | %SOM | 0–5 | Abisko | Subarctic heath | 120.00 | 10.90 | Shrub | Wet | Growing-season | 3-4 | High | 7 | Long | 15.90 | 6.37 | 6 | 13.40 | 1.96 | 6 | Rinnan etal.(2008) |
| Soil total N | g/kg soil | 2–7 | Abisko | Subarctic heath | 120.00 | 10.90 | Moss | Wet | Growing-season | 3-4 | High | 10 | Long | 7.80 | 1.22 | 6 | 9.30 | 22.78 | 6 | Sorensen etal.(2011) |
| Soil total N | g/kg soil | 2–7 | Abisko | Subarctic heath | 120.00 | 11.00 | Moss | Wet | Growing-season | 3-4 | High | 10 | Long | 6.30 | 1.22 | 6 | 8.10 | 19.84 | 6 | Sorensen etal.(2011) |
| Soil total N | g/kg soil | 0-5 | Fenghuoshan | Alpine swamp meadow | 216 | 1.42 | Herb | Wet | Year-round | 6.2 | High | 4 | Long | 2.85 | 0.40 | 4 | 3.05 | 0.52 | 4 | Chen etal.(2017) |
| Soil total N | g/kg soil | 0-10 | Damxung | Alpine meadow | 405 | 10.7 | Herb | Moist | Year-round | >1.23 |  | 8 | Long | 2.07 | 0.44 | 4 | 2.18 | 0.17 | 4 | Yu etal. (2019) |
| Soil total N | g/kg soil | 0-10 | Damxung | Alpine meadow | 405 | 10.7 | Herb | Moist | Year-round | >1.33 |  | 8 | Long | 2.52 | 0.06 | 4 | 2.60 | 0.22 | 4 | Yu etal. (2019) |
| Soil total N | g/kg soil | 0-10 | Damxung | Alpine meadow | 405 | 10.7 | Herb | Moist | Year-round | >1.24 |  | 8 | Long | 4.62 | 0.50 | 4 | 4.48 | 0.77 | 4 | Yu etal. (2019) |
| Soil total N | g/kg soil | 0-10 | Beiluhe | Alpine swamp meadow | 306 | 6 | Herb | Wet | Year-round | >1.7 | High | 3 | Short | 1.61 | 0.36 | 3 | 1.96 | 0.48 | 3 | Zhang etal. (2014) |
| Soil total N | g/kg soil | 0-10 | Beiluhe | Alpine meadow | 306 | 6 | Herb | Moist | Year-round | >2.3 | High | 3 | Short | 1.25 | 0.10 | 3 | 1.23 | 0.26 | 3 | Zhang etal. (2014) |
| Soil total N | g/kg soil | 0-10 | Beiluhe | Alpine steppe | 306 | 6 | Herb+Shrub | Dry | Year-round | >1.7 | High | 3 | Short | 0.39 | 0.12 | 3 | 0.40 | 0.09 | 3 | Zhang etal. (2014) |
| NH_4_^+^-N | mg /kg soil | 0-20 | Damxung | Alpine meadow | 405 | 10.7 | Herb | Moist | Year-round | 0.6-2.0 | Low | 2 | Short | 5.93 | 2.28 | 3 | 4.75 | 2.09 | 3 | Yu et al. (2014) |
| NH_4_^+^-N | mg /kg soil | 0-20 | Damxung | Alpine meadow | 405 | 10.7 | Herb | Moist | Year-round | 0.6-2.0 | Low | 2 | Short | 5.55 | 2.34 | 3 | 4.72 | 2.03 | 3 | Yu et al. (2014) |
| NH_4_^+^-N | mg /kg soil | 0-20 | Damxung | Alpine meadow | 405 | 10.7 | Herb | Moist | Year-round | 0.6-2.0 | Low | 2 | Short | 14.81 | 5.55 | 3 | 14.76 | 3.11 | 3 | Yu et al. (2014) |
| NH_4_^+^-N | mg /kg soil | 0-5 | Beiluhe | Alpine meadow | 276 | 6 | Herb | Moist | Year-round | 2.70 | High | 3 | Short | 10.13 | 1.27 | 5 | 9.67 | 0.36 | 5 | Xue et al. (2015) |
| NH_4_^+^-N | mg /kg soil | 0-15 | Nagqu | Alpine meadow | 370 | 8.5 | Herb | Moist | Year-round | 0.8-1.1 | Low | 2 | Short | 47.68 | 2.79 | 4 | 53.28 | 3.61 | 4 | Wang et al. (2014) |
| NH_4_^+^-N | mg /kg soil | 0-15 | Haibei | Alpine meadow | 370 | 8.5 | Herb | Moist | Year-round | 0.8-1.1 | Low | 10 | Long | 51.14 | 3.35 | 4 | 56.84 | 2.31 | 4 | Wang et al. (2014) |
| NH_4_^+^-N | mg /kg soil | 0-10 | Haibei | Alpine meadow | 370 | 8.5 | Herb | Moist | Growing-season | 1.2-1.7 | Low | 4 | Long | 28.20 | 4.20 | 4 | 23.90 | 8.20 | 4 | Zheng et al. (2012) |
| NH_4_^+^-N | mg /kg soil | 0-15 | Kakagou | Alpine meadow | 574.4 | 7.33 | Herb | Moist | Year-round | >1.8 | High | 1 | Short | 10.79 | 1.50 | 5 | 13.37 | 1.74 | 5 | Shi et al. (2012) |
| NH_4_^+^-N | mg /kg soil | 0–10 | Haibei | Alpine meadow | 370 | 8.5 | Herb | Moist | Growing-season | 1.2-1.7 | Low | 4 | Long | 25.59 | 2.61 | 4 | 21.83 | 4.25 | 4 | Rui et al. (2011) |
| NH_4_^+^-N | mg /kg soil | 5 | Fenghuoshan | Alpine meadow | 270 | 2.5 | Herb | Moist | Year-round | 1.2 | Low | 7 | Long | 12.18 | 3.02 | 3 | 5.70 | 1.42 | 3 | Chang etal. (2017) |
| NH_4_^+^-N | mg /kg soil | 0-5 | Dadu River | Alpine meadow | 360 | 17.4 | Herb | Moist | Growing-season | 1.63 | Low | 3 | Short | 13.42 | 2.10 | 3 | 5.88 | 4.28 | 3 | Xiong etal. (2016) |
| NH_4_^+^-N | mg /kg soil | 0-15 | Nagqu | Alpine meadow | 370 | 8.5 | Herb | Moist | Year-round | 2.70 | High | 4 | Long | 27.97 | 4.62 | 4 | 30.09 | 2.74 | 4 | Wang et al. (2018) |
| NH_4_^+^-N | mg /kg soil | 0-15 | Nagqu | Alpine meadow | 370 | 8.5 | Herb | Moist | Year-round | 2.70 | High | 4 | Long | 27.97 | 4.62 | 4 | 33.09 | 4.62 | 4 | Wang et al. (2018) |
| NH_4_^+^-N | mg /kg soil | 0-20 | Haiyan | Alpine meadow | 280 | 10.5 | Herb | Moist | Year-round | 2 | Low | 1 | Short | 0.34 | 0.05 | 3 | 1.11 | 0.33 | 3 | Zhao etal.(2017) |
| NH_4_^+^-N | mg /kg soil | 0-20 | Gonghe | Alpine steppe | 302.00 | 11.10 | Herb | Dry | Year-round | 2 | Low | 1 | Short | 0.68 | 0.10 | 3 | 1.10 | 0.14 | 3 | Zhao etal.(2017) |
| NH_4_^+^-N | mg /kg soil | 0-10 | Gangcha | Alpine steppe | 348 | 11.4 | Herb | Dry | Year-round | >2 | High | 2 | Short | 1.80 | 1.20 | 10 | 3.00 | 1.90 | 10 | Wang etal. (2018) |
| NH_4_^+^-N | mg /kg soil | 0-10 | Gangcha | Alpine steppe | 348 | 11.4 | Herb | Dry | Year-round | >2 | High | 3 | Short | 2.20 | 0.79 | 10 | 3.00 | 0.60 | 10 | Wang etal. (2018) |
| NH_4_^+^-N | mg /kg soil |  | Alexandra Fiord | Arctic tundra | 30 | 7.9 | Shrub | Dry | Year-round | 1–2 | Low | 16 | Long | 65.42 | 139.20 | 5 | 66.56 | 142.57 | 5 | Lamb etal. (2011) |
| NH_4_^+^-N | g/m^2^ |  | N-Siberia | Arctic tundra | 310 | 11 | Shrub | Dry | Year-round | 3.60 | High | 2 | Short | 0.21 | 0.25 | 9 | 0.40 | 0.34 | 9 | Biasi etal. (2006) |
| NH_4_^+^-N | mg /kg soil | 0–5 | Seida | Arctic tundra | 450 | 12.5 | Moss | Wet | Growing-season | 0.95 | Low | 2 | Short | 49.00 | 38.01 | 5 | 42.00 | 17.89 | 5 | Voigt etal. (2017) |
| NH_4_^+^-N | mg /kg soil | 0–5 | Seida | Arctic tundra | 450 | 13.4 | Shrub | Wet | Growing-season | 0.95 | Low | 2 | Short | 40.00 | 20.12 | 5 | 17.00 | 11.18 | 5 | Voigt etal. (2017) |
| NH_4_^+^-N | mg /kg soil | B | Seida | Arctic tundra | 450 | 12.5 | Shrub | Dry | Growing-season | 0.95 | Low | 2 | Short | 10.00 | 6.71 | 5 | 10.00 | 4.47 | 5 | Voigt etal. (2017) |
| NH_4_^+^-N | mg /kg soil | 0–5 | Seida | Arctic tundra | 450 | 12.5 | Moss | Wet | Growing-season | 0.95 | Low | 3 | Short | 94.00 | 82.73 | 5 | 71.00 | 53.67 | 5 | Voigt etal. (2017) |
| NH_4_^+^-N | mg /kg soil | 0–5 | Seida | Arctic tundra | 450 | 13.4 | Shrub | Wet | Growing-season | 0.95 | Low | 3 | Short | 36.00 | 17.89 | 5 | 26.00 | 15.65 | 5 | Voigt etal. (2017) |
| NH_4_^+^-N | mg /kg soil | B | Seida | Arctic tundra | 450 | 12.5 | Shrub | Dry | Growing-season | 0.95 | Low | 3 | Short | 5.00 | 4.47 | 5 | 3.30 | 0.67 | 5 | Voigt etal. (2017) |
| NH_4_^+^-N | μmol/bag | 0-10 | Toolik Lake | Arctic tundra | 180.00 | 10.00 | Herb+Shrub | Moist | Growing-season | 4-5 | High | 2 | Short | 3.50 | 1.83 | 5 | 3.24 | 1.30 | 5 | Hobbie etal. (1998) |
| NH_4_^+^-N | μmol/bag | 0-10 | Toolik Lake | Arctic tundra | 180.00 | 10.00 | Herb+Shrub | Moist | Growing-season | 4-5 | High | 2 | Short | 10.26 | 10.24 | 5 | 8.59 | 2.82 | 5 | Hobbie etal. (1998) |
| NH_4_^+^-N | mg N/m^2^ | 0-10 | Daring Lake | Arctic tundra | 138 | 5.9 | Shrub | Moist | Growing-season | >2 | High | 8 | Long | 17.56 | 16.55 | 5 | 18.20 | 16.55 | 5 | Zamin etal. (2014) |
| NH_4_^+^-N | mg /kg soil | Organic soil | Toolik Lake | Arctic tundra | 180.00 | 10.00 | Herb | Wet | Growing-season | 3.5 | High | 8 | Long | 2.20 | 0.85 | 8 | 3.30 | 1.98 | 8 | Chapin III etal. (1995) |
| NH_4_^+^-N | mg /kg soil | Organic soil | Toolik Lake | Arctic tundra | 180.00 | 10.00 | Shrub | Dry | Growing-season | 3.39 | High | 9 | Long | 60.04 | 5.59 | 2 | 65.54 | 20.04 | 2 | Demarco etal. (2014) |
| NH_4_^+^-N | mg/kg-SOM | Organic soil | Kilpisjäarvi | Subarctic heath | 514 | 10.1 | Shrub | Moist | Year-round | 1.20 | Low | 10 | Long | 8.85 | 4.06 | 3 | 6.26 | 1.45 | 3 | Rinnan etal.(2009) |
| NH_4_^+^-N | mg/kg-SOM | 0–5 | Abisko | Subarctic heath | 120.00 | 10.90 | Shrub | Wet | Growing-season | 3-4 | High | 7 | Long | 1.75 | 1.00 | 6 | 1.46 | 0.88 | 6 | Rinnan etal.(2008) |
| NH_4_^+^-N | mg /kg soil | 0-5 | Fenghuoshan | Alpine swamp meadow | 216 | 1.42 | Herb | Wet | Year-round | 6.2 | High | 4 | Long | 34.37 | 13.12 | 4 | 60.89 | 19.36 | 4 | Chen etal.(2017) |
| NH_4_^+^-N | mg /kg soil | 0-10 | Damxung | Alpine meadow | 405 | 10.7 | Herb | Moist | Year-round | 2.95 | High | 3 | Short | 5.42 | 1.10 | 3 | 23.61 | 6.07 | 3 | Fu etal. (2019) |
| NH_4_^+^-N | mg /kg soil | 0-10 | Damxung | Alpine meadow | 405 | 10.7 | Herb | Moist | Year-round | 2.76 | High | 3 | Short | 5.42 | 1.10 | 3 | 16.67 | 7.47 | 3 | Fu etal. (2019) |
| NO_3_^-^-N | mg /kg soil | 0-20 | Damxung | Alpine meadow | 405 | 10.7 | Herb | Moist | Year-round | 0.6-2.0 | Low | 2 | Short | 6.11 | 3.96 | 3 | 3.94 | 2.05 | 3 | Yu et al. (2014) |
| NO_3_^-^-N | mg /kg soil | 0-20 | Damxung | Alpine meadow | 405 | 10.7 | Herb | Moist | Year-round | 0.6-2.0 | Low | 2 | Short | 6.41 | 2.62 | 3 | 4.54 | 1.97 | 3 | Yu et al. (2014) |
| NO_3_^-^-N | mg /kg soil | 0-20 | Damxung | Alpine meadow | 405 | 10.7 | Herb | Moist | Year-round | 0.6-2.0 | Low | 2 | Short | 4.99 | 2.32 | 3 | 5.86 | 2.32 | 3 | Yu et al. (2014) |
| NO_3_^-^-N | mg /kg soil | 0-5 | Beiluhe | Alpine meadow | 276 | 6 | Herb | Moist | Year-round | 2.70 | High | 3 | Short | 3.28 | 0.64 | 5 | 3.39 | 0.42 | 5 | Xue et al. (2015) |
| NO_3_^-^-N | mg /kg soil | 0-15 | Nagqu | Alpine meadow | 370 | 8.5 | Herb | Moist | Year-round | 0.8-1.1 | Low | 2 | Short | 41.33 | 3.17 | 4 | 44.42 | 1.22 | 4 | Wang et al. (2014) |
| NO_3_^-^-N | mg /kg soil | 0-15 | Haibei | Alpine meadow | 370 | 8.5 | Herb | Moist | Year-round | 0.8-1.1 | Low | 10 | Long | 39.79 | 3.80 | 4 | 42.40 | 3.03 | 4 | Wang et al. (2014) |
| NO_3_^-^-N | mg /kg soil | 0-15 | Kakagou | Alpine meadow | 574.4 | 7.33 | Herb | Moist | Year-round | >1.8 | High | 1 | Short | 6.39 | 0.20 | 5 | 8.01 | 1.14 | 5 | Shi et al. (2012) |
| NO_3_^-^-N | mg /kg soil | 0–10 | Haibei | Alpine meadow | 370 | 8.5 | Herb | Moist | Growing-season | 1.2-1.7 | Low | 4 | Long | 3.72 | 1.44 | 4 | 5.40 | 0.72 | 4 | Rui et al. (2011) |
| NO_3_^-^-N | ng N/(day · g resin) | 0–10 | Haibei | Alpine meadow | 370 | 8.5 | Herb | Moist | Year-round | 0.56 | Low | 4 | Long | 96.54 | 73.89 | 12 | 54.15 | 37.10 | 12 | Wu et al. (2016) |
| NO_3_^-^-N | mg /kg soil | 0-5 | Dadu River | Alpine meadow | 360 | 17.4 | Herb | Moist | Growing-season | 1.63 | Low | 3 | Short | 13.41 | 15.19 | 3 | 9.75 | 3.90 | 3 | Xiong etal. (2016) |
| NO_3_^-^-N | mg /kg soil | 0-15 | Nagqu | Alpine meadow | 370 | 8.5 | Herb | Moist | Year-round | 2.70 | High | 4 | Long | 14.66 | 1.28 | 4 | 15.70 | 1.08 | 4 | Wang et al. (2018) |
| NO_3_^-^-N | mg /kg soil | 0-15 | Nagqu | Alpine meadow | 370 | 8.5 | Herb | Moist | Year-round | 2.70 | High | 4 | Long | 14.66 | 1.28 | 4 | 15.79 | 1.28 | 4 | Wang et al. (2018) |
| NO_3_^-^-N | mg /kg soil | 0-20 | Haiyan | Alpine meadow | 280 | 10.5 | Herb | Moist | Year-round | 2 | Low | 1 | Short | 5.82 | 1.42 | 3 | 3.99 | 1.77 | 3 | Zhao etal.(2017) |
| NO_3_^-^-N | mg /kg soil | 0-20 | Gonghe | Alpine steppe | 302.00 | 11.10 | Herb | Dry | Year-round | 2 | Low | 1 | Short | 3.17 | 0.73 | 3 | 4.26 | 1.37 | 3 | Zhao etal.(2017) |
| NO_3_^-^-N | mg /kg soil | 0-10 | Gangcha | Alpine steppe | 348 | 11.4 | Herb | Dry | Year-round | >2 | High | 2 | Short | 25.00 | 5.06 | 10 | 23.50 | 4.11 | 10 | Wang etal. (2018) |
| NO_3_^-^-N | mg /kg soil | 0-10 | Gangcha | Alpine steppe | 348 | 11.4 | Herb | Dry | Year-round | >2 | High | 3 | Short | 5.96 | 1.26 | 10 | 4.04 | 1.26 | 10 | Wang etal. (2018) |
| NO_3_^-^-N | mg /kg soil |  | Alexandra Fiord | Arctic tundra | 30 | 7.9 | Shrub | Dry | Year-round | 1–2 | Low | 16 | Long | 27.65 | 80.59 | 5 | 25.14 | 52.46 | 5 | Lamb etal. (2011) |
| NO_3_^-^-N | g/m^2^ |  | N-Siberia | Arctic tundra | 310 | 11 | Shrub | Dry | Year-round | 3.60 | High | 2 | Short | 0.09 | 0.09 | 9 | 0.17 | 0.17 | 9 | Biasi etal. (2006) |
| NO_3_^-^-N | mg /kg soil | 0–5 | Seida | Arctic tundra | 450 | 12.5 | Moss | Wet | Growing-season | 0.95 | Low | 2 | Short | 118.00 | 51.43 | 5 | 157.00 | 26.83 | 5 | Voigt etal. (2017) |
| NO_3_^-^-N | mg /kg soil | 0–5 | Seida | Arctic tundra | 450 | 13.4 | Shrub | Wet | Growing-season | 0.95 | Low | 2 | Short | 0.80 | 0.67 | 5 | 1.10 | 0.67 | 5 | Voigt etal. (2017) |
| NO_3_^-^-N | mg /kg soil | B | Seida | Arctic tundra | 450 | 12.5 | Shrub | Dry | Growing-season | 0.95 | Low | 2 | Short | 0.60 | 0.67 | 5 | 1.40 | 1.57 | 5 | Voigt etal. (2017) |
| NO_3_^-^-N | mg /kg soil | 0–5 | Seida | Arctic tundra | 450 | 12.5 | Moss | Wet | Growing-season | 0.95 | Low | 3 | Short | 419.00 | 120.75 | 5 | 308.00 | 114.04 | 5 | Voigt etal. (2017) |
| NO_3_^-^-N | mg /kg soil | 0–5 | Seida | Arctic tundra | 450 | 13.4 | Shrub | Wet | Growing-season | 0.95 | Low | 3 | Short | 3.20 | 2.46 | 5 | 1.30 | 1.79 | 5 | Voigt etal. (2017) |
| NO_3_^-^-N | mg /kg soil | B | Seida | Arctic tundra | 450 | 12.5 | Shrub | Dry | Growing-season | 0.95 | Low | 3 | Short | 1.30 | 1.79 | 5 | 0.40 | 0.45 | 5 | Voigt etal. (2017) |
| NO_3_^-^-N | μmol/bag | 0-10 | Toolik Lake | Arctic tundra | 180.00 | 10.00 | Herb+Shrub | Moist | Growing-season | 4-5 | High | 2 | Short | 0.38 | 0.16 | 5 | 0.27 | 0.11 | 5 | Hobbie etal. (1998) |
| NO_3_^-^-N | μmol/bag | 0-10 | Toolik Lake | Arctic tundra | 180.00 | 10.00 | Herb+Shrub | Moist | Growing-season | 4-5 | High | 2 | Short | 0.36 | 0.20 | 5 | 0.35 | 0.20 | 5 | Hobbie etal. (1998) |
| NO_3_^-^-N | mg N/m^2^ | 0-10 | Daring Lake | Arctic tundra | 138 | 5.9 | Shrub | Moist | Growing-season | >2 | High | 8 | Long | 0.46 | 0.25 | 5 | 1.38 | 1.04 | 5 | Zamin etal. (2014) |
| NO_3_^-^-N | mg /kg soil | Organic | Toolik Lake | Arctic tundra | 180.00 | 10.00 | Shrub | Dry | Growing-season | 3.39 | High | 9 | Long | 1.73 | 1.09 | 2 | 0.50 | 0.01 | 2 | Demarco etal. (2014) |
| NO_3_^-^-N | mg /kg soil | 0-5 | Fenghuoshan | Alpine swamp meadow | 216 | 1.42 | Herb | Wet | Year-round | 6.2 | High | 4 | Long | 14.09 | 6.44 | 4 | 25.14 | 13.02 | 4 | Chen etal.(2017) |
| NO_3_^-^-N | mg /kg soil | 0-10 | Damxung | Alpine meadow | 405 | 10.7 | Herb | Moist | Year-round | 2.76 | High | 3 | Short | 6.65 | 2.11 | 3 | 12.20 | 4.20 | 3 | Fu etal. (2019) |
| MBC | mg /kg soil | 0-15 | Nagqu | Alpine meadow | 370 | 8.5 | Herb | Moist | Year-round | 0.8-1.1 | Low | 2 | Short | 538.63 | 4.70 | 4 | 541.60 | 28.88 | 4 | Wang et al. (2014) |
| MBC | mg /kg soil | 0-15 | Haibei | Alpine meadow | 370 | 8.5 | Herb | Moist | Year-round | 0.8-1.1 | Low | 10 | Long | 522.25 | 39.60 | 4 | 496.31 | 39.86 | 4 | Wang et al. (2014) |
| MBC | mg /kg soil | 0-10 | Haibei | Alpine meadow | 370 | 8.5 | Herb | Moist | Growing-season | 1.2-1.7 | Low | 4 | Long | 1415.00 | 1454.00 | 4 | 1491.00 | 1260.00 | 4 | Zheng et al. (2012) |
| MBC | mg /kg soil | 0–20 | Damxung | Alpine meadow | 405 | 10.7 | Herb | Moist | Year-round | 1.26 | Low | 2 | Short | 344.01 | 87.56 | 3 | 286.61 | 32.62 | 3 | Fu et al. (2012) |
| MBC | mg /kg soil | 0–20 | Damxung | Alpine meadow | 405 | 10.7 | Herb | Moist | Year-round | 0.98 | Low | 2 | Short | 337.76 | 51.04 | 3 | 304.18 | 27.74 | 3 | Fu et al. (2012) |
| MBC | mg /kg soil | 0–20 | Damxung | Alpine meadow | 405 | 10.7 | Herb | Moist | Year-round | 1.37 | Low | 2 | Short | 751.92 | 166.41 | 3 | 566.85 | 38.27 | 3 | Fu et al. (2012) |
| MBC | mg /kg soil | 0–10 | Haibei | Alpine meadow | 370 | 8.5 | Herb | Moist | Growing-season | 1.2-1.7 | Low | 7 | Long | 622.81 | 74.56 | 4 | 778.51 | 135.96 | 4 | Ma et al. (2015) |
| MBC | mg /kg soil | 0-15 | Kakagou | Alpine meadow | 574.4 | 7.33 | Herb | Moist | Year-round | >1.8 | High | 1 | Short | 901.93 | 138.34 | 5 | 1149.70 | 158.06 | 5 | Shi et al. (2012) |
| MBC | mg /kg soil | 0–10 | Haibei | Alpine meadow | 370 | 8.5 | Herb | Moist | Growing-season | 1.2-1.7 | Low | 4 | Long | 2245.00 | 644.00 | 4 | 2211.00 | 738.00 | 4 | Rui et al. (2011) |
| MBC | mg /kg soil | 5-15 | Qilian Mountain | Alpine meadow | 330 | 8.5 | Herb | Moist | Year-round | 1.5-2.5 |  | 3 | Short | 747.23 | 175.25 | 9 | 752.58 | 371.23 | 9 | Heng et al. (2011) |
| MBC | mg /kg soil | 0-10 | Haibei | Alpine meadow | 370 | 8.5 | Herb | Moist | Year-round | 1.60 | Low | 3 | Short | 646.51 | 74.42 | 4 | 548.84 | 37.20 | 4 | Yang et al. (2017) |
| MBC | mg /kg soil | 0-15 | Nagqu | Alpine meadow | 370 | 8.5 | Herb | Moist | Year-round | 2.70 | High | 4 | Long | 264.58 | 26.54 | 4 | 257.39 | 32.72 | 4 | Wang et al. (2018) |
| MBC | mg /kg soil | 0-15 | Nagqu | Alpine meadow | 370 | 8.5 | Herb | Moist | Year-round | 2.70 | High | 4 | Long | 264.58 | 26.54 | 4 | 272.17 | 26.54 | 4 | Wang et al. (2018) |
| MBC | mg /kg soil | 0-10 | Haibei | Alpine meadow | 370 | 8.5 | Herb | Moist | Growing-season | 1.2-1.7 | Low | 4 | Long | 1964.00 | 222.03 | 2 | 1678.00 | 110.31 | 2 | Jing et al. (2014) |
| MBC | mg /kg soil | 0-10 | Gangcha | Alpine steppe | 348 | 11.4 | Herb | Dry | Year-round | >2 | High | 3 | Short | 733.21 | 22.58 | 10 | 751.86 | 89.40 | 10 | Wang etal. (2018) |
| MBC | mg /kg soil | 0-10 | Gangcha | Alpine steppe | 348 | 11.4 | Herb | Dry | Year-round | >2 | High | 4 | Long | 834.09 | 94.87 | 10 | 846.59 | 81.43 | 10 | Wang etal. (2018) |
| MBC | mg /kg soil | 0-10 | Nam Co | Alpine steppe | 332 | 12 | Herb | Dry | Growing-season | 2 | Low | 2 | Short | 350.50 | 89.10 | 4 | 252.48 | 53.46 | 4 | Zhao etal.(2019) |
| MBC | mg /kg soil | 0-10 | Gangcha | Alpine steppe | 348 | 11.4 | Herb | Dry | Year-round | >2 | High | 2 | Short | 890.40 | 74.95 | 10 | 801.90 | 129.02 | 10 | Wang etal. (2018) |
| MBC | mg /kg soil | 0-10 | Gangcha | Alpine steppe | 348 | 11.4 | Herb | Dry | Year-round | >2 | High | 3 | Short | 732.60 | 35.73 | 10 | 720.10 | 58.82 | 10 | Wang etal. (2018) |
| MBC | mg/g SOM | Organic soil | Toolik Lake | Arctic tundra | 120.00 | 9.9 | Shrub | Dry | Growing-season | 1-2 | Low | 8 | Long | 8.00 | 1.41 | 2 | 8.10 | 1.41 | 2 | Schmidt etal. (2002) |
| MBC | mg/g SOM | Organic soil | Toolik Lake | Arctic tundra | 120.00 | 5.8 | Shrub | Dry | Growing-season | 1-2 | Low | 8 | Long | 9.25 | 0.07 | 2 | 8.90 | 0.99 | 2 | Schmidt etal. (2002) |
| MBC | mg/g SOM | Organic soil | Toolik Lake | Arctic tundra | 120.00 | 9.9 | Shrub | Moist | Growing-season | 1-2 | Low | 9 | Long | 18.10 | 0.57 | 2 | 17.05 | 2.33 | 2 | Schmidt etal. (2002) |
| MBC | mg/g SOM | Organic soil | Toolik Lake | Arctic tundra | 120.00 | 9.9 | Herb | Wet | Growing-season | 1-2 | Low | 9 | Long | 4.55 | 0.21 | 2 | 4.20 | 0.71 | 2 | Schmidt etal. (2002) |
| MBC | mg /kg soil | Surface organic soil | Toolik Lake | Arctic tundra | 180.00 | 10.00 | Herb+Shrub | Moist | Growing-season | >2 | High | 20 | Long | 6050.00 | 2474.00 | 4 | 6315.00 | 2732.00 | 4 | Sistla etal. (2013) |
| MBC | g/kg-SOM | Organic soil | Kilpisjäarvi | Subarctic heath | 514 | 10.1 | Shrub | Moist | Year-round | 1.20 | Low | 10 | Long | 5.82 | 1.05 | 2 | 5.04 | 1.37 | 2 | Rinnan etal.(2009) |
| MBC | g/kg-SOM | 0–5 | Abisko | Subarctic heath | 120.00 | 10.90 | Shrub | Wet | Growing-season | 3-4 | High | 7 | Long | 10.69 | 1.59 | 6 | 10.51 | 1.49 | 6 | Rinnan etal.(2008) |
| MBC | mg /kg soil | 0-5 | Fenghuoshan | Alpine swamp meadow | 216 | 1.42 | Herb | Wet | Year-round | 6.2 | High | 4 | Long | 223.85 | 223.86 | 4 | 309.73 | 209.50 | 4 | Chen etal.(2017) |
| MBC | mg /kg soil | 0-5 | Fenghuoshan | Alpine swamp meadow | 216 | 1.42 | Herb | Wet | Year-round | 2.54 | High | 3 | Short | 602.70 | 67.53 | 5 | 808.70 | 78.71 | 5 | Li etal.(2010) |
| MBC | mg /kg soil | 0-5 | Fenghuoshan | Alpine swamp meadow | 216 | 1.42 | Herb | Wet | Year-round | 4.99 | High | 3 | Short | 602.70 | 67.53 | 5 | 354.00 | 48.30 | 5 | Li etal.(2010) |
| MBC | mg /kg soil | 0-5 | Fenghuoshan | Alpine meadow | 216 | 2.5 | Herb | Moist | Year-round | 2 | Low | 3 | Short | 230.00 | 44.72 | 5 | 385.30 | 42.49 | 5 | Li etal.(2010) |
| MBC | mg /kg soil | 0-5 | Fenghuoshan | Alpine meadow | 216 | 2.5 | Herb | Moist | Year-round | 4 | High | 3 | Short | 230.00 | 44.72 | 5 | 192.70 | 67.75 | 5 | Li etal.(2010) |
| MBC | mg /kg soil | 0-20 | Fenghuoshan | Alpine swamp meadow | 216 | 1.42 | Herb | Wet | Year-round | 1.5-2.5 |  | 3 | Short | 255.95 | 117.22 | 3 | 289.19 | 90.13 | 3 | Xi etal.(2019) |
| MBC | mg /kg soil | 0-20 | Fenghuoshan | Alpine swamp meadow | 216 | 1.42 | Herb | Wet | Year-round | 3-5 | High | 3 | Short | 255.95 | 117.22 | 3 | 254.54 | 90.93 | 3 | Xi etal.(2019) |
| MBC | mg /kg soil | 0-5 | Fenghuoshan | Alpine swamp meadow | 216 | 1.42 | Herb | Wet | Year-round | 2.28 | High | 2 | Short | 602.68 | 52.31 | 3 | 808.69 | 60.97 | 3 | Li etal. (2011) |
| MBC | mg /kg soil | 0-20 | Damxung | Alpine meadow | 405 | 10.7 | Herb | Moist | Year-round | 1.9 | Low | 6 | Long | 279.30 | 129.24 | 5 | 270.40 | 103.31 | 5 | Guan etal. (2018) |
| MBC | mg /kg soil | 0-20 | Beiluhe | Alpine meadow | 306 | 6 | Herb | Moist | Year-round | >1.4 |  | 3 | Short | 335.03 | 99.73 | 3 | 719.32 | 184.65 | 3 | Zhang etal. (2015) |
| MBC | mg /kg soil | 0-10 | Damxung | Alpine meadow | 405 | 10.7 | Herb | Moist | Year-round | 1.6 | Low | 3 | Short | 114.00 | 26.83 | 5 | 99.60 | 83.18 | 5 | Zong etal. (2018) |
| MBC | mg /kg soil | 0-20 | Damxung | Alpine meadow | 405 | 10.7 | Herb | Moist | Year-round | 1.6 | Low | 4 | Long | 198.00 | 61.72 | 5 | 153.60 | 136.85 | 5 | Zong etal. (2018) |
| MBC | mg /kg soil | 0-20 | Damxung | Alpine meadow | 405 | 10.7 | Herb | Moist | Year-round | 1.6 | Low | 5 | Long | 204.00 | 88.55 | 5 | 169.20 | 53.67 | 5 | Zong etal. (2018) |
| MBC | mg /kg soil | 0-20 | Damxung | Alpine meadow | 405 | 10.7 | Herb | Moist | Year-round | 1-1.4 | Low | 5 | Long | 167.33 | 106.12 | 3 | 158.54 | 57.83 | 3 | Fu etal.(2013) |
| MBC | mg /kg soil | 0-20 | Damxung | Alpine meadow | 405 | 10.7 | Herb | Moist | Year-round | 1-1.4 | Low | 5 | Long | 207.39 | 43.23 | 3 | 251.62 | 67.53 | 3 | Fu etal.(2013) |
| MBC | mg /kg soil | 0-20 | Damxung | Alpine meadow | 405 | 10.7 | Herb | Moist | Year-round | 1-1.4 | Low | 5 | Long | 588.02 | 212.73 | 3 | 665.99 | 159.42 | 3 | Fu etal.(2013) |
| SOC | g/kg soil |  | Damxung | Alpine meadow | 405 | 10.7 | Herb | Moist | Year-round | 0.6-2.0 | Low | 2 | Short | 20.04 | 0.76 | 3 | 19.78 | 1.90 | 3 | Yu et al. (2014) |
| SOC | g/kg soil |  | Damxung | Alpine meadow | 405 | 10.7 | Herb | Moist | Year-round | 0.6-2.0 | Low | 2 | Short | 25.03 | 2.90 | 3 | 22.99 | 1.07 | 3 | Yu et al. (2014) |
| SOC | g/kg soil |  | Damxung | Alpine meadow | 405 | 10.7 | Herb | Moist | Year-round | 0.6-2.0 | Low | 2 | Short | 46.08 | 3.81 | 3 | 44.59 | 1.75 | 3 | Yu et al. (2014) |
| SOC | g/kg soil |  | Beiluhe | Alpine meadow | 276 | 6 | Herb | Moist | Year-round | 2.30 | High | 3 | Short | 7.03 | 1.12 | 5 | 6.84 | 1.22 | 5 | Xue et al. (2015) |
| SOC | g/kg soil |  | Nagqu | Alpine meadow | 370 | 8.5 | Herb | Moist | Year-round | 0.8-1.1 | Low | 2 | Short | 41.39 | 3.09 | 4 | 36.35 | 9.80 | 4 | Wang et al. (2014) |
| SOC | g/kg soil |  | Haibei | Alpine meadow | 370 | 8.5 | Herb | Moist | Year-round | 0.8-1.1 | Low | 10 | Long | 42.56 | 3.19 | 4 | 40.85 | 4.70 | 4 | Wang et al. (2014) |
| SOC | g/kg soil |  | Haibei | Alpine meadow | 370 | 8.5 | Herb | Moist | Growing-season | 1.2-1.7 | Low | 4 | Long | 0.57 | 0.15 | 4 | 0.56 | 0.11 | 4 | Rui et al. (2011) |
| SOC | g/kg soil |  | Nagqu | Alpine meadow | 345.6 | 12.4 | Herb | Moist | Year-round | 1.05 | Low | 4 | Long | 35.43 | 3.68 | 4 | 45.48 | 3.06 | 4 | Ganjurjav etal.(2016) |
| SOC | g/kg soil |  | Nagqu | Alpine meadow | 345.6 | 12.4 | Herb | Moist | Year-round | 1.69 | Low | 4 | Long | 35.43 | 3.68 | 4 | 51.21 | 11.36 | 4 | Ganjurjav etal.(2016) |
| SOC | g/kg soil |  | Dadu River | Alpine meadow | 360 | 17.4 | Herb | Moist | Growing-season | 1.63 | Low | 3 | Short | 125.10 | 132.15 | 3 | 98.60 | 106.00 | 3 | Xiong etal. (2016) |
| SOC | g/kg soil |  | Nagqu | Alpine meadow | 370 | 8.5 | Herb | Moist | Year-round | 2.70 | High | 4 | Long | 28.19 | 1.22 | 4 | 25.77 | 1.54 | 4 | Wang et al. (2018) |
| SOC | g/kg soil |  | Nagqu | Alpine meadow | 370 | 8.5 | Herb | Moist | Year-round | 2.70 | High | 4 | Long | 28.19 | 1.22 | 4 | 27.38 | 1.22 | 4 | Wang et al. (2018) |
| SOC | g/m^2^ |  | Fenghuoshan | Alpine meadow | 216 | 2.5 | Herb | Moist | Year-round | 2.59 | High | 2 | Short | 459.53 | 145.09 | 3 | 487.20 | 115.12 | 3 | Li etal. (2011) |
| SOC | g/m^2^ |  | Fenghuoshan | Alpine meadow | 216 | 2.5 | Herb | Moist | Year-round | 5.16 | High | 2 | Short | 459.53 | 145.09 | 3 | 370.90 | 91.85 | 3 | Li etal. (2011) |
| SOC | g/kg soil | 0-20 | Baingoin | Alpine steppe | 267.2 | 13 | Herb | Dry | Year-round | >2.25 |  | 4 | Long | 29.10 | 1.14 | 4 | 26.85 | 5.72 | 4 | Ganjurjav etal.(2016) |
| SOC | g/kg soil | 0-10 | Gangcha | Alpine steppe | 348 | 11.4 | Herb | Dry | Year-round | >2 | High | 3 | Short | 32.00 | 6.32 | 10 | 32.00 | 6.32 | 10 | Wang etal. (2018) |
| SOC | g/kg soil | 0-10 | Gangcha | Alpine steppe | 348 | 11.4 | Herb | Dry | Year-round | >2 | High | 4 | Long | 33.00 | 3.16 | 10 | 33.20 | 3.16 | 10 | Wang etal. (2018) |
| SOC | g/kg soil |  | Nam Co | Alpine steppe | 332 | 12 | Herb | Dry | Growing-season | 2 | Low | 1 | Short | 27.86 | 4.32 | 4 | 29.27 | 3.36 | 4 | Zhao etal.(2019) |
| SOC | g/kg soil |  | Nam Co | Alpine steppe | 332 | 12 | Herb | Dry | Growing-season | 2 | Low | 2 | Short | 27.49 | 1.90 | 4 | 28.67 | 1.42 | 4 | Zhao etal.(2019) |
| SOC | g/kg soil |  | Alexandra Fiord | Arctic tundra | 30 | 7.9 | Shrub | Dry | Year-round | 1–2 | Low | 16 | Long | 260.90 | 319.40 | 5 | 302.44 | 592.31 | 5 | Lamb etal. (2011) |
| SOC | g/kg soil |  | Fenghuoshan | Alpine swamp meadow | 216 | 1.42 | Herb | Dry | Year-round | 6.2 | High | 4 | Long | 66.11 | 15.92 | 4 | 71.17 | 10.26 | 4 | Chen etal.(2017) |
| SOC | g/kg soil |  | Fenghuoshan | Alpine swamp meadow | 216 | 1.42 | Herb | Wet | Year-round | 1.5-2.5 |  | 3 | Long | 91.78 | 27.50 | 3 | 72.89 | 7.93 | 3 | Xi etal.(2019) |
| SOC | g/kg soil |  | Fenghuoshan | Alpine swamp meadow | 216 | 1.42 | Herb | Wet | Year-round | 3-5 | High | 3 | Long | 91.78 | 27.50 | 3 | 57.39 | 5.57 | 3 | Xi etal.(2019) |
| SOC | g/m^2^ |  | Fenghuoshan | Alpine swamp meadow | 216 | 1.42 | Herb | Wet | Year-round | 2.98 | High | 2 | Short | 447.62 | 20.76 | 3 | 453.97 | 22.50 | 3 | Li etal. (2011) |
| SOC | g/m^2^ |  | Fenghuoshan | Alpine swamp meadow | 216 | 1.42 | Herb | Wet | Year-round | 5.52 | High | 2 | Short | 447.62 | 20.76 | 3 | 466.67 | 179.25 | 3 | Li etal. (2011) |
| SOC | g/kg soil | 0-20 | Damxung | Alpine meadow | 405 | 10.7 | Herb | Moist | Year-round | 1.9 | Low | 6 | Long | 17.84 | 4.25 | 5 | 17.98 | 1.96 | 5 | Guan etal. (2018) |
| SOC | g/kg soil |  | Damxung | Alpine meadow | 405 | 10.7 | Herb | Moist | Year-round |  |  | 8 | Long | 25.00 | 5.69 | 4 | 25.33 | 4.05 | 4 | Yu etal. (2019) |
| SOC | g/kg soil |  | Damxung | Alpine meadow | 405 | 10.7 | Herb | Moist | Year-round |  |  | 8 | Long | 26.38 | 2.40 | 4 | 29.98 | 3.23 | 4 | Yu etal. (2019) |
| SOC | g/kg soil |  | Damxung | Alpine meadow | 405 | 10.7 | Herb | Moist | Year-round |  |  | 8 | Long | 67.20 | 10.62 | 4 | 72.86 | 9.82 | 4 | Yu etal. (2019) |
| SOC | g/kg soil |  | Beiluhe | Alpine swamp meadow | 306 | 6 | Herb | Wet | Year-round | >1.7 | High | 3 | Short | 22.40 | 7.40 | 3 | 29.10 | 10.76 | 3 | Zhang etal. (2014) |
| SOC | g/kg soil |  | Beiluhe | Alpine meadow | 306 | 6 | Herb | Moist | Year-round | >2.3 | High | 3 | Short | 16.50 | 1.32 | 3 | 17.10 | 3.26 | 3 | Zhang etal. (2014) |
| SOC | g/kg soil |  | Beiluhe | Alpine steppe | 306 | 6 | Herb+Shrub | Dry | Year-round | >1.7 | High | 3 | Short | 4.97 | 0.35 | 3 | 4.77 | 0.80 | 3 | Zhang etal. (2014) |
| SOC | g/kg soil |  | Suli | Alpine meadow | 370 | 12.30 | Herb | Moist | Growing-season | 2.18 | High | 1 | Short | 14.89 | 2.49 | 3 | 16.59 | 3.71 | 3 | Yu et al. (2015) |
| SOC | g/kg soil |  | Damxung | Alpine meadow | 405 | 10.7 | Herb | Moist | Year-round | 1-1.4 | Low | 5 | Long | 22.41 | 4.66 | 3 | 23.31 | 3.11 | 3 | Fu etal.(2013) |
| SOC | g/kg soil |  | Damxung | Alpine meadow | 405 | 10.7 | Herb | Moist | Year-round | 1-1.4 | Low | 5 | Long | 26.67 | 3.49 | 3 | 28.24 | 3.11 | 3 | Fu etal.(2013) |
| SOC | g/kg soil |  | Damxung | Alpine meadow | 405 | 10.7 | Herb | Moist | Year-round | 1-1.4 | Low | 5 | Long | 54.90 | 6.99 | 3 | 51.77 | 10.09 | 3 | Fu etal.(2013) |
| NEE | g-C/m^2^/d |  | Nagqu | Alpine steppe | 348 | 11.4 | Herb | Dry | Year-round | 1.7-2.0 | Low | 2 | Short | 4.52 | 3.15 | 4 | 2.88 | 0.27 | 4 | Li etal.(2019) |
| NEE | g-C/m^2^/d |  | Nagqu | Alpine steppe | 348 | 11.4 | Herb | Dry | Year-round | 1.7-2.0 | Low | 3 | Short | 2.44 | 0.51 | 4 | 0.96 | 0.34 | 4 | Li etal.(2019) |
| NEE | g-C/m^2^/d |  | Nagqu | Alpine steppe | 348 | 11.4 | Herb | Dry | Year-round | 1.7-2.0 | Low | 4 | Long | 3.40 | 1.02 | 4 | 1.24 | 0.21 | 4 | Li etal.(2019) |
| NEE | g-C/m^2^/d |  | Eight Mile Lake | Arctic tundra | 235 | 13.5 | Herb+Shrub | Moist | Growing-season | 1 | Low | 1 | Short | 0.08 | 0.16 | 6 | 0.13 | 0.24 | 6 | Natali etal. (2014) |
| NEE | g-C/m^2^/d |  | N-Siberia | Arctic tundra | 310 | 11 | Shrub | Dry | Year-round | 3.60 | High | 2 | Short | -0.05 | 0.04 | 9 | -0.39 | 0.16 | 9 | Biasi etal. (2006) |
| NEE | g-C/m^2^/d |  | Alexandra Fiord | Arctic tundra | 30 | 8.7 | Shrub | Dry | Year-round | 1 | Low | 9 | Long | 0.43 | 0.06 | 3 | 0.49 | 0.08 | 3 | Welker etal. (2004) |
| NEE | g-C/m^2^/d |  | Alexandra Fiord | Arctic tundra | 30 | 8.1 | Herb | Wet | Year-round | 0.2 | Low | 9 | Long | 0.95 | 0.10 | 3 | 0.77 | 0.08 | 3 | Welker etal. (2004) |
| NEE | g-C/m^2^/d |  | Alexandra Fiord | Arctic tundra | 30 | 8.7 | Shrub | Dry | Growing-season | 1-2 | Low | 9 | Long | 0.13 | 0.09 | 3 | 0.53 | 0.32 | 3 | Oberbauer etal. (2007) |
| NEE | g-C/m^2^/d |  | Alexandra Fiord | Arctic tundra | 30 | 8.7 | Shrub | Dry | Growing-season | 1-2 | Low | 10 | Long | 0.51 | 0.35 | 3 | 0.29 | 0.37 | 3 | Oberbauer etal. (2007) |
| NEE | g-C/m^2^/d |  | Alexandra Fiord | Arctic tundra | 30 | 7.9 | Shrub | Moist | Growing-season | 1-2 | Low | 9 | Long | -0.49 | 0.32 | 3 | -0.36 | 0.50 | 3 | Oberbauer etal. (2007) |
| NEE | g-C/m^2^/d |  | Alexandra Fiord | Arctic tundra | 30 | 7.9 | Shrub | Moist | Growing-season | 1-2 | Low | 10 | Long | -0.56 | 0.43 | 3 | -0.37 | 0.51 | 3 | Oberbauer etal. (2007) |
| NEE | g-C/m^2^/d |  | Alexandra Fiord | Arctic tundra | 30 | 8.1 | Herb | Wet | Growing-season | 1-2 | Low | 9 | Long | 0.48 | 0.39 | 3 | 0.49 | 0.57 | 3 | Oberbauer etal. (2007) |
| NEE | g-C/m^2^/d |  | Alexandra Fiord | Arctic tundra | 30 | 8.1 | Herb | Wet | Growing-season | 1-2 | Low | 10 | Long | 0.79 | 0.57 | 3 | 0.52 | 0.53 | 3 | Oberbauer etal. (2007) |
| NEE | g-C/m^2^/d |  | Barrow | Arctic tundra | 57 | 3.7 | Shrub | Dry | Year-round | 1-2 | Low | 7 | Long | -0.02 | 0.16 | 5 | -1.11 | 0.62 | 5 | Oberbauer etal. (2007) |
| NEE | g-C/m^2^/d |  | Barrow | Arctic tundra | 57 | 3.7 | Shrub | Dry | Year-round | 1-2 | Low | 8 | Long | 0.04 | 0.07 | 5 | -0.45 | 0.37 | 5 | Oberbauer etal. (2007) |
| NEE | g-C/m^2^/d |  | Barrow | Arctic tundra | 57 | 3.7 | Herb | Wet | Year-round | 1-2 | Low | 6 | Long | 0.23 | 0.09 | 5 | 0.27 | 0.16 | 5 | Oberbauer etal. (2007) |
| NEE | g-C/m^2^/d |  | Barrow | Arctic tundra | 57 | 3.7 | Herb | Wet | Year-round | 1-2 | Low | 7 | Long | 0.52 | 0.14 | 5 | 0.81 | 0.23 | 5 | Oberbauer etal. (2007) |
| NEE | g-C/m^2^/d |  | Atqasuk | Arctic tundra | 55 | 9 | Shrub | Dry | Year-round | 1-2 | Low | 5 | Long | -0.18 | 0.11 | 5 | -0.30 | 0.16 | 5 | Oberbauer etal. (2007) |
| NEE | g-C/m^2^/d |  | Atqasuk | Arctic tundra | 55 | 9 | Herb | Wet | Year-round | 1-2 | Low | 5 | Long | 0.46 | 0.16 | 5 | 0.75 | 0.30 | 5 | Oberbauer etal. (2007) |
| NEE | g-C/m^2^/d |  | Atqasuk | Arctic tundra | 55 | 9 | Herb | Wet | Year-round | 1-2 | Low | 6 | Long | 0.55 | 0.14 | 5 | 0.71 | 0.14 | 5 | Oberbauer etal. (2007) |
| NEE | g-C/m^2^/d |  | Toolik Lake | Arctic tundra | 180 | 10.00 | Shrub | Dry | Year-round | 1-2 | Low | 3 | Short | -0.50 | 0.48 | 3 | -1.79 | 0.59 | 3 | Oberbauer etal. (2007) |
| NEE | g-C/m^2^/d |  | Toolik Lake | Arctic tundra | 180 | 10.00 | Shrub | Dry | Year-round | 1-2 | Low | 4 | Long | 0.12 | 0.37 | 3 | -0.11 | 0.21 | 3 | Oberbauer etal. (2007) |
| NEE | g-C/m^2^/d |  | Toolik Lake | Arctic tundra | 180 | 10.00 | Herb | Moist | Year-round | 1-2 | Low | 3 | Short | -0.43 | 0.18 | 3 | -0.66 | 0.62 | 3 | Oberbauer etal. (2007) |
| NEE | g-C/m^2^/d |  | Toolik Lake | Arctic tundra | 180 | 10.00 | Herb | Moist | Year-round | 1-2 | Low | 4 | Long | 0.12 | 0.25 | 3 | -0.57 | 0.46 | 3 | Oberbauer etal. (2007) |
| NEE | g-C/m^2^/d |  | Seida | Arctic tundra | 450 | 12.5 | Moss | Wet | Growing-season | 0.95 | Low | 2 | Short | -1.16 | 0.40 | 5 | -2.31 | 0.40 | 5 | Voigt etal. (2017) |
| NEE | g-C/m^2^/d |  | Seida | Arctic tundra | 450 | 13.4 | Shrub | Wet | Growing-season | 0.95 | Low | 2 | Short | 1.10 | 1.39 | 5 | -0.56 | 3.18 | 5 | Voigt etal. (2017) |
| NEE | g-C/m^2^/d |  | Seida | Arctic tundra | 450 | 12.5 | Shrub | Dry | Growing-season | 0.95 | Low | 2 | Short | -0.53 | 1.85 | 5 | -4.03 | 2.45 | 5 | Voigt etal. (2017) |
| NEE | g-C/m^2^/d |  | Seida | Arctic tundra | 450 | 12.5 | Moss | Wet | Growing-season | 0.95 | Low | 3 | Short | -1.43 | 0.23 | 5 | -2.06 | 0.17 | 5 | Voigt etal. (2017) |
| NEE | g-C/m^2^/d |  | Seida | Arctic tundra | 450 | 13.4 | Shrub | Wet | Growing-season | 0.95 | Low | 3 | Short | 1.63 | 0.97 | 5 | -0.79 | 1.88 | 5 | Voigt etal. (2017) |
| NEE | g-C/m^2^/d |  | Seida | Arctic tundra | 450 | 12.5 | Shrub | Dry | Growing-season | 0.95 | Low | 3 | Short | 1.15 | 0.74 | 5 | -0.59 | 1.65 | 5 | Voigt etal. (2017) |
| NEE | g-C/m^2^/d |  | Toolik Lake | Arctic tundra | 180 | 10.00 | Moss | Wet | Growing-season | 2-5 | High | 11 | Long | 1.84 | 2.56 | 5 | 3.24 | 3.07 | 5 | Leffler etal. (2016) |
| NEE | g-C/m^2^/d |  | Eight Mile Lake | Arctic tundra | 235 | 13.5 | Herb+Shrub | Moist | Growing-season | 2.20 | High | 1 | Short | 0.05 | 0.40 | 5 | 0.08 | 0.50 | 5 | Natali etal. (2011) |
| NEE | g-C/m^2^/d |  | Toolik Lake | Arctic tundra | 180 | 10.00 | Herb | Wet | Growing-season |  |  | 14 | Long | 2.33 | 0.43 | 5 | 3.89 | 0.87 | 5 | Boelman etal. (2003) |
| NEE | g-C/m^2^/d |  | Abisko | Subarctic heath | 120.00 | 12.70 | Shrub | Dry | Growing-season | 2.40 | High | 10 | Long | -0.22 | 0.53 | 6 | 0.09 | 0.58 | 6 | Illeris etal.(2004) |
| NEE | g-C/m^2^/d |  | Abisko | Subarctic heath | 120.00 | 11.90 | Shrub | Wet | Growing-season | 1 | Low | 16 | Long | 1.82 | 0.43 | 6 | 1.44 | 0.43 | 6 | Pedersen etal.(2017) |
| NEE | g-C/m^2^/d |  | Gangcha | Alpine swamp meadow | 315.00 | 11 | Herb | Wet | Year-round | >1.6 | High | 3 | Short | 13.26 | 8.67 | 16 | 11.56 | 8.97 | 16 | Li etal.(2017) |
| NEE | g-C/m^2^/d |  | Gangcha | Alpine swamp meadow | 315.00 | 11 | Herb | Wet | Year-round | >1.6 | High | 4 | Long | 6.56 | 7.35 | 16 | 6.44 | 7.07 | 16 | Li etal.(2017) |
| NEE | g-C/m^2^/d |  | Nagqu | Alpine meadow | 370 | 8.5 | Herb | Moist | Year-round | 2.20 | High | 1 | Short | 3.51 | 1.06 | 2 | 3.62 | 3.61 | 2 | Zhu etal. (2017) |
| NEE | g-C/m^2^/d |  | Nagqu | Alpine meadow | 370 | 8.5 | Herb | Moist | Year-round | 2.20 | High | 2 | Short | 2.39 | 0.26 | 2 | 2.17 | 1.16 | 2 | Zhu etal. (2017) |
| GEP | g-C/m^2^/d |  | Damxung | Alpine meadow | 405 | 10.7 | Herb | Moist | Year-round | 1.08 | Low | 3 | Short | 1.13 | 0.10 | 3 | 0.81 | 0.10 | 3 | Fu et al. (2015) |
| GEP | g-C/m^2^/d |  | Damxung | Alpine meadow | 405 | 10.7 | Herb | Moist | Year-round | 1.81 | Low | 3 | Short | 1.89 | 0.16 | 3 | 1.61 | 0.18 | 3 | Fu et al. (2015) |
| GEP | g-C/m^2^/d |  | Beiluhe | Alpine meadow | 276.00 | 6 | Herb | Moist | Year-round | 1.88 | Low | 3-4 | Long | 5.16 | 2.63 | 5 | 6.73 | 2.56 | 5 | Peng et al. (2014) |
| GEP | g-C/m^2^/d |  | Gonghe | Alpine steppe | 348 | 11.4 | Herb | Dry | Year-round | >1 | High | 2 | Short | 11.11 | 12.15 | 19 | 5.15 | 5.80 | 19 | Li etal.(2019) |
| GEP | g-C/m^2^/d |  | Gonghe | Alpine steppe | 348 | 11.4 | Herb | Dry | Year-round | >1 | High | 3 | Short | 9.97 | 4.09 | 19 | 10.22 | 3.53 | 19 | Li etal.(2019) |
| GEP | g-C/m^2^/d |  | Gonghe | Alpine steppe | 348 | 11.4 | Herb | Dry | Year-round | >1 | High | 4 | Long | 9.01 | 3.81 | 15 | 10.41 | 4.75 | 15 | Li etal.(2019) |
| GEP | g-C/m^2^/d |  | Nagqu | Alpine steppe | 348 | 11.4 | Herb | Dry | Year-round | 1.7-2.0 | Low | 2 | Short | 7.88 | 4.22 | 4 | 5.80 | 0.73 | 4 | Li etal.(2019) |
| GEP | g-C/m^2^/d |  | Nagqu | Alpine steppe | 348 | 11.4 | Herb | Dry | Year-round | 1.7-2.0 | Low | 3 | Short | 4.57 | 1.21 | 4 | 2.78 | 0.38 | 4 | Li etal.(2019) |
| GEP | g-C/m^2^/d |  | Nagqu | Alpine steppe | 348 | 11.4 | Herb | Dry | Year-round | 1.7-2.0 | Low | 4 | Long | 5.88 | 1.09 | 4 | 3.19 | 0.85 | 4 | Li etal.(2019) |
| GEP | g-C/m^2^/d |  | Eight Mile Lake | Arctic tundra | 235 | 13.5 | Herb+Shrub | Moist | Growing-season | 1 | Low | 1 | Short | 2.18 | 0.33 | 6 | 2.43 | 0.24 | 6 | Natali etal. (2014) |
| GEP | g-C/m^2^/d |  | Eight Mile Lake | Arctic tundra | 235 | 13.5 | Herb+Shrub | Moist | Growing-season | 1 | Low | 2 | Short | 2.42 | 0.43 | 6 | 2.90 | 0.33 | 6 | Natali etal. (2014) |
| GEP | g-C/m^2^/d |  | Eight Mile Lake | Arctic tundra | 235 | 13.5 | Herb+Shrub | Moist | Growing-season | 1 | Low | 3 | Short | 2.36 | 0.51 | 6 | 2.97 | 0.39 | 6 | Natali etal. (2014) |
| GEP | g-C/m^2^/d |  | Toolik Lake | Arctic tundra | 180 | 10.00 | Shrub | Dry | Year-round | 2–3 | High | 2 | Short | 1.12 | 0.90 | 5 | 1.06 | 0.75 | 5 | Welker etal. (1999) |
| GEP | g-C/m^2^/d |  | Toolik Lake | Alpine tundra | 180 | 10.00 | Shrub | Dry | Year-round | 2–3 | High | 2 | Short | 1.53 | 1.14 | 4 | 1.54 | 0.90 | 4 | Welker etal. (1999) |
| GEP | g-C/m^2^/d |  | N-Siberia | Arctic tundra | 310 | 11 | Shrub | Dry | Year-round | 3.60 | High | 2 | Short | 0.11 | 0.14 | 9 | 0.31 | 0.26 | 9 | Biasi etal. (2006) |
| GEP | g-C/m^2^/d |  | Alexandra Fiord | Arctic tundra | 30 | 8.7 | Shrub | Dry | Year-round | 0.2 | Low | 9 | Long | 5.81 | 0.41 | 3 | 6.88 | 0.40 | 3 | Welker etal. (2004) |
| GEP | g-C/m^2^/d |  | Alexandra Fiord | Arctic tundra | 30 | 7.9 | Shrub | Moist | Year-round | 1 | Low | 9 | Long | 2.91 | 0.30 | 3 | 3.71 | 0.41 | 3 | Welker etal. (2004) |
| GEP | g-C/m^2^/d |  | Alexandra Fiord | Arctic tundra | 30 | 8.1 | Herb | Wet | Year-round | 0.2 | Low | 9 | Long | 3.76 | 0.33 | 3 | 3.71 | 0.46 | 3 | Welker etal. (2004) |
| GEP | g-C/m^2^/d |  | Alexandra Fiord | Arctic tundra | 30 | 8.7 | Shrub | Dry | Growing-season | 1-2 | Low | 9 | Long | 2.79 | 0.73 | 3 | 3.17 | 0.79 | 3 | Oberbauer etal. (2007) |
| GEP | g-C/m^2^/d |  | Alexandra Fiord | Arctic tundra | 30 | 8.7 | Shrub | Dry | Growing-season | 1-2 | Low | 10 | Long | 3.54 | 1.06 | 3 | 4.26 | 1.30 | 3 | Oberbauer etal. (2007) |
| GEP | g-C/m^2^/d |  | Alexandra Fiord | Arctic tundra | 30 | 7.9 | Shrub | Moist | Growing-season | 1-2 | Low | 9 | Long | 0.94 | 1.00 | 3 | 1.79 | 1.14 | 3 | Oberbauer etal. (2007) |
| GEP | g-C/m^2^/d |  | Alexandra Fiord | Arctic tundra | 30 | 7.9 | Shrub | Moist | Growing-season | 1-2 | Low | 10 | Long | 1.27 | 0.68 | 3 | 1.63 | 0.87 | 3 | Oberbauer etal. (2007) |
| GEP | g-C/m^2^/d |  | Alexandra Fiord | Arctic tundra | 30 | 8.1 | Herb | Wet | Growing-season | 1-2 | Low | 9 | Long | 2.17 | 0.76 | 3 | 2.17 | 0.43 | 3 | Oberbauer etal. (2007) |
| GEP | g-C/m^2^/d |  | Alexandra Fiord | Arctic tundra | 30 | 8.1 | Herb | Wet | Growing-season | 1-2 | Low | 10 | Long | 2.10 | 0.76 | 3 | 2.12 | 0.79 | 3 | Oberbauer etal. (2007) |
| GEP | g-C/m^2^/d |  | Barrow | Arctic tundra | 57 | 3.7 | Shrub | Dry | Year-round | 1-2 | Low | 7 | Long | 0.87 | 0.21 | 5 | 0.96 | 0.49 | 5 | Oberbauer etal. (2007) |
| GEP | g-C/m^2^/d |  | Barrow | Arctic tundra | 57 | 3.7 | Shrub | Dry | Year-round | 1-2 | Low | 8 | Long | 0.96 | 0.21 | 5 | 1.23 | 0.46 | 5 | Oberbauer etal. (2007) |
| GEP | g-C/m^2^/d |  | Barrow | Arctic tundra | 57 | 3.7 | Herb | Wet | Year-round | 1-2 | Low | 6 | Long | 1.56 | 0.42 | 5 | 1.79 | 0.39 | 5 | Oberbauer etal. (2007) |
| GEP | g-C/m^2^/d |  | Barrow | Arctic tundra | 57 | 3.7 | Herb | Wet | Year-round | 1-2 | Low | 7 | Long | 2.08 | 0.32 | 5 | 2.45 | 0.53 | 5 | Oberbauer etal. (2007) |
| GEP | g-C/m^2^/d |  | Atqasuk | Arctic tundra | 55 | 9 | Shrub | Dry | Year-round | 1-2 | Low | 5 | Long | 0.60 | 0.25 | 5 | 0.81 | 0.11 | 5 | Oberbauer etal. (2007) |
| GEP | g-C/m^2^/d |  | Atqasuk | Arctic tundra | 55 | 9 | Shrub | Dry | Year-round | 1-2 | Low | 6 | Long | 0.53 | 0.25 | 5 | 0.78 | 0.07 | 5 | Oberbauer etal. (2007) |
| GEP | g-C/m^2^/d |  | Atqasuk | Arctic tundra | 55 | 9 | Herb | Wet | Year-round | 1-2 | Low | 5 | Long | 1.20 | 0.32 | 5 | 1.39 | 0.28 | 5 | Oberbauer etal. (2007) |
| GEP | g-C/m^2^/d |  | Atqasuk | Arctic tundra | 55 | 9 | Herb | Wet | Year-round | 1-2 | Low | 6 | Long | 1.69 | 0.28 | 5 | 1.58 | 0.39 | 5 | Oberbauer etal. (2007) |
| GEP | g-C/m^2^/d |  | Toolik Lake | Arctic tundra | 180 | 10.00 | Shrub | Dry | Year-round | 1-2 | Low | 3 | Short | 1.22 | 0.54 | 3 | 1.24 | 0.65 | 3 | Oberbauer etal. (2007) |
| GEP | g-C/m^2^/d |  | Toolik Lake | Arctic tundra | 180 | 10.00 | Shrub | Dry | Year-round | 1-2 | Low | 4 | Long | 1.66 | 0.38 | 3 | 2.10 | 0.65 | 3 | Oberbauer etal. (2007) |
| GEP | g-C/m^2^/d |  | Toolik Lake | Arctic tundra | 180 | 10.00 | Herb | Moist | Year-round | 1-2 | Low | 3 | Short | 1.78 | 0.79 | 3 | 1.19 | 0.57 | 3 | Oberbauer etal. (2007) |
| GEP | g-C/m^2^/d |  | Toolik Lake | Arctic tundra | 180 | 10.00 | Herb | Moist | Year-round | 1-2 | Low | 4 | Long | 3.21 | 1.22 | 3 | 2.40 | 1.33 | 3 | Oberbauer etal. (2007) |
| GEP | g-C/m^2^/d |  | Seida | Arctic tundra | 450 | 12.5 | Moss | Wet | Growing-season | 0.95 | Low | 2 | Short | 0.38 | 0.17 | 5 | 0.23 | 0.68 | 5 | Voigt etal. (2017) |
| GEP | g-C/m^2^/d |  | Seida | Arctic tundra | 450 | 13.4 | Shrub | Wet | Growing-season | 0.95 | Low | 2 | Short | 6.35 | 0.85 | 5 | 5.75 | 1.35 | 5 | Voigt etal. (2017) |
| GEP | g-C/m^2^/d |  | Seida | Arctic tundra | 450 | 12.5 | Shrub | Dry | Growing-season | 0.95 | Low | 2 | Short | 6.66 | 0.85 | 5 | 6.58 | 1.18 | 5 | Voigt etal. (2017) |
| GEP | g-C/m^2^/d |  | Seida | Arctic tundra | 450 | 12.5 | Moss | Wet | Growing-season | 0.95 | Low | 3 | Short | 0.26 | 0.44 | 5 | 0.33 | 0.44 | 5 | Voigt etal. (2017) |
| GEP | g-C/m^2^/d |  | Seida | Arctic tundra | 450 | 13.4 | Shrub | Wet | Growing-season | 0.95 | Low | 3 | Short | 7.35 | 1.60 | 5 | 5.98 | 1.16 | 5 | Voigt etal. (2017) |
| GEP | g-C/m^2^/d |  | Seida | Arctic tundra | 450 | 12.5 | Shrub | Dry | Growing-season | 0.95 | Low | 3 | Short | 6.63 | 0.87 | 5 | 5.98 | 1.02 | 5 | Voigt etal. (2017) |
| GEP | g-C/m^2^/d |  | Toolik Lake | Arctic tundra | 180 | 10.00 | Moss | Wet | Growing-season | 2-5 | High | 11 | Long | 5.44 | 2.25 | 5 | 6.02 | 3.07 | 5 | Leffler etal. (2016) |
| GEP | g-C/m^2^/d |  | Eight Mile Lake | Arctic tundra | 235 | 13.5 | Herb | Moist | Growing-season | 2.20 | High | 1 | Short | 1.35 | 0.61 | 5 | 1.63 | 0.75 | 5 | Natali etal. (2011) |
| GEP | g-C/m^2^/d |  | Toolik Lake | Arctic tundra | 180 | 10.00 | Shrub | Wet | Growing-season |  |  | 14 | Long | 5.83 | 0.87 | 5 | 7.45 | 1.16 | 5 | Boelman etal. (2003) |
| GEP | g-C/m^2^/d |  | Abisko | Subarctic heath | 120.00 | 12.70 | Shrub | Dry | Growing-season | 2.40 | High | 10 | Long | 1.74 | 0.79 | 6 | 2.31 | 0.59 | 6 | Illeris etal.(2004) |
| GEP | g-C/m^2^/d |  | Abisko | Subarctic heath | 120.00 | 11.90 | Herb | Wet | Growing-season | 1 | Low | 16 | Long | 4.28 | 0.41 | 6 | 5.21 | 0.52 | 6 | Pedersen etal.(2017) |
| GEP | g-C/m^2^/d |  | Gangcha | Alpine swamp meadow | 315.00 | 11 | Herb | Wet | Year-round | >1.6 | High | 2 | Short | 13.52 | 9.05 | 16 | 13.95 | 9.19 | 16 | Li etal.(2017) |
| GEP | g-C/m^2^/d |  | Gangcha | Alpine swamp meadow | 315.00 | 11 | Herb | Wet | Year-round | >1.6 | High | 3 | Short | 13.26 | 8.67 | 16 | 11.56 | 8.97 | 16 | Li etal.(2017) |
| GEP | g-C/m^2^/d |  | Gangcha | Alpine swamp meadow | 315.00 | 11 | Herb | Wet | Year-round | >1.6 | High | 4 | Long | 13.05 | 9.79 | 16 | 13.37 | 9.16 | 16 | Li etal.(2017) |
| GEP | g-C/m^2^/d |  | Nagqu | Alpine meadow | 370 | 8.5 | Herb | Moist | Year-round | 2.20 | High | 1 | Short | 7.04 | 1.49 | 2 | 6.90 | 5.06 | 2 | Zhu etal. (2017) |
| GEP | g-C/m^2^/d |  | Nagqu | Alpine meadow | 370 | 8.5 | Herb | Moist | Year-round | 2.20 | High | 2 | Short | 4.33 | 0.17 | 2 | 4.86 | 1.32 | 2 | Zhu etal. (2017) |
| GEP | g-C/m^2^/d |  | Damxung | Alpine meadow | 405 | 10.7 | Herb | Moist | Year-round | 1.91 | Low | 1 | Short | 0.57 | 0.38 | 4 | 0.61 | 0.35 | 4 | Fu etal. (2017) |
| GEP | g-C/m^2^/d |  | Damxung | Alpine meadow | 405 | 10.7 | Herb | Moist | Year-round | 3.51 | High | 1 | Short | 0.57 | 0.38 | 4 | 0.66 | 0.46 | 4 | Fu etal. (2017) |
| GEP | g-C/m^2^/d |  | Damxung | Alpine meadow | 405 | 10.7 | Herb | Moist | Year-round | 1.91 | Low | 2 | Short | 0.35 | 0.25 | 4 | 0.35 | 0.31 | 4 | Fu etal. (2017) |
| GEP | g-C/m^2^/d |  | Damxung | Alpine meadow | 405 | 10.7 | Herb | Moist | Year-round | 3.51 | High | 2 | Short | 0.35 | 0.25 | 4 | 0.35 | 0.37 | 4 | Fu etal. (2017) |
| GEP | g-C/m^2^/d |  | Damxung | Alpine meadow | 405 | 10.7 | Herb | Moist | Year-round | 1.91 | Low | 3 | Short | 0.70 | 0.31 | 4 | 0.63 | 0.35 | 4 | Fu etal. (2017) |
| GEP | g-C/m^2^/d |  | Damxung | Alpine meadow | 405 | 10.7 | Herb | Moist | Year-round | 3.51 | High | 3 | Short | 0.70 | 0.31 | 4 | 0.70 | 0.39 | 4 | Fu etal. (2017) |
| GEP | g-C/m^2^/d |  | Damxung | Alpine meadow | 405 | 10.7 | Herb | Moist | Growing-season | 1.91 | Low | 1 | Short | 0.38 | 0.29 | 4 | 0.39 | 0.22 | 4 | Fu etal. (2019) |
| GEP | g-C/m^2^/d |  | Damxung | Alpine meadow | 405 | 10.7 | Herb | Moist | Growing-season | 1.91 | Low | 3 | Short | 0.62 | 0.33 | 4 | 0.85 | 0.54 | 4 | Fu etal. (2019) |
| ER | g-C/m^2^/d |  | Suli | Alpine meadow | 370 | 12.30 | Herb | Moist | Year-round | 2.00 | Low | 1 | Short | 2.18 | 1.00 | 16 | 2.66 | 1.27 | 16 | Yu et al. (2015) |
| ER | g-C/m^2^/d |  | Kakagou | Alpine meadow | 574.4 | 7.33 | Herb | Moist | Year-round | >1.8 | High | 1 | Short | 5.83 | 1.20 | 6 | 7.76 | 1.01 | 6 | Shi et al. (2012) |
| ER | g-C/m^2^/d |  | Haibei | Alpine meadow | 370 | 8.5 | Herb | Moist | Growing-season | 1.2-1.7 | Low | 2 | Short | 3.45 | 1.48 | 6 | 2.40 | 1.75 | 6 | Lin et al. (2011) |
| ER | g-C/m^2^/d |  | Haibei | Alpine meadow | 370 | 8.5 | Herb | Moist | Growing-season | 1.2-1.7 | Low | 3 | Short | 2.96 | 1.51 | 12 | 3.03 | 1.28 | 12 | Lin et al. (2011) |
| ER | g-C/m^2^/d |  | Damxung | Alpine meadow | 405 | 10.7 | Herb | Moist | Year-round | 1.00 | Low | 1 | Short | 0.81 | 0.72 | 12 | 1.27 | 1.06 | 12 | Geng et al. (2017) |
| ER | g-C/m^2^/d |  | Damxung | Alpine meadow | 405 | 10.7 | Herb | Moist | Year-round | 2.00 | Low | 1 | Short | 0.81 | 0.72 | 12 | 1.11 | 0.58 | 12 | Geng et al. (2017) |
| ER | g-C/m^2^/d |  | Damxung | Alpine meadow | 405 | 10.7 | Herb | Moist | Year-round | 3.00 | High | 1 | Short | 0.81 | 0.72 | 12 | 0.98 | 0.58 | 12 | Geng et al. (2017) |
| ER | g-C/m^2^/d |  | Damxung | Alpine meadow | 405 | 10.7 | Herb | Moist | Year-round | 4.00 | High | 1 | Short | 0.81 | 0.72 | 12 | 0.78 | 0.48 | 12 | Geng et al. (2017) |
| ER | g-C/m^2^/d |  | Damxung | Alpine meadow | 405 | 10.7 | Herb | Moist | Year-round | 1.00 | Low | 2 | Short | 1.90 | 1.83 | 12 | 2.16 | 2.02 | 12 | Geng et al. (2017) |
| ER | g-C/m^2^/d |  | Damxung | Alpine meadow | 405 | 10.7 | Herb | Moist | Year-round | 2.00 | Low | 2 | Short | 1.90 | 1.83 | 12 | 2.04 | 1.59 | 12 | Geng et al. (2017) |
| ER | g-C/m^2^/d |  | Damxung | Alpine meadow | 405 | 10.7 | Herb | Moist | Year-round | 3.00 | High | 2 | Short | 1.90 | 1.83 | 12 | 1.80 | 1.54 | 12 | Geng et al. (2017) |
| ER | g-C/m^2^/d |  | Damxung | Alpine meadow | 405 | 10.7 | Herb | Moist | Year-round | 4.00 | High | 2 | Short | 1.90 | 1.83 | 12 | 1.55 | 1.35 | 12 | Geng et al. (2017) |
| ER | g-C/m^2^/d |  | Damxung | Alpine meadow | 405 | 10.7 | Herb | Moist | Year-round | 1.00 | Low | 3 | Short | 0.32 | 0.14 | 12 | 0.85 | 0.15 | 12 | Geng et al. (2017) |
| ER | g-C/m^2^/d |  | Damxung | Alpine meadow | 405 | 10.7 | Herb | Moist | Year-round | 2.00 | Low | 3 | Short | 0.32 | 0.14 | 12 | 2.00 | 2.56 | 12 | Geng et al. (2017) |
| ER | g-C/m^2^/d |  | Damxung | Alpine meadow | 405 | 10.7 | Herb | Moist | Year-round | 3.00 | High | 3 | Short | 0.32 | 0.14 | 12 | 1.03 | 1.45 | 12 | Geng et al. (2017) |
| ER | g-C/m^2^/d |  | Damxung | Alpine meadow | 405 | 10.7 | Herb | Moist | Year-round | 4.00 | High | 3 | Short | 0.32 | 0.14 | 12 | 1.50 | 0.10 | 12 | Geng et al. (2017) |
| ER | g-C/m^2^/d |  | Beiluhe | Alpine meadow | 276.00 | 6 | Herb | Moist | Year-round | 1.88 | Low | 3-4 | Long | 2.83 | 1.61 | 5 | 3.62 | 1.64 | 5 | Peng et al. (2014) |
| ER | g-C/m^2^/d |  | Haiyan | Alpine meadow | 280 | 10.5 | Herb | Moist | Year-round | 2 | Low | 1 | Short | 3.50 | 2.37 | 3 | 3.52 | 0.43 | 3 | Zhao etal.(2017) |
| ER | g-C/m^2^/d |  | Gonghe | Alpine steppe | 302.00 | 11.10 | Herb | Dry | Year-round | 2 | Low | 1 | Short | 4.61 | 1.56 | 3 | 2.72 | 0.54 | 3 | Zhao etal.(2017) |
| ER | g-C/m^2^/d |  | Nam Co | Alpine steppe | 332 | 12 | Herb | Dry | Growing-season | 2 | Low | 1 | Short | 2.38 | 0.06 | 4 | 1.73 | 0.38 | 4 | Zhao etal.(2019) |
| ER | g-C/m^2^/d |  | Nam Co | Alpine steppe | 332 | 12 | Herb | Dry | Growing-season | 2 | Low | 2 | Short | 3.20 | 0.38 | 4 | 2.15 | 0.38 | 4 | Zhao etal.(2019) |
| ER | g-C/m^2^/d |  | Nagqu | Alpine steppe | 348 | 11.4 | Herb | Dry | Year-round | 1.7-2.0 | Low | 2 | Short | 3.45 | 1.15 | 4 | 3.00 | 0.66 | 4 | Li etal.(2019) |
| ER | g-C/m^2^/d |  | Nagqu | Alpine steppe | 348 | 11.4 | Herb | Dry | Year-round | 1.7-2.0 | Low | 3 | Short | 2.24 | 0.22 | 4 | 1.70 | 0.22 | 4 | Li etal.(2019) |
| ER | g-C/m^2^/d |  | Nagqu | Alpine steppe | 348 | 11.4 | Herb | Dry | Year-round | 1.7-2.0 | Low | 4 | Long | 2.42 | 0.51 | 4 | 1.87 | 0.57 | 4 | Li etal.(2019) |
| ER | g-C/m^2^/d |  | Alexandra Fiord | Arctic tundra | 30 | 7.9 | Shrub | Dry | Year-round | 1–2 | Low | 16 | Long | 1.34 | 1.81 | 5 | 0.83 | 1.10 | 5 | Lamb etal. (2011) |
| ER | g-C/m^2^/d |  | Eight Mile Lake | Arctic tundra | 235 | 13.5 | Herb+Shrub | Moist | Growing-season | 1 | Low | 1 | Short | 2.08 | 0.22 | 6 | 2.29 | 0.06 | 6 | Natali etal. (2014) |
| ER | g-C/m^2^/d |  | Eight Mile Lake | Arctic tundra | 235 | 13.5 | Herb+Shrub | Moist | Growing-season | 1 | Low | 2 | Short | 2.23 | 0.22 | 6 | 2.53 | 0.18 | 6 | Natali etal. (2014) |
| ER | g-C/m^2^/d |  | Eight Mile Lake | Arctic tundra | 235 | 13.5 | Herb+Shrub | Moist | Growing-season | 1 | Low | 3 | Short | 1.96 | 0.27 | 6 | 2.33 | 0.14 | 6 | Natali etal. (2014) |
| ER | g-C/m^2^/d |  | Toolik Lake | Arctic tundra | 180 | 10.00 | Shrub | Dry | Year-round | 1–3 |  | 3 | Short | 1.32 | 0.47 | 5 | 2.31 | 0.85 | 5 | Welker etal. (1999) |
| ER | g-C/m^2^/d |  | Toolik Lake | Alpine tundra | 180 | 10.00 | Shrub | Dry | Year-round | 1–3 |  | 3 | Short | 1.59 | 0.44 | 4 | 1.81 | 0.51 | 4 | Welker etal. (1999) |
| ER | g-C/m^2^/d |  | N-Siberia | Arctic tundra | 310 | 11 | Shrub | Dry | Year-round | 3.60 | High | 2 | Short | 0.16 | 0.16 | 9 | 0.70 | 0.44 | 9 | Biasi etal. (2006) |
| ER | g-C/m^2^/d |  | Alexandra Fiord | Arctic tundra | 30 | 8.7 | Shrub | Dry | Year-round | 0.2 | Low | 9 | Long | 5.38 | 0.32 | 3 | 6.40 | 0.30 | 3 | Welker etal. (2004) |
| ER | g-C/m^2^/d |  | Alexandra Fiord | Arctic tundra | 30 | 7.9 | Shrub | Moist | Year-round | 1 | Low | 9 | Long | 3.79 | 0.25 | 4 | 3.99 | 0.32 | 4 | Welker etal. (2004) |
| ER | g-C/m^2^/d |  | Alexandra Fiord | Arctic tundra | 30 | 8.1 | Herb | Wet | Year-round | 0.2 | Low | 9 | Long | 2.78 | 0.25 | 3 | 2.95 | 0.30 | 3 | Welker etal. (2004) |
| ER | g-C/m^2^/d |  | Alexandra Fiord | Arctic tundra | 30 | 8.7 | Shrub | Dry | Growing-season | 1-2 | Low | 9 | Long | 2.62 | 0.90 | 3 | 2.59 | 0.70 | 3 | Oberbauer etal. (2007) |
| ER | g-C/m^2^/d |  | Alexandra Fiord | Arctic tundra | 30 | 8.7 | Shrub | Dry | Growing-season | 1-2 | Low | 10 | Long | 2.40 | 0.93 | 3 | 3.19 | 1.13 | 3 | Oberbauer etal. (2007) |
| ER | g-C/m^2^/d |  | Alexandra Fiord | Arctic tundra | 30 | 7.9 | Shrub | Moist | Growing-season | 1-2 | Low | 9 | Long | 1.41 | 0.79 | 3 | 1.90 | 0.72 | 3 | Oberbauer etal. (2007) |
| ER | g-C/m^2^/d |  | Alexandra Fiord | Arctic tundra | 30 | 7.9 | Shrub | Moist | Growing-season | 1-2 | Low | 10 | Long | 1.88 | 0.92 | 3 | 2.04 | 0.52 | 3 | Oberbauer etal. (2007) |
| ER | g-C/m^2^/d |  | Alexandra Fiord | Arctic tundra | 30 | 8.1 | Herb | Wet | Growing-season | 1-2 | Low | 9 | Long | 1.47 | 1.04 | 3 | 1.54 | 1.15 | 3 | Oberbauer etal. (2007) |
| ER | g-C/m^2^/d |  | Alexandra Fiord | Arctic tundra | 30 | 8.1 | Herb | Wet | Growing-season | 1-2 | Low | 10 | Long | 0.91 | 0.58 | 3 | 1.13 | 0.90 | 3 | Oberbauer etal. (2007) |
| ER | g-C/m^2^/d |  | Barrow | Arctic tundra | 57 | 3.7 | Shrub | Dry | Year-round | 1-2 | Low | 7 | Long | 0.96 | 0.12 | 5 | 2.10 | 0.86 | 5 | Oberbauer etal. (2007) |
| ER | g-C/m^2^/d |  | Barrow | Arctic tundra | 57 | 3.7 | Shrub | Dry | Year-round | 1-2 | Low | 8 | Long | 0.96 | 0.19 | 5 | 1.72 | 0.51 | 5 | Oberbauer etal. (2007) |
| ER | g-C/m^2^/d |  | Barrow | Arctic tundra | 57 | 3.7 | Herb | Wet | Year-round | 1-2 | Low | 6 | Long | 1.34 | 0.21 | 5 | 1.51 | 0.39 | 5 | Oberbauer etal. (2007) |
| ER | g-C/m^2^/d |  | Barrow | Arctic tundra | 57 | 3.7 | Herb | Wet | Year-round | 1-2 | Low | 7 | Long | 1.56 | 0.28 | 5 | 1.61 | 0.44 | 5 | Oberbauer etal. (2007) |
| ER | g-C/m^2^/d |  | Atqasuk | Arctic tundra | 55 | 9 | Shrub | Dry | Year-round | 1-2 | Low | 5 | Long | 0.83 | 0.09 | 5 | 1.15 | 0.14 | 5 | Oberbauer etal. (2007) |
| ER | g-C/m^2^/d |  | Atqasuk | Arctic tundra | 55 | 9 | Shrub | Dry | Year-round | 1-2 | Low | 6 | Long | 0.61 | 0.09 | 5 | 0.86 | 0.16 | 5 | Oberbauer etal. (2007) |
| ER | g-C/m^2^/d |  | Atqasuk | Arctic tundra | 55 | 9 | Herb | Wet | Year-round | 1-2 | Low | 5 | Long | 0.78 | 0.14 | 5 | 0.61 | 0.16 | 5 | Oberbauer etal. (2007) |
| ER | g-C/m^2^/d |  | Atqasuk | Arctic tundra | 55 | 9 | Herb | Wet | Year-round | 1-2 | Low | 6 | Long | 1.00 | 0.14 | 5 | 0.89 | 0.16 | 5 | Oberbauer etal. (2007) |
| ER | g-C/m^2^/d |  | Toolik Lake | Arctic tundra | 180 | 10.00 | Shrub | Dry | Year-round | 1-2 | Low | 3 | Short | 1.87 | 0.35 | 3 | 2.71 | 0.95 | 3 | Oberbauer etal. (2007) |
| ER | g-C/m^2^/d |  | Toolik Lake | Arctic tundra | 180 | 10.00 | Shrub | Dry | Year-round | 1-2 | Low | 4 | Long | 1.58 | 1.20 | 3 | 2.43 | 0.81 | 3 | Oberbauer etal. (2007) |
| ER | g-C/m^2^/d |  | Toolik Lake | Arctic tundra | 180 | 10.00 | Herb | Moist | Year-round | 1-2 | Low | 3 | Short | 2.22 | 1.31 | 3 | 2.03 | 0.83 | 3 | Oberbauer etal. (2007) |
| ER | g-C/m^2^/d |  | Toolik Lake | Arctic tundra | 180 | 10.00 | Herb | Moist | Year-round | 1-2 | Low | 4 | Long | 3.04 | 0.68 | 3 | 2.77 | 0.65 | 3 | Oberbauer etal. (2007) |
| ER | g-C/m^2^/d |  | Seida | Arctic tundra | 450 | 12.5 | Moss | Wet | Growing-season | 0.95 | Low | 2 | Short | 1.68 | 0.51 | 5 | 2.82 | 0.34 | 5 | Voigt etal. (2017) |
| ER | g-C/m^2^/d |  | Seida | Arctic tundra | 450 | 13.4 | Shrub | Wet | Growing-season | 0.95 | Low | 2 | Short | 5.11 | 1.19 | 5 | 6.33 | 2.56 | 5 | Voigt etal. (2017) |
| ER | g-C/m^2^/d |  | Seida | Arctic tundra | 450 | 12.5 | Shrub | Dry | Growing-season | 0.95 | Low | 2 | Short | 7.40 | 2.05 | 5 | 10.60 | 1.70 | 5 | Voigt etal. (2017) |
| ER | g-C/m^2^/d |  | Seida | Arctic tundra | 450 | 12.5 | Moss | Wet | Growing-season | 0.95 | Low | 3 | Short | 1.77 | 0.44 | 5 | 2.43 | 0.44 | 5 | Voigt etal. (2017) |
| ER | g-C/m^2^/d |  | Seida | Arctic tundra | 450 | 13.4 | Shrub | Wet | Growing-season | 0.95 | Low | 3 | Short | 5.64 | 1.61 | 6 | 6.75 | 2.20 | 5 | Voigt etal. (2017) |
| ER | g-C/m^2^/d |  | Seida | Arctic tundra | 450 | 12.5 | Shrub | Dry | Growing-season | 0.95 | Low | 3 | Short | 5.38 | 1.91 | 7 | 6.43 | 0.88 | 5 | Voigt etal. (2017) |
| ER | g-C/m^2^/d |  | Toolik Lake | Arctic tundra | 180 | 10.00 | Moss | Wet | Growing-season | 2-5 | High | 11 | Long | 3.32 | 1.43 | 5 | 2.65 | 1.64 | 5 | Leffler etal. (2016) |
| ER | g-C/m^2^/d |  | Eight Mile Lake | Arctic tundra | 235 | 13.5 | Herb+Shrub | Moist | Growing-season | 2.20 | High | 1 | Short | 1.31 | 0.28 | 5 | 1.55 | 0.33 | 5 | Natali etal. (2011) |
| ER | g-C/m^2^/d |  | Toolik Lake | Arctic tundra | 180 | 10.00 | Herb | Wet | Growing-season |  |  | 14 | Long | 3.50 | 1.01 | 5 | 3.69 | 0.15 | 5 | Boelman etal. (2003) |
| ER | g-C/m^2^/d |  | Abisko | Subarctic heath | 120.00 | 8.90 | Shrub | Wet | Growing-season | 3.9 | High |  |  | 2.23 | 0.74 | 4 | 2.97 | 0.85 | 4 | Christensen etal.(1997) |
| ER | g-C/m^2^/d |  | Abisko | Subarctic heath | 120.00 | 12.70 | Shrub | Dry | Growing-season | 2.40 | High | 10 | Long | 1.94 | 0.46 | 6 | 2.22 | 0.53 | 6 | Illeris etal.(2004) |
| ER | g-C/m^2^/d |  | Abisko | Subarctic heath | 120.00 | 11.90 | Shrub | Wet | Growing-season | 1 | Low | 16 | Long | 2.35 | 0.21 | 6 | 3.45 | 0.63 | 6 | Pedersen etal.(2017) |
| ER | g-C/m^2^/d |  | Fenghuoshan | Alpine swamp meadow | 216 | 1.42 | Herb | Wet | Year-round | 6.2 | High | 4 | Long | 8.46 | 1.24 | 3 | 11.07 | 1.46 | 3 | Chen etal.(2017) |
| ER | g-C/m^2^/d |  | Gangcha | Alpine swamp meadow | 315.00 | 11 | Herb | Wet | Year-round | >1.6 | High | 2 | Short | 6.88 | 4.12 | 16 | 7.65 | 2.87 | 16 | Li etal.(2017) |
| ER | g-C/m^2^/d |  | Gangcha | Alpine swamp meadow | 315.00 | 11 | Herb | Wet | Year-round | >1.6 | High | 3 | Short | 5.98 | 2.65 | 16 | 6.25 | 2.71 | 16 | Li etal.(2017) |
| ER | g-C/m^2^/d |  | Gangcha | Alpine swamp meadow | 315.00 | 11 | Herb | Wet | Year-round | >1.6 | High | 4 | Long | 6.80 | 2.77 | 16 | 7.04 | 2.52 | 16 | Li etal.(2017) |
| ER | g-C/m^2^/d |  | Nagqu | Alpine meadow | 370 | 8.5 | Herb | Moist | Year-round | 2.20 | High | 1 | Short | 3.48 | 0.56 | 2 | 3.25 | 1.49 | 2 | Zhu etal. (2017) |
| ER | g-C/m^2^/d |  | Nagqu | Alpine meadow | 370 | 8.5 | Herb | Moist | Year-round | 2.20 | High | 2 | Short | 1.99 | 0.10 | 2 | 2.69 | 0.10 | 2 | Zhu etal. (2017) |
| ER | g-C/m^2^/d |  | Damxung | Alpine meadow | 405 | 10.7 | Herb | Moist | Year-round | 1.6 | Low | 3 | Short | 3.63 | 2.90 | 5 | 2.85 | 2.90 | 5 | Zong etal. (2018) |
| ER | g-C/m^2^/d |  | Damxung | Alpine meadow | 405 | 10.7 | Herb | Moist | Year-round | 1.6 | Low | 4 | Long | 2.14 | 0.51 | 5 | 2.26 | 0.19 | 5 | Zong etal. (2018) |
| ER | g-C/m^2^/d |  | Damxung | Alpine meadow | 405 | 10.7 | Herb | Moist | Year-round | 1.6 | Low | 5 | Long | 4.56 | 4.57 | 5 | 3.12 | 2.62 | 5 | Zong etal. (2018) |
| ER | g-C/m^2^/d |  | Fenghuoshan | Alpine meadow | 216 | 2.5 | Herb | Moist | Year-round | 1.25 | Low | 1 | Short | 1.63 | 0.83 | 3 | 2.18 | 0.47 | 3 | Bai etal. (2011) |
| ER | g-C/m^2^/d |  | Fenghuoshan | Alpine meadow | 216 | 2.5 | Herb | Moist | Year-round | 3.68 | High | 1 | Short | 1.63 | 0.83 | 3 | 4.35 | 1.91 | 3 | Bai etal. (2011) |
| ER | g-C/m^2^/d |  | Abisko | Sub-arctic peatland | 120.00 | 11.30 | Moss | Dry | Growing-season | 0.2-0.9 | Low | 4 | Long | 4.27 | 1.19 | 5 | 7.15 | 1.91 | 5 | Dorrepaal etal.(2009) |
| ER | g-C/m^2^/d |  | Abisko | Sub-arctic peatland | 120.00 | 11.30 | Moss | Dry | Growing-season | 0.2-0.9 | Low | 6 | Long | 12.80 | 5.25 | 5 | 17.60 | 2.62 | 5 | Dorrepaal etal.(2009) |
| ER | g-C/m^2^/d |  | Abisko | Sub-arctic peatland | 120.00 | 11.30 | Moss | Dry | Growing-season | 0.2-0.9 | Low | 7 | Long | 8.00 | 2.15 | 5 | 13.23 | 2.39 | 5 | Dorrepaal etal.(2009) |
| ER | g-C/m^2^/d |  | Abisko | Sub-arctic peatland | 120.00 | 11.30 | Moss | Dry | Growing-season | 0.2-0.9 | Low | 8 | Long | 8.00 | 2.15 | 5 | 15.04 | 3.58 | 5 | Dorrepaal etal.(2009) |
| ER | g-C/m^2^/d |  | Haibei | Alpine meadow | 370 | 8.5 | Herb | Moist | Year-round | >1 |  | 2 | Short | 6.56 | 0.70 | 6 | 7.13 | 0.56 | 6 | Chen etal.(2016) |
| ER | g-C/m^2^/d |  | Haibei | Alpine meadow | 370 | 8.5 | Herb | Moist | Year-round | >1 |  | 3 | Short | 8.24 | 0.57 | 6 | 9.61 | 0.70 | 6 | Chen etal.(2016) |
| ER | g-C/m^2^/d |  | Haibei | Alpine meadow | 370 | 8.5 | Herb | Moist | Year-round | >1 |  | 4 | Long | 7.44 | 0.43 | 6 | 7.68 | 0.70 | 6 | Chen etal.(2016) |
| ER | g-C/m^2^/d |  | Damxung | Alpine meadow | 405 | 10.7 | Herb | Moist | Year-round | 1-1.4 | Low | 3 | Short | 3.13 | 0.82 | 7 | 1.58 | 0.36 | 7 | Fu etal.(2013) |
| ER | g-C/m^2^/d |  | Damxung | Alpine meadow | 405 | 10.7 | Herb | Moist | Year-round | 1-1.4 | Low | 4 | Long | 4.05 | 0.98 | 7 | 2.97 | 0.70 | 7 | Fu etal.(2013) |
| ER | g-C/m^2^/d |  | Damxung | Alpine meadow | 405 | 10.7 | Herb | Moist | Year-round | 1-1.4 | Low | 5 | Long | 3.03 | 1.33 | 7 | 2.43 | 1.18 | 7 | Fu etal.(2013) |
| ER | g-C/m^2^/d |  | Damxung | Alpine meadow | 405 | 10.7 | Herb | Moist | Year-round | 1-1.4 | Low | 3 | Short | 3.17 | 1.06 | 7 | 2.41 | 1.24 | 7 | Fu etal.(2013) |
| ER | g-C/m^2^/d |  | Damxung | Alpine meadow | 405 | 10.7 | Herb | Moist | Year-round | 1-1.4 | Low | 4 | Long | 3.76 | 1.12 | 7 | 3.65 | 1.47 | 7 | Fu etal.(2013) |
| ER | g-C/m^2^/d |  | Damxung | Alpine meadow | 405 | 10.7 | Herb | Moist | Year-round | 1-1.4 | Low | 5 | Long | 3.84 | 1.29 | 7 | 3.63 | 1.49 | 7 | Fu etal.(2013) |
| ER | g-C/m^2^/d |  | Damxung | Alpine meadow | 405 | 10.7 | Herb | Moist | Year-round | 1-1.4 | Low | 3 | Short | 3.78 | 0.92 | 7 | 4.09 | 1.40 | 7 | Fu etal.(2013) |
| ER | g-C/m^2^/d |  | Damxung | Alpine meadow | 405 | 10.7 | Herb | Moist | Year-round | 1-1.4 | Low | 4 | Long | 4.56 | 1.24 | 7 | 5.49 | 1.36 | 7 | Fu etal.(2013) |
| ER | g-C/m^2^/d |  | Damxung | Alpine meadow | 405 | 10.7 | Herb | Moist | Year-round | 1-1.4 | Low | 5 | Long | 4.39 | 1.54 | 7 | 5.49 | 1.63 | 7 | Fu etal.(2013) |
| ER | g-C/m^2^/d |  | Beiluhe | Alpine meadow | 276.00 | 6 | Herb | Moist | Year-round | 2.30 | High | 1 | Short | 3.23 | 1.22 | 5 | 4.38 | 2.39 | 5 | Peng et al. (2016) |

1. The data collected for meta-analysis were all from warming experiments conducted in alpine, subarctic, and arctic biomes of the northern hemisphere. As we focused on air warming effects, only warming experiments used methods of infrared radiators (IR), open-top chambers (OTCs), snow fences, and greenhouse were included. Thus, we did not included soil warming experiments using soil heating cables into analysis as Wang *et al*., 2019 (See Wang *et al*., 2019, Effects of climate warming on carbon fluxes in grasslands - a global meta-analysis. Global Change Biology).
2. Note that to test for differences in responses of functional groups, vegetation types were classified to moss, herb, herb and shrub, and shrub.
3. Soil moisture status was characterized to dry, moist, and wet. For sites where soil moisture status was not specified (e.g. Fu *et al*, 2012, 2013, 2015, 2018, and 2019), we characterized it based on soil texture, soil moisture content, and water holding capacity. Soil moisture status was identified according to the ratio of soil moisture content to water holding capacity (<60%: dry, 60—100%: moist, >100%, wet).
4. As reports on non-growing season warming effects for alpine and arctic ecosystems were rather limited (e.g. Fu *et al*., 2019), we took only growing-season and year-round warming into consideration.
5. Air temperature increases varied from 0.2 to 6.2 ℃, with 2℃ chosen as the subdividing point (low warming: ≤2℃, high warming: > 2℃). For some studies that did not report warming effects on air temperature (e.g. Li *et al.,* 20017), we characterzied warming levels according to soil temperature increase. If soil temperature increase exceeded 1.6 ℃, the treatment was considered as high-level warming.
6. The warming time ranged from 1 to 21 growing seasons, and was grouped into short- and long-term warming subdivided by 3 growing seasons.
7. Admittedly, warming approaches could affect warming effects (Wang *et al*., 2019, see above). Unlike IR, passive warming, such as OTC, has actually no capability to set a fixed temperature varying from few to several degrees along the day, and increases temperature only during daytime. However, a recent study has confirmed that warming methods have no differences in their effects on alpine AGB, BGB, SOC, MBC, and TN (Chen *et al*., 2020, Effects of warming on carbon and nitrogen cycling in alpine grassland ecosystems on the Tibetan Plateau: A meta-analysis. Geodera, 370, 114363). Thus, in the current study we did not analyze their effects.
8. GEP—gross ecosystem productivity, ER—ecosystem respiration, NEE—net ecosystem C exchange, AGB—aboveground biomass, BGB—belowground biomass, SOC—soil organic C, and MBC—microbial biomass.

**References**

Alatalo, J.M., Jägerbrand, A.K., Juhanson, J., Michelsen, A. and Ľuptáčik, P., 2017. Impacts of twenty years of experimental warming on soil carbon, nitrogen, moisture and soil mites across alpine/subarctic tundra communities. Scientific Reports, 7: 44489.

Bai, W., Wang, G. and Liu, G., 2011. Effects of elevated temperature on CO_2_ flux during growth season in an alpine meadow ecosystem of Qinghai-Tibet Plateau. Chinese Journal of Ecology, 30(06): 1045-1051.

Biasi, C., Meyer, H., Rusalimova, O., Hämmerle, R., Kaiser, C., Baranyi, C., Daims, H., Lashchinsky, N., Barsukov, P. and Richter, A., 2008. Initial effects of experimental warming on carbon exchange rates, plant growth and microbial dynamics of a lichen-rich dwarf shrub tundra in Siberia. Plant and Soil, 307(1-2): 191-205.

Boelman, N.T., Stieglitz, M., Rueth, H.M., Sommerkorn, M., Griffin, K.L., Shaver, G.R. and Gamon, J.A., 2003. Response of NDVI, biomass, and ecosystem gas exchange to long-term warming and fertilization in wet sedge tundra. Oecologia, 135(3): 414-421.

Chang, R., Wang, G., Yang, Y. and Chen, X., 2017. Experimental warming increased soil nitrogen sink in the Tibetan permafrost. Journal of Geophysical Research Biogeosciences, 122(7): 1870-1879.

Chapin III, F.S., Shaver, G.R., Giblin, A.E., Nadelhoffer, K.J. and Laundre, J.A., 1995. Responses of arctic tundra to experimental and observed changes in climate. Ecology, 76(3): 694-711.

Chen, X., Wang, G., Zhang, T., Mao, T., Wei, D., Song, C., Hu, Z. and Huang, K., 2017. Effects of warming and nitrogen fertilization on GHG flux in an alpine swamp meadow of a permafrost region. Science of the Total Environment, 601-602: 1389-1399.

Christensen, T.R., Michelsen, A., Jonasson, S. and Schmidt, I.K., 1997. Carbon dioxide and methane exchange of a subarctic heath in response to climate change related environmental manipulations. Oikos: 34-44.

Deane-Coe, K.K., Mauritz, M., Celis, G., Salmon, V., Crummer, K.G., Natali, S.M. and Schuur, E.A., 2015. Experimental warming alters productivity and isotopic signatures of tundra mosses. Ecosystems, 18(6): 1070-1082.

DeMarco, J., Mack, M.C., Bret-Harte, M.S., Burton, M. and Shaver, G.R., 2014. Long‐term experimental warming and nutrient additions increase productivity in tall deciduous shrub tundra. Ecosphere, 5(6): 1-22.

Dorji, T., Totland, Ø., Moe, S.R., Hopping, K.A., Pan, J. and Klein, J.A., 2013. Plant functional traits mediate reproductive phenology and success in response to experimental warming and snow addition in Tibet. Global Change Biology, 19(2): 459-472.

Dorrepaal, E., Toet, S., Logtestijn, R.S.P.V., Swart, E., Weg, M.J.V.D., Callaghan, T.V. and Aerts, R., 2009. Carbon respiration from subsurface peat accelerated by climate warming in the subarctic. Nature, 460(7255): 616-619.

Fu, G., Shen, Z.-X. and Zhang, X.-Z., 2018. Increased precipitation has stronger effects on plant production of an alpine meadow than experimental warming in the Northern Tibetan Plateau. Agricultural and Forest Meteorology, 249: 11-21.

Fu, G., Shen, Z., Zhang, X. and Zhou, Y., 2012. Response of soil microbial biomass to short-term experimental warming in alpine meadow on the Tibetan Plateau. Applied Soil Ecology, 61(61): 158-160.

Fu, G., Wei, S., Cheng-Qun, Y., Xian-Zhou, Z., Zhen-Xi, S., Yun-Long, L., Peng-Wan, Y. and Nan, Z., 2015. Clipping Alters the Response of Biomass Production to Experimental Warming: A Case Study in an Alpine Meadow on the Tibetan Plateau, China. Journal of Mountain Science, 12(4): 935-942.

Fu, G., Zhang, H.R. and Sun, W., 2019. Response of plant production to growing/non-growing season asymmetric warming in an alpine meadow of the Northern Tibetan Plateau. Science of the Total Environment, 650: 2666-2673.

Fu, G., Zhang, Y.J., Zhang, X.Z., Shi, P.L., Zhou, Y.T., Li, Y.L. and Shen, Z.X., 2013. Response of ecosystem respiration to experimental warming and clipping in Tibetan alpine meadow at three elevations. Biogeosciences Discussions, 10(8): 13015-13047.

Ganjurjav, H., Gao, Q., Gornish, E.S., Schwartz, M.W., Liang, Y., Cao, X., Zhang, W., Zhang, Y., Li, W. and Wan, Y., 2016. Differential response of alpine steppe and alpine meadow to climate warming in the central Qinghai–Tibetan Plateau. Agricultural & Forest Meteorology, 223: 233-240.

Geng, X., Xu, r. and Wei, D., 2017. Response of greenhouse gases fluxto multi-level warming in an alpine meadow of TibetanPlateau. Ecology and Environmental Sciences, 26(003): 445-452.

Gill, R.A., 2014. The influence of 3-years of warming and N-deposition on ecosystem dynamics is small compared to past land use in subalpine meadows. Plant & Soil, 374(1-2): 197-210.

Guan, S., An, N., Zong, N., He, Y., Shi, P., Zhang, J. and He, N., 2018. Climate warming impacts on soil organic carbon fractions and aggregate stability in a Tibetan alpine meadow. Soil Biology and Biochemistry, 116: 224-236.

Guo, H., De, K., Lu, G. and Wang, W., 2015. The Impacts of Simulative Warming and Adding Nitrogen on the Grassland Productivity of Alpine Meadow. Acta Agrestia Sinica, 23(2): 322-327.

Heng, T., Wu, J., Xie, S. and Wu, M., 2011. The Responses of Soil C and N,Microbial Biomass C or N under Alpine Meadow of Qinghai-Tibet Plateau to the Change of Temperature and Precipitation. Chinese Agricultural Science Bulletin.

Hobbie, S.E. and Chapin III, F.S., 1998. The response of tundra plant biomass, aboveground production, nitrogen, and CO_2_ flux to experimental warming. Ecology, 79(5): 1526-1544.

Hollister, R.D. and Flaherty, K.J., 2010. Above‐and below‐ground plant biomass response to experimental warming in northern Alaska. Applied Vegetation Science, 13(3): 378-387.

Hu, Y., Chang, X., Lin, X., Wang, Y., Wang, S., Duan, J., Zhang, Z., Yang, X., Luo, C. and Xu, G., 2010. Effects of warming and grazing on N_2_O fluxes in an alpine meadow ecosystem on the Tibetan plateau. Soil Biology & Biochemistry, 42(6): 944-952.

Hudson, J.M. and Henry, G.H., 2010. High Arctic plant community resists 15 years of experimental warming. Journal of Ecology, 98(5): 1035-1041.

Illeris, L., König, S.M., Grogan, P., Jonasson, S., Michelsen, A. and Ro-Poulsen, H., 2004. Growing-season carbon dioxide flux in a dry subarctic heath: responses to long-term manipulations. Arctic, Antarctic, and Alpine Research, 36(4): 456-463.

Ji, C., Luo, Y., Xia, J., Zheng, S., Jiang, L., Niu, S., Zhou, X. and Cao, J., 2016. Differential responses of ecosystem respiration components to experimental warming in a meadow grassland on the Tibetan Plateau. Agricultural & Forest Meteorology, 220(220): 21-29.

Jiang, Y.-B., Fan, M. and Zhang, Y.-J., 2017. Effect of short-term warming on plant community features of alpine meadow in Northern Tibet. Chinese Journal of Ecology, 36(3): 616-622.

Jing, X., Wang, Y., Chung, H., Mi, Z., Wang, S., Zeng, H. and He, J.-S., 2014. No temperature acclimation of soil extracellular enzymes to experimental warming in an alpine grassland ecosystem on the Tibetan Plateau. Biogeochemistry, 117(1): 39-54.

Lamb, E.G., Han, S., Lanoil, B.D., Henry, G.H.R., Brummell, M.E., Banerjee, S. and Siciliano, S.D., 2011. A High Arctic soil ecosystem resists long-term environmental manipulations. Global Change Biology, 17(10): 3187-3194.

Leffler, A.J., Klein, E.S., Oberbauer, S.F. and Welker, J.M., 2016. Coupled long-term summer warming and deeper snow alters species composition and stimulates gross primary productivity in tussock tundra. Oecologia, 181(1): 287-297.

Li, F., Peng, Y., Natali, S.M., Chen, K., Han, T., Yang, G., Ding, J., Zhang, D., Wang, G., Wang, J., Yu, J., Liu, F. and Yang, Y., 2017. Warming effects on permafrost ecosystem carbon fluxes associated with plant nutrients. Ecology, 98(11): 2851-2859.

Li, F., Peng, Y., Zhang, D., Yang, G., Fang, K., Wang, G., Wang, J., Yu, J., Zhou, G. and Yang, Y., 2019a. Leaf area rather than photosynthetic rate determines the response of ecosystem productivity to experimental warming in an alpine steppe. Journal of Geophysical Research: Biogeosciences.

Li, N., Wang, G., Yan, Y., Gao, Y., Liu, L. and Liu, G., 2011. Short-term effects of temperature enhancement on community structure and biomass of alpine meadow in the Qinghai-Tibet Plateau. Acta Ecologica Sinica, 31(4): 895-905.

Li, N., Wang, G.X., Gao, Y.H., Wang, J.F. and Liu, L.A., 2010. Effects of simulated warming on soil nutrients and biological characteristics of alpine meadow soil in the headwaters region of the yangtze river. Acta Pedologica Sinica.

Li, Y., Hasbagan, G., Hu, G., Wan, Y. and Gao, Q., 2019b. Effects of warming on carbon exchange in an alpine steppe in the Tibetan Plateau. Acta Ecologica Sinica, 39(6): 2004-2012.

Lin, X., Zhang, Z., Wang, S., Hu, Y., Xu, G., Luo, C., Chang, X., Duan, J., Lin, Q. and Xu, B., 2011. Response of ecosystem respiration to warming and grazing during the growing seasons in the alpine meadow on the Tibetan plateau. Agricultural & Forest Meteorology, 151(7): 792-802.

Liu, H., Mi, Z., Lin, L., Wang, Y., Zhang, Z., Zhang, F., Wang, H., Liu, L., Zhu, B. and Cao, G., 2018. Shifting plant species composition in response to climate change stabilizes grassland primary production. Proc Natl Acad Sci U S A, 115(16): 4051-4056.

Liu, W., Wang, C.T., Zhao, J.Z., Qing-Min, X.U. and Zhou, L., 2010. Responses of Quantity Characteristics of Plant Community to Simulating Warming in Alpine Kobresia humilis Meadow Ecosystem. Acta Botanica Boreali-Occidentalia Sinica, 30(5): 995-1003.

Luo, C.Y., Xu, G.P., Chao, Z.G., Wang, S.P., Lin, X.W., Hu, Y.G., Zhang, Z.H., Duan, J.C., Chang, X.F. and Su, A.L., 2010. Effect of warming and grazing on litter mass loss and temperature sensitivity of litter and dung mass loss on the Tibetan plateau. Global Change Biology, 16(5): 1606-1617.

Ma, S., Zhu, X., Zhang, J., Zhang, L., Che, R., Wang, F., Liu, H., Niu, H., Wang, S. and Cui, X., 2015. Warming decreased and grazing increased plant uptake of amino acids in an alpine meadow. Ecology & Evolution, 5(18): 3995.

Michelsen, A., Jonasson, S., Sleep, D., Havström, M. and Callaghan, T.V., 1996. Shoot biomass, δ^13^C, nitrogen and chlorophyll responses of two arctic dwarf shrubs to in situ shading, nutrient application and warming simulating climatic change. Oecologia, 105(1): 1-12.

Natali, S.M., Schuur, E.A., Trucco, C., Hicks Pries, C.E., Crummer, K.G. and Baron Lopez, A.F., 2011. Effects of experimental warming of air, soil and permafrost on carbon balance in Alaskan tundra. Global Change Biology, 17(3): 1394-1407.

Natali, S.M., Schuur, E.A., Webb, E.E., Pries, C.E.H. and Crummer, K.G., 2014. Permafrost degradation stimulates carbon loss from experimentally warmed tundra. Ecology, 95(3): 602-608.

Oberbauer, S.F., Tweedie, C.E., Welker, J.M., Fahnestock, J.T., Henry, G.H., Webber, P.J., Hollister, R.D., Walker, M.D., Kuchy, A. and Elmore, E., 2007. Tundra CO_2_ fluxes in response to experimental warming across latitudinal and moisture gradients. Ecological Monographs, 77(2): 221-238.

Pedersen, E.P., Elberling, B. and Michelsen, A., 2017. Seasonal variations in methane fluxes in response to summer warming and leaf litter addition in a subarctic heath ecosystem. Journal of Geophysical Research: Biogeosciences, 122(8): 2137-2153.

Peng, F., Xue, X., You, Q., Xu, M., Chen, X., Guo, J. and Wang, T., 2016. Intensified plant N and C pool with more available nitrogen under experimental warming in an alpine meadow ecosystem. Ecology & Evolution, 6(23): 8546-8555.

Peng, F., Xue, X., You, Q., Zhou, X. and Wang, T., 2015. Warming effects on carbon release in a permafrost area of Qinghai-Tibet Plateau. Environmental Earth Sciences, 73(1): 57-66.

Peng, F., You, Q., Xu, M., Guo, J., Wang, T. and Xue, X., 2014. Effects of Warming and Clipping on Ecosystem Carbon Fluxes across Two Hydrologically Contrasting Years in an Alpine Meadow of the Qinghai-Tibet Plateau. Plos One, 9(10): e109319.

Qin, Y., Yi, S., Chen, J., Ren, S. and Wang, X., 2015. Responses of ecosystem respiration to short-term experimental warming in the alpine meadow ecosystem of a permafrost site on the Qinghai–Tibetan Plateau. Cold Regions Ence & Technology, 115(jul.): 77-84.

Richardson, S.J., Press, M.C., Parsons, A.N. and Hartley, S.E., 2002. How do nutrients and warming impact on plant communities and their insect herbivores? A 9‐year study from a sub‐Arctic heath. Journal of Ecology, 90(3): 544-556.

Rinnan, R., Michelsen, A. and Jonasson, S., 2008. Effects of litter addition and warming on soil carbon, nutrient pools and microbial communities in a subarctic heath ecosystem. Applied Soil Ecology, 39(3): 271-281.

Rinnan, R., Stark, S. and Tolvanen, A., 2009. Responses of vegetation and soil microbial communities to warming and simulated herbivory in a subarctic heath. Journal of Ecology, 97(4): 788-800.

Rui, Y., Wang, S., Xu, Z., Wang, Y., Chen, C., Zhou, X., Kang, X., Lu, S., Hu, Y. and Lin, Q., 2011. Warming and grazing affect soil labile carbon and nitrogen pools differently in an alpine meadow of the Qinghai–Tibet Plateau in China. Journal of Soils & Sediments, 11(6): 903-914.

Schmidt, I.K., Jonasson, S., Shaver, G.R., Michelsen, A. and Nordin, A., 2002. Mineralization and distribution of nutrients in plants and microbes in four arctic ecosystems: responses to warming. Plant & Soil, 242(1): 93-106.

Shen, Z.X., Li, Y.L. and Fu, G., 2015. Response of soil respiration to short-term experimental warming and precipitation pulses over the growing season in an alpine meadow on the Northern Tibet. Applied Soil Ecology, 90(90): 35-40.

Shi, F., Chen, H., Wu, Y. and Wu, N., 2012. The combined effects of warming and drying suppress CO_2_ and N_2_O emission rates in an alpine meadow of the eastern Tibetan Plateau. Ecological Research, 27(4): 725-733.

Shi, F.S., 2008. Effect of Experimental Warming on Carbon and Nitrogen Content of Sub-alpine Meadow in Northwestern Sichuan. Bulletin of Botanical Research, 28(6): 730-736.

Sistla, S.A., Moore, J.C., Simpson, R.T., Gough, L., Shaver, G.R. and Schimel, J.P., 2013. Long-term warming restructures Arctic tundra without changing net soil carbon storage. Nature, 497(7451): 615.

Sorensen, P.L. and Michelsen, A., 2011. Long-term warming and litter addition affects nitrogen fixation in a subarctic heath. Global Change Biology, 17(1): 528-537.

Voigt, C., Lamprecht, R.E., Marushchak, M.E., Lind, S.E., Novakovskiy, A., Aurela, M., Martikainen, P.J. and Biasi, C., 2017. Warming of subarctic tundra increases emissions of all three important greenhouse gases–carbon dioxide, methane, and nitrous oxide. Global Change Biology, 23(8): 3121-3138.

Wang, B., Sun, G., Luo, P., Wang, M. and Wu, N., 2011. Labile and recalcitrant carbon and nitrogen pools of an alpine meadow soil from the eastern Qinghai-Tibetan Plateau subjected to experimental warming and grazing. Acta Ecologica Sinica, 10(10): 1109-1122.

Wang, J., Wang, G.Q., Fei, L.I., Peng, Y.F., Yang, G.B., Jian-Chun, Y.U., Zhou, G.Y. and Yang, Y.H., 2018a. Effects of short-term experimental warming on soil microbes in a typical alpine steppe. Chinese Journal of Plant Ecology.

Wang, X., Dong, S., Gao, Q., Zhou, H., Liu, S., Su, X. and Li, Y., 2014a. Effects of short-term and long-term warming on soil nutrients, microbial biomass and enzyme activities in an alpine meadow on the Qinghai-Tibet Plateau of China. Soil Biology & Biochemistry, 76: 140-142.

Wang, X., Yumei, Z., Xiaojie, J. and Shijie, H., 2014b. Effects of warming on soil microbial community structure in Changbai Mountain Tundra. Acta Ecologica Sinica, 34(20).

Wang, X., Zhou, Y., Jiang, X. and Han, S., 2014c. Effects of warming on soil microbial community structure in Changbai Mountain Tundra. Acta Ecologica Sinica, 34(20).

Wang, X.X., Gao, Q.Z., Ganjurjav, H., Guo-Zheng, H.U., Wen-Han, L.I. and Luo, W.R., 2018b. Response of Greenhouse Gases Emission Fluxes to Long-term Warming in Alpine Meadow of Northern Tibet. Chinese Journal of Agrometeorology.

Welker, J., Brown, K. and Fahnestock, J., 1999. CO_2_ flux in arctic and alpine dry tundra: comparative field responses under ambient and experimentally warmed conditions. Arctic, Antarctic, and Alpine Research, 31(3): 272-277.

Welker, J.M., Fahnestock, J.T., Henry, G.H.R., O'Dea, K.W. and Chimner, R.A., 2004. CO2 exchange in three Canadian High Arctic ecosystems: response to long-term experimental warming. Global Change Biology, 10(12): 1981-1995.

Wu, D., Jing, X., Lin, L., Yang, X. and He, J., 2016. Responses of Soil Inorganic Nitrogen to Warming and Altered Precipitation in an Alpine Meadow on the Qinghai-Tibetan Plateau. Acta Scientiarum Naturalium Universitatis Pekinensis.

Xi, J., Bai, W., Pengsong, Y. and Yongwan, L., 2019. Effects of simulated warming on soil organic carbon composition and biomass in alpine swamp meadow in the headwaters region of the Yangtze River. Ecological Science, 38(1): 92-101.

Xiong, Q., Pan, K., Zhang, L., Wang, Y., Li, W., He, X. and Luo, H., 2016. Warming and nitrogen deposition are interactive in shaping surface soil microbial communities near the alpine timberline zone on the eastern Qinghai–Tibet Plateau, southwestern China. Applied Soil Ecology, 101: 72-83.

Xu, M.-h., Liu, M., Xian, X., Da-tong, Z., Fei, P. and Quan-gang, Y., 2016. Dynamic changes in biomass and its relationship with environmental factors in an alpine meadow on the Qinghai-Tibetan Plateau,based on simulated warming experiments. Acta Ecologica Sinica, 36(18).

Xu, M.-H., Peng, F., You, Q.-G., Guo, J., Tian, X.-F., Liu, M. and Xue, X., 2014. Initial Effects of Experimental Warming on Temperature, Moisture, and Vegetation Characteristics in an Alpine Meadow on the Qinghai-Tibetan Plateau. Polish Journal of Ecology, 62(3): 491-507, 17.

Xu, M.H., Liu, M., Xue, X., Zhai, D.T. and Liu, Z.Q., 2015. Effects of warming and clipping on vegetation species diversity and belowground biomass in an alpine meadow. Chinese Journal of Ecology, 34(9): 2432-2439.

Xue, N., Fei, P., Quangang, Y., Manhou, X. and Siyang, D., 2015. Belowground carbon responses to experimental warming regulated by soil moisture change in an alpine ecosystem of the Qinghai-Tibet Plateau. Ecology & Evolution, 5(18): 4063.

Yang, X., Lin, L., Li, Y. and He, J., 2017. Effects of Warming and Altered Precipitation on Soil Physical Properties and Carbon Pools in a Tibetan Alpine Grassland. Beijing Daxue Xuebao (Ziran Kexue Ban)/Acta Scientiarum Naturalium Universitatis Pekinensis, 53: 765-774.

Ylänne, H., Stark, S. and Tolvanen, A., 2015. Vegetation shift from deciduous to evergreen dwarf shrubs in response to selective herbivory offsets carbon losses: evidence from 19 years of warming and simulated herbivory in the subarctic tundra. Global Change Biology, 21(10): 3696-3711.

Yu, C.-Q., Shen, Z.-X., Zhang, X.-Z., Sun, W. and Fu, G., 2014. Response of Soil C and N, Dissolved Organic C and N, and Inorganic N to Short-Term Experimental Warming in an Alpine Meadow on the Tibetan Plateau. Scientific World Journal.

Yu, C.-Q., Wang, J.-W., Shen, Z.-X. and Fu, G., 2019. Effects of experimental warming and increased precipitation on soil respiration in an alpine meadow in the Northern Tibetan Plateau. Science of the Total Environment, 647: 1490-1497.

Zamin, T.J., Bret‐Harte, M.S. and Grogan, P., 2014. Evergreen shrubs dominate responses to experimental summer warming and fertilization in Canadian mesic low arctic tundra. Journal of Ecology, 102(3): 749-766.

Zhang, B., Chen, S., He, X., Liu, W., Zhao, Q., Zhao, L. and Tian, C., 2014. Responses of Soil Microbial Communities to Experimental Warming in Alpine Grasslands on the Qinghai-Tibet Plateau. Plos One, 9(8): e103859.

Zhang, B., Chen, S., Zhang, J., He, X. and Tian, C., 2015a. Depth-related respopnse of soil microbial communities to experimental warming in an alpine meadow on the Qinghai-Tibet Plateau. European Journal of Soil Science, 66(3): 496-504.

Zhang, Y., Gao, Q., Dong, S., Liu, S., Wang, X., Su, X., Li, Y., Tang, L., Wu, X. and Zhao, H., 2015b. Effects of grazing and climate warming on plant diversity, productivity and living state in the alpine rangelands and cultivated grasslands of the Qinghai-Tibetan Plateau. The Rangeland Journal, 37(1): 57-65.

Zhang, Y. and Welker, J.M., 1996. Tibetan alpine tundra responses to simulated changes in climate: aboveground biomass and community responses. Arctic and Alpine Research, 28(2): 203-209.

Zhao, J., Tian, L., Wei, H., Sun, F. and Li, R., 2019. Negative responses of ecosystem autotrophic and heterotrophic respiration to experimental warming in a Tibetan semi-arid alpine steppe. Catena, 179: 98-106.

Zhao, Y., Xu, L., Yao, B., Ma, Z., Zhang, C., Wang, F. and Zhou, H., 2016. Influence of Simulated Warming to the Carbon, Nitrogen and Their Stability Isotope-(δ^13^C, δ^15^N)Contents in Alpine Meadow Plant Leaves. Acta Botanica Boreali-Occidentalia Sinica, 36(4): 777-783.

Zhao, Z., Dong, S., Jiang, X., Liu, S., Ji, H., Li, Y., Han, Y. and Sha, W., 2017. Effects of warming and nitrogen deposition on CH_4_, CO_2_ and N_2_O emissions in alpine grassland ecosystems of the Qinghai-Tibetan Plateau. Science of the Total Environment, 592: 565-572.

Zheng, Y., Yang, W., Sun, X., Wang, S.P., Rui, Y.C., Luo, C.Y. and Guo, L.D., 2012. Methanotrophic community structure and activity under warming and grazing of alpine meadow on the Tibetan Plateau. Applied Microbiology & Biotechnology, 93(5): 2193-2203.

Zhu, J., Zhang, Y. and Jiang, L., 2017. Experimental warming drives a seasonal shift of ecosystem carbon exchange in Tibetan alpine meadow. Agricultural & Forest Meteorology, 233(Complete): 242-249.

Zi, H., Hu, L., Wang, C., Wang, G., Wu, P., Lerdau, M. and Ade, L., 2018. Responses of soil bacterial community and enzyme activity to experimental warming of an alpine meadow. European Journal of Soil Science, 69(3): 429-438.

Zong, N., Chai, X., Shi, P.-L. and Yang, X.-C., 2018a. Effects of warming and nitrogen addition on plant photosynthate partitioning in an alpine meadow on the Tibetan Plateau. Journal of Plant Growth Regulation, 37(3): 803-812.

Zong, N., Geng, S., Duan, C., Shi, P., Chai, X. and Zhang, X., 2018b. The effects of warming and nitrogen addition on ecosystem respiration in a Tibetan alpine meadow: The significance of winter warming. Ecology and Evolution, 8(20): 10113-10125.

Zong, N., Shi, P., Jiang, J., Song, M., Xiong, D., Ma, W., Fu, G., Zhang, X. and Shen, Z., 2013. Responses of Ecosystem CO_2_ Fluxes to Short-Term Experimental Warming and Nitrogen Enrichment in an Alpine Meadow, Northern Tibet Plateau. Scientific World Journal.
