# Supplementary material for: Permafrost response to temperature rise in carbon and nutrient cycling: Effects from habitat‐specific conditions and factors of warming
Source: Ecol Evol. 2021 Oct 27;11(22):16021–33. doi: 10.1002/ece3.8271 (PMC8601908; doi:10.1002/ece3.8271)
Supplement: Supplementary file 5 — Table S3 [file ECE3-11-16021-s003.docx]

**Table S3** Data on green leaf N

| **Response** | **Unit** | **Species** | **Vegetation** | **Warming pattern** | **Warming level** | | **Warming time** | | ***Xc*** | ***S_d1_*** | ***n_1_*** | ***Xt*** | ***S_d2_*** | ***n_2_*** | **Citation** |
| --- | --- | --- | --- | --- | --- | --- | --- | --- | --- | --- | --- | --- | --- | --- | --- |
| **variable** |  |  |  |  | ℃ | Level | Growing-season | Time | Control mean | Standrad deviation | Sample size | Treatment mean | Standrad deviation | Sample size |  |
| Green leaf N | % | *Betula nana* | Shrub | Growing-season | 0.3 | Low | 1 | Short | 2.2 | 0.49 | 6 | 2.1 | 0.24 | 6 | Natali etal. (2012) |
| Green leaf N | % | *Carex bigelowii* | Herb | Growing-season | 0.3 | Low | 1 | Short | 2.1 | 0.49 | 6 | 2.2 | 0.24 | 6 | Natali etal. (2012) |
| Green leaf N | % | *Eriophorum vaginatum* | Herb | Growing-season | 0.3 | Low | 1 | Short | 1.7 | 0.24 | 6 | 1.7 | 0.24 | 6 | Natali etal. (2012) |
| Green leaf N | % | *Rhododendron subarcticum* | Shrub | Growing-season | 0.3 | Low | 1 | Short | 1.6 | 0.24 | 6 | 1.6 | 0.49 | 6 | Natali etal. (2012) |
| Green leaf N | % | *Rubus chamaemorus* | Herb | Growing-season | 0.3 | Low | 1 | Short | 2.4 | 0.24 | 6 | 2.3 | 0.24 | 6 | Natali etal. (2012) |
| Green leaf N | % | *Vaccinium uliginosum* | Shrub | Growing-season | 0.3 | Low | 1 | Short | 1.8 | 0.24 | 6 | 1.7 | 0.24 | 6 | Natali etal. (2012) |
| Green leaf N | % | *Betula nana* | Shrub | Growing-season | 0.4 | Low | 2 | Short | 2 | 0.49 | 6 | 2 | 0.24 | 6 | Natali etal. (2012) |
| Green leaf N | % | *Carex bigelowii* | Herb | Growing-season | 0.4 | Low | 2 | Short | 2.1 | 0.24 | 6 | 1.9 | 0.49 | 6 | Natali etal. (2012) |
| Green leaf N | % | *Eriophorum vaginatum* | Herb | Growing-season | 0.4 | Low | 2 | Short | 1.9 | 0.24 | 6 | 1.7 | 0.24 | 6 | Natali etal. (2012) |
| Green leaf N | % | *Rhododendron subarcticum* | Shrub | Growing-season | 0.4 | Low | 2 | Short | 1.6 | 0.24 | 6 | 1.6 | 0.24 | 6 | Natali etal. (2012) |
| Green leaf N | % | *Rubus chamaemorus* | Herb | Growing-season | 0.4 | Low | 2 | Short | 2.2 | 0.24 | 6 | 2.3 | 0.24 | 6 | Natali etal. (2012) |
| Green leaf N | % | *Vaccinium uliginosum* | Shrub | Growing-season | 0.4 | Low | 2 | Short | 1.8 | 0.24 | 6 | 1.8 | 0.24 | 6 | Natali etal. (2012) |
| Green leaf N | % | *Betula nana* | Shrub | Year-round | 0.2 | Low | 1 | Short | 2.2 | 0.49 | 6 | 1.8 | 0.24 | 6 | Natali etal. (2012) |
| Green leaf N | % | *Carex bigelowii* | Herb | Year-round | 0.2 | Low | 1 | Short | 2.1 | 0.49 | 6 | 2.1 | 0.49 | 6 | Natali etal. (2012) |
| Green leaf N | % | *Eriophorum vaginatum* | Herb | Year-round | 0.2 | Low | 1 | Short | 1.7 | 0.24 | 6 | 1.8 | 0.24 | 6 | Natali etal. (2012) |
| Green leaf N | % | *Rhododendron subarcticum* | Shrub | Year-round | 0.2 | Low | 1 | Short | 1.6 | 0.24 | 6 | 1.5 | 0.49 | 6 | Natali etal. (2012) |
| Green leaf N | % | *Rubus chamaemorus* | Herb | Year-round | 0.2 | Low | 1 | Short | 2.4 | 0.24 | 6 | 2.4 | 0.24 | 6 | Natali etal. (2012) |
| Green leaf N | % | *Vaccinium uliginosum* | Shrub | Year-round | 0.2 | Low | 1 | Short | 1.8 | 0.24 | 6 | 1.8 | 0.24 | 6 | Natali etal. (2012) |
| Green leaf N | % | *Betula nana* | Shrub | Year-round | 0.6 | Low | 2 | Short | 2 | 0.49 | 6 | 1.8 | 0.24 | 6 | Natali etal. (2012) |
| Green leaf N | % | *Carex bigelowii* | Herb | Year-round | 0.6 | Low | 2 | Short | 2.1 | 0.24 | 6 | 2.1 | 0.49 | 6 | Natali etal. (2012) |
| Green leaf N | % | *Eriophorum vaginatum* | Herb | Year-round | 0.6 | Low | 2 | Short | 1.9 | 0.24 | 6 | 2 | 0.49 | 6 | Natali etal. (2012) |
| Green leaf N | % | *Rhododendron subarcticum* | Shrub | Year-round | 0.6 | Low | 2 | Short | 1.6 | 0.24 | 6 | 1.6 | 0.24 | 6 | Natali etal. (2012) |
| Green leaf N | % | *Rubus chamaemorus* | Herb | Year-round | 0.6 | Low | 2 | Short | 2.2 | 0.24 | 6 | 2.1 | 0.24 | 6 | Natali etal. (2012) |
| Green leaf N | % | *Vaccinium uliginosum* | Shrub | Year-round | 0.6 | Low | 2 | Short | 1.8 | 0.24 | 6 | 1.8 | 0.05 | 6 | Natali etal. (2012) |
| Green leaf N | % | *Carex stans* | Herb | Year-round | 0.2 | Low | 9 | Long | 2.22 | 0.19 | 3 | 2.11 | 0.19 | 3 | Welker etal. (2004) |
| Green leaf N | % | *Dryas integrifolia* | Shrub | Year-round | 1 | Low | 9 | Long | 1.46 | 0.09 | 3 | 1.3 | 0.12 | 3 | Welker etal. (2004) |
| Green leaf N | % | *Dryas integrifolia* | Shrub | Year-round | 0.2 | Low | 9 | Long | 1.25 | 0.13 | 4 | 1.3 | 0.05 | 4 | Welker etal. (2004) |
| Green leaf N | % | *Dryas integrifolia* | Shrub | Year-round | 0.2 | Low | 9 | Long | 1.64 | 0.08 | 3 | 1.57 | 0.2 | 3 | Welker etal. (2004) |
| Green leaf N | % | *Eriophorum angustifolium* | Herb | Year-round | 1 | Low | 9 | Long | 2.48 | 0.49 | 3 | 2.11 | 0.45 | 3 | Welker etal. (2004) |
| Green leaf N | % | *Salix arctica* | Shrub | Year-round | 0.2 | Low | 9 | Long | 2.15 | 0.16 | 3 | 2.14 | 0.1 | 3 | Welker etal. (2004) |
| Green leaf N | % | *Salix arctica* | Shrub | Year-round | 0.2 | Low | 9 | Long | 2.29 | 0.29 | 4 | 1.95 | 0.46 | 4 | Welker etal. (2004) |
| Green leaf N | % | *Salix arctica* | Shrub | Year-round | 1 | Low | 9 | Long | 2.46 | 0.06 | 3 | 2.07 | 0.15 | 3 | Welker etal. (2004) |
| Green leaf N | % | *Salix arctica* | Shrub | Year-round | 0.2 | Low | 9 | Long | 2.45 | 0.26 | 3 | 2 | 0.13 | 3 | Welker etal. (2004) |
| Green leaf N | % | *Salix arctica* | Shrub | Year-round | 0.2 | Low | 9 | Long | 2.32 | 0.3 | 4 | 2.55 | 0.44 | 4 | Welker etal. (2004) |
| Green leaf N | % | *Betula nana* | Shrub | Growing-season | 2-5 | High | 11 | Long | 2.62 | 0.44 | 5 | 2.36 | 0.51 | 5 | Leffler etal. (2016) |
| Green leaf N | % | *Salix pulchra* | Shrub | Growing-season | 2-5 | High | 11 | Long | 2.69 | 0.42 | 5 | 2.47 | 0.58 | 5 | Leffler etal. (2016) |
| Green leaf N | % | *Dryas integrifolia* | Shrub | Year-round | 1-2 | Low | 17 | Long | 1.7 | 0.68 | 5 | 1.72 | 1.18 | 5 | Hudson etal. (2011) |
| Green leaf N | % | *Salix arctica* | Shrub | Year-round | 1-2 | Low | 17 | Long | 2.72 | 0.76 | 5 | 2.43 | 0.99 | 5 | Hudson etal. (2011) |
| Green leaf N | % | *Oxyria digyna* | Herb | Year-round | 1-2 | Low | 17 | Long | 2.49 | 1.14 | 5 | 3.28 | 0.91 | 5 | Hudson etal. (2010) |
| Green leaf N | % | *Cassiope tetragona* | Shrub | Year-round | 1-2 | Low | 17 | Long | 0.98 | 0.76 | 5 | 0.96 | 0.61 | 5 | Hudson etal. (2011) |
| Green leaf N | % | *Eriophorum angustifolium* | Herb | Year-round | 1-2 | Low | 17 | Long | 2.16 | 0.18 | 5 | 2.4 | 0.19 | 5 | Hudson etal. (2010) |
| Green leaf N | % | *Carex bigelowii* | Herb | Growing-season | 2.2 | High | 1 | Short | 2.1 | 0.49 | 6 | 2.2 | 0.24 | 6 | Natali etal. (2011) |
| Green leaf N | % | *Eriophorum vaginatum* | Herb | Growing-season | 2.2 | High | 1 | Short | 1.7 | 0.24 | 6 | 1.7 | 0.24 | 6 | Natali etal. (2011) |
| Green leaf N | % | *Betula nana* | Shrub | Growing-season | 2.2 | High | 1 | Short | 2.2 | 0.49 | 6 | 2.1 | 0.24 | 6 | Natali etal. (2011) |
| Green leaf N | % | *Rhododendron subarcticum* | Shrub | Growing-season | 2.2 | High | 1 | Short | 1.6 | 0.24 | 6 | 1.6 | 0.49 | 6 | Natali etal. (2011) |
| Green leaf N | % | *Rubus chamaemorus* | Herb | Growing-season | 2.2 | High | 1 | Short | 2.4 | 0.24 | 6 | 2.3 | 0.24 | 6 | Natali etal. (2011) |
| Green leaf N | % | *Vaccinium uliginosum* | Shrub | Growing-season | 2.2 | High | 1 | Short | 1.8 | 0.24 | 6 | 1.7 | 0.24 | 6 | Natali etal. (2011) |
| Green leaf N | % | *Arctostaphylos alpina* | *Shrub* | Growing-season | 2 | Low | 1-3 | Short | 1.28 | 0.28 | 3 | 1.51 | 0.17 | 3 | Welker etal. (2005) |
| Green leaf N | % | *Betula nana* | *Shrub* | Growing-season | 2 | Low | 1-3 | Short | 2.06 | 0.62 | 3 | 2.13 | 0.1 | 3 | Welker etal. (2005) |
| Green leaf N | % | *Dryas octopetala* | *Shrub* | Growing-season | 2 | Low | 1-3 | Short | 1.54 | 0.21 | 3 | 1.82 | 0.1 | 3 | Welker etal. (2005) |
| Green leaf N | % | *Ledum palustre* | *Shrub* | Growing-season | 2 | Low | 1-3 | Short | 0.98 | 0.19 | 3 | 1.16 | 0.25 | 3 | Welker etal. (2005) |
| Green leaf N | % | *Vaccinium vitis-idaea* | *Shrub* | Growing-season | 2 | Low | 1-3 | Short | 1.06 | 0.11 | 3 | 1.03 | 0.13 | 3 | Welker etal. (2005) |
| Green leaf N | % | *Betula nana* | Shrub | Growing-season | 2 | Low | 1-3 | Short | 2.28 | 0.18 | 3 | 2.1 | 0.3 | 3 | Welker etal. (2005) |
| Green leaf N | % | *Eriophorum vaginatum* | Herb | Growing-season | 2 | Low | 1-3 | Short | 2.13 | 0.23 | 3 | 2.22 | 0.18 | 3 | Welker etal. (2005) |
| Green leaf N | % | *Salix pulchra* | Shrub | Growing-season | 2 | Low | 1-3 | Short | 2.33 | 0.26 | 3 | 2.55 | 0.34 | 3 | Welker etal. (2005) |
| Green leaf N | % | *Vaccinium uliginosum* | Shrub | Growing-season | 2 | Low | 1-3 | Short | 1.54 | 0.39 | 3 | 2.04 | 0.34 | 3 | Welker etal. (2005) |
| Green leaf N | % | *Vaccinium vitis-idaea* | Shrub | Growing-season | 2 | Low | 1-3 | Short | 1 | 0.08 | 3 | 1.55 | 0.41 | 3 | Welker etal. (2005) |
| Green leaf N | mg/g | *Cassiope tetragona* | Shrub | Growing-season | 2.8 | High | 5 | Long | 11.2 | 1.47 | 6 | 10.1 | 0.73 | 6 | Michelsen etal.(1996) |
| Green leaf N | mg/g | *Cassiope tetragona* | Shrub | Growing-season | 2.8 | High | 5 | Long | 11.2 | 1.47 | 6 | 10.9 | 1.22 | 6 | Michelsen etal.(1996) |
| Green leaf N | mg/g | *Cassiope tetragona* | Shrub | Growing-season | 2.8 | High | 5 | Long | 12.5 | 0.73 | 6 | 11.6 | 0.98 | 6 | Michelsen etal.(1996) |
| Green leaf N | mg/g | *Cassiope tetragona* | Shrub | Growing-season | 2.8 | High | 5 | Long | 12.5 | 0.73 | 6 | 10.9 | 0.73 | 6 | Michelsen etal.(1996) |
| Green leaf N | mg/g | *Kobresia tibetica* | Herb | Year-round | >1.6 | High | 2 | Short | 23.7 | 1.23 | 10 | 22.1 | 0.98 | 10 | Li etal.(2017) |
| Green leaf N | mg/g | *Carex atrofusca* | Herb | Year-round | >1.6 | High | 2 | Short | 22.5 | 1.11 | 10 | 21.7 | 0.89 | 10 | Li etal.(2017) |
| Green leaf N | mg/g | *Kobresia tibetica* | Herb | Year-round | >1.6 | High | 3 | Short | 23 | 1.11 | 10 | 21.1 | 0.79 | 10 | Li etal.(2017) |
| Green leaf N | mg/g | *Carex atrofusca* | Herb | Year-round | >1.6 | High | 3 | Short | 22.8 | 1.01 | 10 | 21.6 | 0.66 | 10 | Li etal.(2017) |
| Green leaf N | mg/g | *Kobresia tibetica* | Herb | Year-round | >1.6 | High | 4 | Long | 24.2 | 0.76 | 10 | 23 | 0.89 | 10 | Li etal.(2017) |
| Green leaf N | mg/g | *Carex atrofusca* | Herb | Year-round | >1.6 | High | 4 | Long | 24.6 | 1.14 | 10 | 23.1 | 0.89 | 10 | Li etal.(2017) |
| Green leaf N | % | *Kobresia pygmaea* | Herb | Year-round | 1.9 | Low | 4 | Long | 1.74 | 0.1 | 5 | 1.84 | 0.01 | 5 | Zong etal. (2018) |
| Green leaf N | % | *Stipa capillacea Keng* | Herb | Year-round | 1.9 | Low | 4 | Long | 1.72 | 0.07 | 5 | 1.54 | 0.01 | 5 | Zong etal. (2018) |
| Green leaf N | % | *Anaphalis xylorhiza* | Herb | Year-round | 1.9 | Low | 4 | Long | 1.49 | 0 | 5 | 1.55 | 0.05 | 5 | Zong etal. (2018) |
| Leaf *δ*^15^N | ‰ | *Dryas integrifolia* | Shrub | Year-round | 1-2 | Low | 17 | Long | -1.22 | 0.95 | 5 | -0.96 | 1.01 | 5 | Hudson etal. (2011) |
| Leaf *δ*^15^N | ‰ | *Salix arctica* | Shrub | Year-round | 1-2 | Low | 17 | Long | -1.65 | 1.14 | 5 | -1.56 | 1.14 | 5 | Hudson etal. (2011) |
| Leaf *δ*^15^N | ‰ | *Oxyria digyna* | Herb | Year-round | 1-2 | Low | 17 | Long | 2.52 | 1.01 | 5 | 1.65 | 1.01 | 5 | Hudson etal. (2010) |
| Leaf *δ*^15^N | ‰ | *Cassiope tetragona* | Shrub | Year-round | 1-2 | Low | 17 | Long | -5.28 | 1.14 | 5 | -4.77 | 1.27 | 5 | Hudson etal. (2011) |
| Leaf *δ*^15^N | ‰ | *Eriophorum angustifolium* | Herb | Year-round | 1-2 | Low | 17 | Long | 3.49 | 1.08 | 5 | 3.38 | 0.89 | 5 | Hudson etal. (2010) |
| Leaf *δ*^15^N | ‰ | *Carex bigelowii* | Herb | Growing-season | 2.2 | High | 1 | Short | 0.90 | 0.73 | 6 | 1.10 | 0.73 | 6 | Natali etal. (2011) |
| Leaf *δ*^15^N | ‰ | *Eriophorum vaginatum* | Herb | Growing-season | 2.2 | High | 1 | Short | 1.30 | 0.49 | 6 | 1.20 | 0.49 | 6 | Natali etal. (2011) |
| Leaf *δ*^15^N | ‰ | *Betula nana* | Shrub | Growing-season | 2.2 | High | 1 | Short | -7.60 | 1.47 | 6 | -7.30 | 0.73 | 6 | Natali etal. (2011) |
| Leaf *δ*^15^N | ‰ | *Rhododendron subarcticum* | Shrub | Growing-season | 2.2 | High | 1 | Short | -7.30 | 0.98 | 6 | -8.30 | 0.98 | 6 | Natali etal. (2011) |
| Leaf *δ*^15^N | ‰ | *Rubus chamaemorus* | Herb | Growing-season | 2.2 | High | 1 | Short | -0.20 | 0.49 | 6 | 0.20 | 0.24 | 6 | Natali etal. (2011) |
| Leaf *δ*^15^N | ‰ | *Vaccinium uliginosum* | Shrub | Growing-season | 2.2 | High | 1 | Short | 5.90 | 0.49 | 6 | 5.30 | 0.49 | 6 | Natali etal. (2011) |

**References**

Hudson, J.M.G. and Henry, G.H.R., 2010. High Arctic plant community resists 15 years of experimental warming. Journal of Ecology, 98(5): 1035-1041.

Li, F., Peng, Y., Natali, S.M., Chen, K., Han, T., Yang, G., Ding, J., Zhang, D., Wang, G. and Wang, J., 2017. Warming effects on permafrost ecosystem carbon fluxes associated with plant nutrients. Ecology, 98(11).

Natali, S.M., Schuur, E.A.G. and Rubin, R.L., 2012. Increased plant productivity in Alaskan tundra as a result of experimental warming of soil and permafrost. Journal of Ecology, 100(2): 488-498.

Natali, S.M., Schuur, E.A.G., Trucco, C., Pries, C.E.H., Crummer, K.G. and Lopez, A.F.B., 2011. Effects of experimental warming of air, soil and permafrost on carbon balance in Alaskan tundra. Global Change Biology, 17(3): 1394-1407.

Welker, J.M., Fahnestock, J.T., Henry, G.H.R., O'Dea, K.W. and Chimner, R.A., 2004. CO_2_ exchange in three Canadian High Arctic ecosystems: response to long-term experimental warming. Global Change Biology.

Welker, J.M., Fahnestock, J.T., Sullivan, P.F. and Chimner, R.A., 2005. Leaf mineral nutrition of Arctic plants in response to warming and deeper snow in northern Alaska. Oikos, 109(1): 167-177.

Zong, N., Shi, P. and Chai, X., 2018. Effects of warming and nitrogen addition on nutrient resorption efficiency in an alpine meadow on the northern Tibetan Plateau. Soil Science and Plant Nutrition, 64(4): 482-490.
