# Supplementary material for: Permafrost response to temperature rise in carbon and nutrient cycling: Effects from habitat‐specific conditions and factors of warming
Source: Ecol Evol. 2021 Oct 27;11(22):16021–33. doi: 10.1002/ece3.8271 (PMC8601908; doi:10.1002/ece3.8271)
Supplement: Supplementary file 6 — Table S4 [file ECE3-11-16021-s006.docx]

**Table S4** Publication bias assessed with Egger's test

| Response variable | Egger's test |
| --- | --- |
|  | *p* |
| GEP | 0.258 |
| ER | 0.535 |
| NEE | 0.053 |
| AGB | 0.307 |
| BGB | 0.365 |
| Green leaf N | 0.788 |
| Microbial biomass | 0.98 |
| Soil temperature | 0.093 |
| Soil moisture | 0.075 |
| SOC | 0.8 |
| TN | 0.258 |
| Soil NH_4_^+^-N | 0.957 |
| Soil NO_3_^-^-N | 0.449 |

**
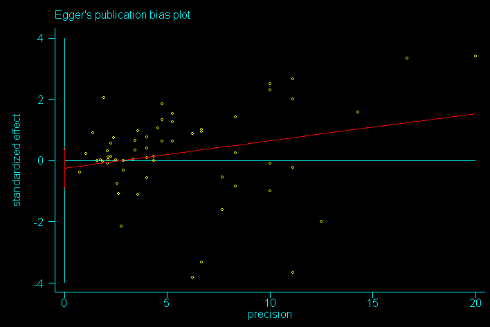
GEP**


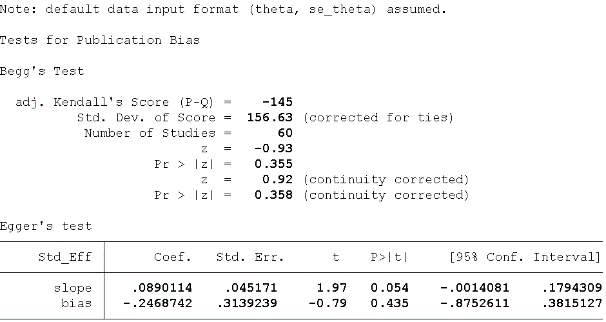


**ER**


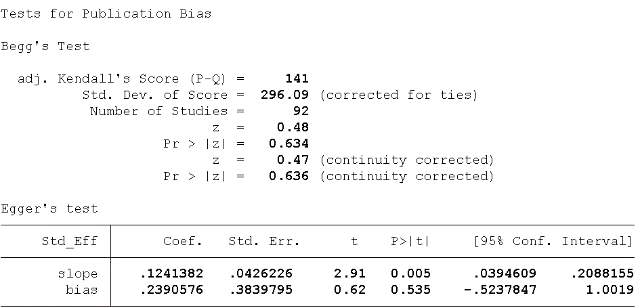

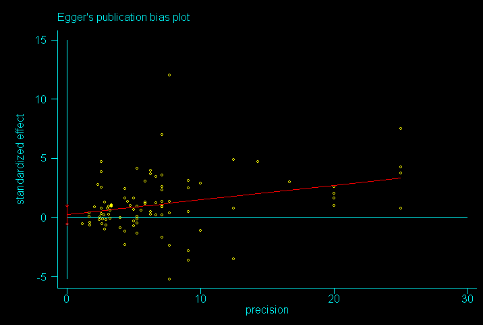


**
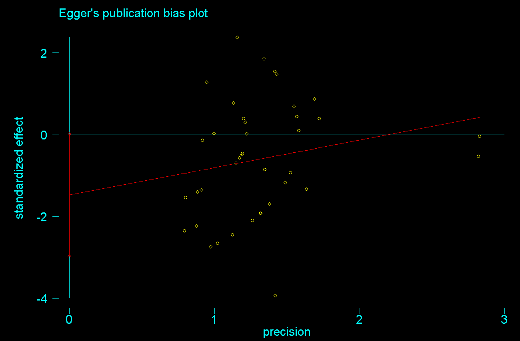
NEE**


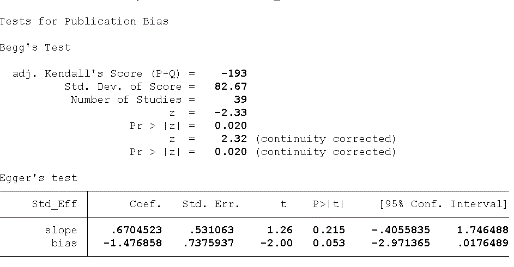


**
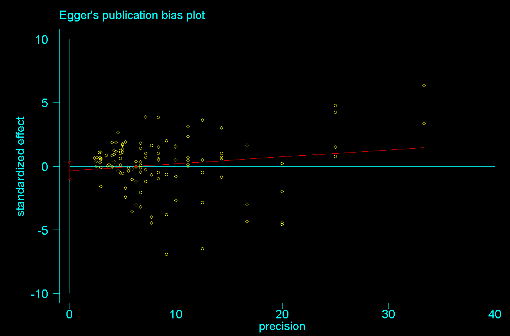
AGB**

**BGB**


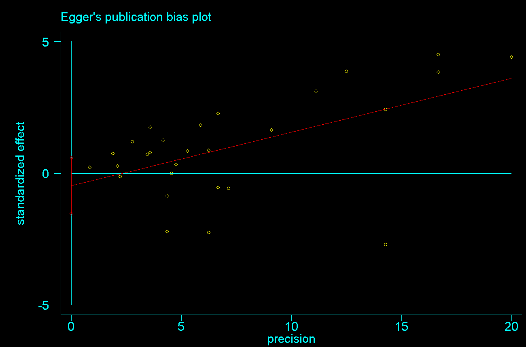


**Green leaf N**


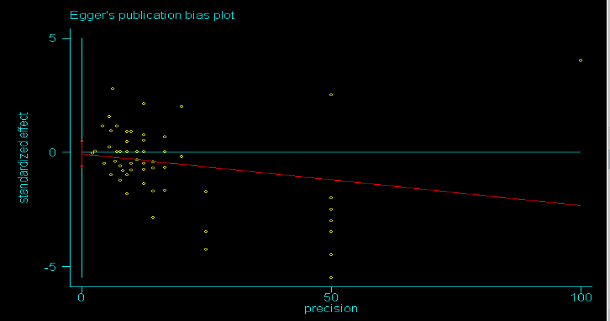


**Microbial biomass**

**
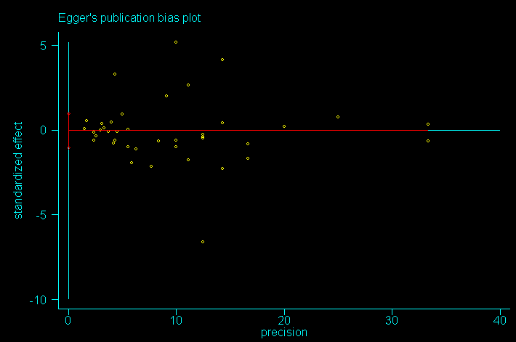
**

**
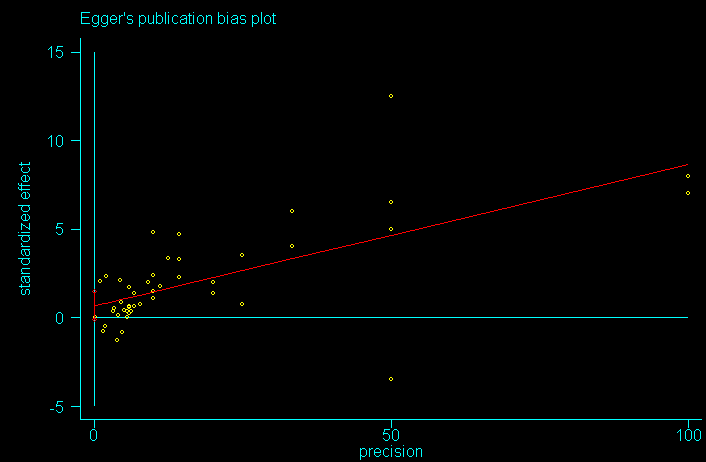
Soil temperature**

**Soil moisture**

**
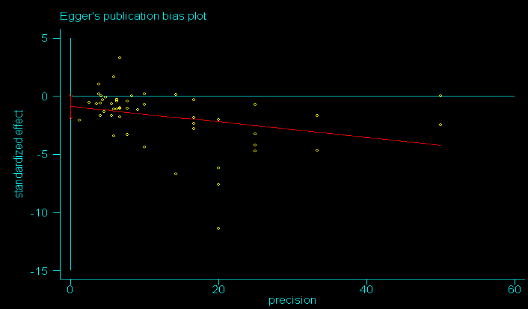
**

**SOC**

**
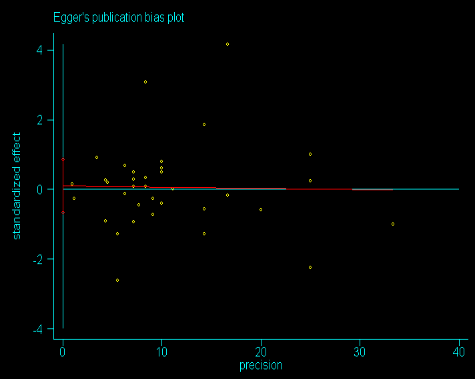
**

**
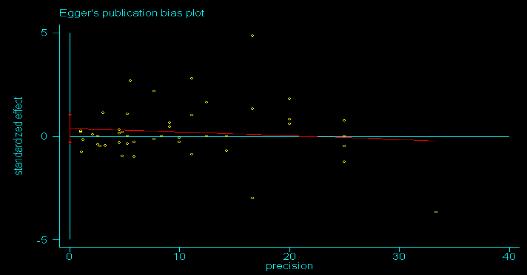
TN**

**Soil NH_4_^+^-N**

**
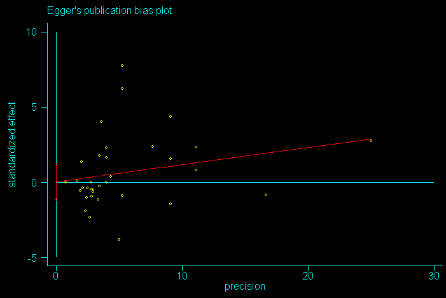
**

**
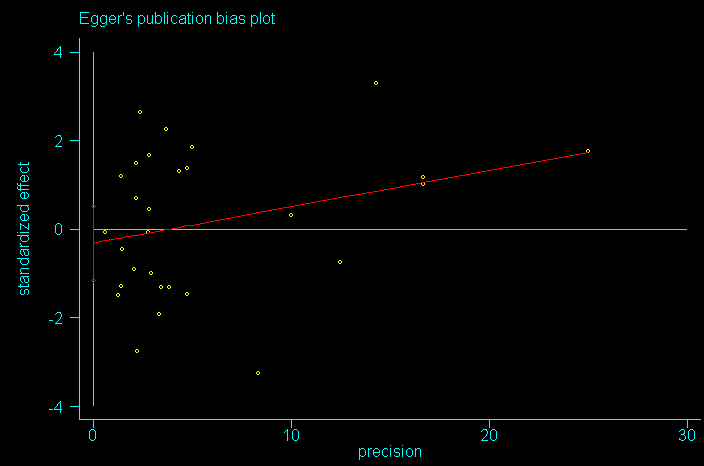
Soil NO_3_^-^-N**
