# Supplementary material for: Permafrost response to temperature rise in carbon and nutrient cycling: Effects from habitat‐specific conditions and factors of warming
Source: Ecol Evol. 2021 Oct 27;11(22):16021–33. doi: 10.1002/ece3.8271 (PMC8601908; doi:10.1002/ece3.8271)
Supplement: Supplementary file 7 — Table S5 [file ECE3-11-16021-s004.docx]

**Table S5** Between-group heterogeneity (*Q_b_*)

| Category | *Q_b_* | *p* |  | Category | *Q_b_* | *p* |
| --- | --- | --- | --- | --- | --- | --- |
| *GEP* |  |  |  | *ER* |  |  |
| Plant functional groups | **18.48** | **<0.001** |  | Plant functional groups | **44.41** | **<0.001** |
| Soil moisture status | 0.87 | 0.648 |  | Soil moisture status | 0.1 | 0.953 |
| Warming pattern | **5.39** | **0.02** |  | Warming pattern | 0.36 | 0.548 |
| Warming level | 1.66 | 0.436 |  | Warming level | **44.54** | **<0.001** |
| Warming time | **23.81** | **<0.001** |  | Warming time | 1.54 | 0.463 |
| *NEE* |  |  |  | *AGB* |  |  |
| Plant functional groups | **12.5** | **0.006** |  | Plant functional groups | **9.27** | **0.01** |
| Soil moisture status | **12.78** | **0.002** |  | Soil moisture status | **21.56** | **<0.001** |
| Warming pattern | 0.15 | 0.70 |  | Warming pattern | **11.63** | **0.001** |
| Warming level | **9.87** | **0.007** |  | Warming level | **94.48** | **<0.001** |
| Warming time | **11.78** | **0.001** |  | Warming time | 1.38 | 0.501 |
| *BGB* |  |  |  | *Green leaf N* |  |  |
| Plant functional groups | 1.9 | 0.386 |  | Plant functional groups | **6.56** | **0.001** |
| Soil moisture status | **6.29** | **0.043** |  | Soil moisture status | **58.36** | **<0.001** |
| Warming pattern | 3.05 | 0.081 |  | Warming pattern | 0.23 | 0.268 |
| Warming level | **26.95** | **<0.001** |  | Warming level | **44.4** | **<0.001** |
| Warming time | **40.62** | **<0.001** |  | Warming time | **7.43** | **0.006** |
| *Soil temperature* |  |  |  | *Soil moisture* |  |  |
| Plant functional groups | 1.97 | 0.374 |  | Plant functional groups | 6.84 | 0.077 |
| Soil moisture status | 3.44 | 0.179 |  | Soil moisture status | **22.6** | **<0.001** |
| Warming pattern | **74.78** | **<0.001** |  | Warming pattern | **4.47** | **0.035** |
| Warming level | **9.37** | **0.009** |  | Warming level | **29.39** | **<0.001** |
| Warming time | **15.65** | **<0.001** |  | Warming time | 5.41 | 0.067 |
| *Microbial biomass* |  |  |  | *SOC* |  |  |
| Plant functional groups | 0.73 | 0.693 |  | Plant functional groups | 0.2 | 0.903 |
| Soil moisture status | 1.84 | 0.398 |  | Soil moisture status | 0.65 | 0.724 |
| Warming pattern | 1.6 | 0.207 |  | Warming pattern | 1.38 | 0.24 |
| Warming level | 5.45 | 0.065 |  | Warming level | 5.65 | 0.059 |
| Warming time | 0.69 | 0.406 |  | Warming time | 0.05 | 0.82 |
| *Soil total N* |  |  |  | *Soil NH_4_^+^-N* |  |  |
| Plant functional groups | 5.66 | 0.13 |  | Plant functional groups | **8.07** | **0.045** |
| Soil moisture status | 5.76 | 0.056 |  | Soil moisture status | **11.06** | **0.004** |
| Warming pattern | 3.29 | 0.07 |  | Warming pattern | **16** | **<0.001** |
| Warming level | **17.95** | **<0.001** |  | Warming level | 2.62 | 0.165 |
| Warming time | **4.36** | **0.037** |  | Warming time | **5.17** | **0.023** |
| *Soil NO_3_^-^-N* |  |  |  |  |  |  |
| Plant functional groups | 2.19 | 0.534 |  |  |  |  |
| Soil moisture status | **10.29** | **0.006** |  |  |  |  |
| Warming pattern | 0.14 | 0.704 |  |  |  |  |
| Warming level | 0 | 0.984 |  |  |  |  |
| Warming time | 0.35 | 0.557 |  |  |  |  |

GEP: gross ecosystem productivity, ER: ecosystem respiration, NEE: net ecosystem C exchange, AGB: aboveground biomass, BGG: belowground biomass, SOC: soil organic C.

The number in bold indicates a significant level at *p*<0.05.
